# A practical guide to the updated seizure classification 2025

**DOI:** 10.1002/epd2.70110

**Published:** 2025-10-13

**Authors:** Sándor Beniczky, Eugen Trinka, Elaine Wirrell, Mamta Bhushan Singh, Hal Blumenfeld, Alicia Bogacz Fressola, Fernando Cendes, Dana Craiu, Birgit Frauscher, Floor E. Jansen, Philippe Kahane, Veena Kander, Nirmeen Kishk, Ching Soong Khoo, Angelica Lizcano, Luca De Palma, Philippe Ryvlin, Nicola Specchio, Michael R. Sperling, William Tatum, Elza Márcia Yacubian, Jo Wilmshurst, J. Helen Cross

**Affiliations:** ^1^ Department of Neurology, Aarhus University Hospital, Member of European Reference Network EpiCARE Aarhus Denmark; ^2^ Department of Clinical Medicine, Aarhus University Aarhus Denmark; ^3^ Department of Clinical Neurophysiology, Danish Epilepsy Centre Dianalund Denmark; ^4^ Department of Neurology, Member of European Reference Network EpiCARE, Center for Cognitive Neuroscience, Christian Doppler University Hospital, Paracelsus Medical University Salzburg Austria; ^5^ Neuroscience Institute, Center for Cognitive Neuroscience, Christian Doppler University Hospital Paracelsus Medical University Salzburg Austria; ^6^ Institute of Public Health, Medical Decision Making and Health Technology Assessment, University for Health Sciences, Medical Informatics, and Technology Hall in Tyrol Austria; ^7^ Divisions of Child and Adolescent Neurology and Epilepsy Department of Neurology Mayo Clinic USA; ^8^ Department of Neurology All India Institute of Medical Sciences New Delhi India; ^9^ Department of Neurology, Neuroscience and Neurosurgery Yale University School of Medicine New Haven Connecticut USA; ^10^ Neurological Institute Universidad de la República (UDELAR) Montevideo Uruguay; ^11^ Department of Neurology, School of Medical Sciences, University of Campinas (UNICAMP) Campinas, São Paulo Brazil; ^12^ Brazilian Institute of Neuroscience and Neurotechnology (BRAINN), Campinas São Paulo Brazil; ^13^ Neuroscience Department, Pediatric Neurology Discipline, “Carol Davila” University of Medicine Bucharest Bucharest Romania; ^14^ Center of Expertise of Rare Pediatric Neurological Disorders “Al. Obregia” Clinical Hospital Bucharest Romania; ^15^ Department of Neurology, Duke University School of Medicine Durham North Carolina USA; ^16^ Department of Biomedical Engineering Duke Pratt School of Engineering Durham North Carolina USA; ^17^ Department of Child Neurology Brain Center, University Medical Center Utrecht the Netherlands; ^18^ Neurology Department, CHU Grenoble Alpes Univ. Grenoble Alpes, Inserm, U1216, Grenoble Institut Neurosciences Grenoble France; ^19^ Department of Pediatric Neurology, Red Cross War Memorial Children's Hospital Neuroscience Institute, University of Cape Town Cape Town South Africa; ^20^ Department of Neurology, School of Medicine, Kasralainy Hospital Cairo University Cairo Egypt; ^21^ Faculty of Medicine, Universiti Kebangsaan Malaysia Kuala Lumpur Malaysia; ^22^ Neurology Unit, Department of Medicine, Hospital Canselor Tuanku Muhriz Kuala Lumpur Malaysia; ^23^ Centre for Global Epilepsy, Wolfson College University of Oxford Oxford UK; ^24^ Department of Clinical Neurophysiology and Epilepsy Clinic, Neurocentro and Coneuro Pereira Colombia; ^25^ Laboratory of Neuroimmunology, Medcare Cúcuta Colombia; ^26^ Neurology, Epilepsy and Movement Disorders Unit, Bambino Gesu’ Children’ Hospital, IRCCS, Member of European Reference Network EpiCARE Rome Italy; ^27^ Department of Clinical Neurosciences, Centre Hospitalier Universitaire Vaudois and Université de Lausanne Lausanne Switzerland; ^28^ Jefferson Comprehensive Epilepsy Center, Department of Neurology, Thomas Jefferson University Philadelphia Pennsylvania USA; ^29^ Department of Neurology Mayo Clinic Jacksonville, Florida USA; ^30^ Department of Neurology and Neurosurgery Universidade Federal de São Paulo São Paulo Brazil; ^31^ University College London NIHR BRC Great Ormond Street Institute of Child Health, Great Ormond Street Hospital & Young Epilepsy London UK

**Keywords:** classification, epilepsy, ILAE, seizure, 2025 update

## Abstract

This paper provides a practical guide to applying the updated seizure classification in clinical settings. The updated classification, published by the International League Against Epilepsy in 2025, builds on the operational classification introduced in 2017. It aims to enhance clarity, clinical relevance, and consistency in seizure terminology across various healthcare settings. The classification system distinguishes between four main seizure classes: focal, generalized, unknown whether focal or generalized, and unclassified. The basic version allows for application even in resource‐limited or primary care environments, while the expanded version offers detailed semiological descriptors and supports advanced diagnostic and surgical decision‐making. Consciousness, defined through awareness (recall) and responsiveness, is now considered a classifier. The paper also highlights the importance of distinguishing observable from non‐observable features and integrates semiology into the expanded classification with attention to temporal sequence and somatotopic detail. Generalized seizures are categorized into seizure types having a direct impact on syndrome diagnosis and treatment decisions. Epileptic spasms are given special attention because of their unique presentation and the urgency of early intervention, particularly in infants. To support clinical implementation, this paper includes a comprehensive table of semiological descriptors, definitions of generalized seizure types, and a series of real‐world case vignettes illustrating the application of the updated seizure classification. Supplementary figures and videos further support the educational aims of the paper. This practical guide is intended for healthcare professionals managing patients with epilepsy, providing a clear, structured approach to seizure classification that is adaptable to varying levels of clinical expertise and diagnostic resources.

## 1 INTRODUCTION

This paper explains how to apply the updated seizure classification in clinical practice. In 2017, the International League Against Epilepsy (ILAE) published a position paper on the operational classification of seizure types.^1^, ^2^ Based on experience gained in real‐world clinical settings, an updated version was released in 2025.^3^, ^4^ For details about how the classification was developed, readers are referred to the ILAE position paper.^3^

The ILAE seizure classification offers a logical, structured framework and standardized terminology, a common language for managing people with epilepsy. Its flexible format, which includes both a basic and an expanded version, makes it practical for different levels of care and expertise, including resource‐limited settings and highly specialized centers. It is useful in both clinical practice and research, and it also supports the classification of epilepsy types and syndromes. Importantly, the seizure classification does not include non‐epileptic paroxysmal episodes, status epilepticus, or neonatal seizures, as these are addressed in separate publications.^5^, ^6^ It also excludes electrographic seizures that occur without clinical manifestations.

The 2017 classification was based on the premise that seizures are divided into four main classes: focal, generalized, unknown, and unclassified. It also introduced the term awareness as a classifier. However, this term proved problematic, both in translation across languages and because it did not account for responsiveness, an essential aspect of consciousness. Additionally, concerns were raised that the 2017 classification did not sufficiently incorporate the evolution of focal seizure semiology over time. Epileptic spasms, particularly important to recognize in infancy due to the need for urgent treatment, were previously included across all seizure classes. However, because the underlying etiology can significantly influence later management, the updated classification includes spasms as a seizure type when generalized and as a descriptor when the seizure is focal — an approach explained in detail in this paper.

Seizures are classified based on all information available at the time of evaluation, and thus the initial classification of the patient's seizure may be revised later if new information about the patient becomes available. While seizure semiology remains the cornerstone of seizure classification, additional insights from paraclinical investigations (such as EEG, neuroimaging, and laboratory tests) can significantly influence the classification. Because of this, seizure classification is context‐sensitive and may evolve over time.

**FIGURE 1:**
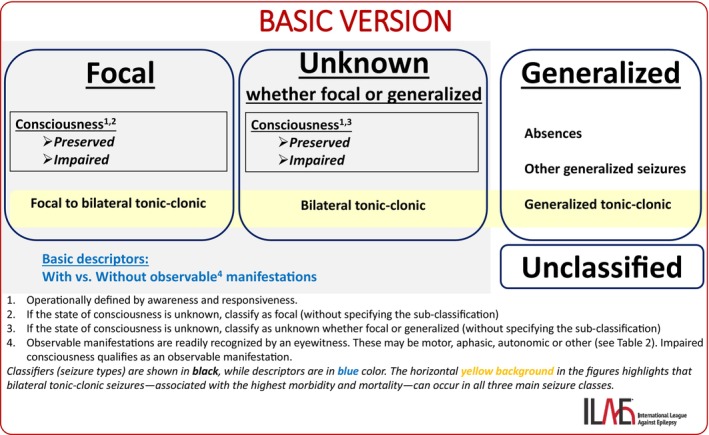
Basic version of the ILAE seizure classification.

Classification is based on the consistent application of principles for grouping and defining seizure types, a system known as *taxonomy*. In seizure classification, the guiding principle is that the categories used (*main seizure classes and seizure types*) should have a direct impact on clinical management, including diagnosis (such as epilepsy type and syndrome classification) and treatment decisions. This approach is primarily justified on operational (or utilitarian) grounds. However, it has also been suggested that these categories reflect underlying biological differences — a conceptual justification. Additional seizure characteristics that support patient management when interpreted alongside other clinical data are included as *seizure descriptors*.

## 2 FOCAL VERSUS GENERALIZED

The main division in seizure classification is between focal and generalized seizures (Box 1).

BOX 1 **Definitions of focal and generalized seizures**

**Table:** 

*Focal seizures* are defined as originating within networks limited to one hemisphere.^1^, ^7^ (Figure 1) They may be discretely localized or more widely distributed, may originate in cortical structures (most often) or subcortical structures (such as periventricular nodular heterotopia and hypothalamic hamartoma). For each seizure type, ictal onset is consistent from one seizure to another, with preferential propagation patterns that may involve the contralateral hemisphere. In some cases, however, there is more than one network, and more than one seizure type, but each individual seizure type has a consistent site of onset.^7^
*Generalized seizures* are defined as originating at some point within, and rapidly engaging, bilaterally distributed networks, which can include cortical and subcortical structures, but not the entire cortex^1^, ^7^ (Figure 1). Semiology at seizure onset can appear localized (focal), can be asymmetric, and EEG features may include focal aspects, but bilateral‐synchronous patterns prevail.^8^, ^9^

Supplementary Material S1 includes an animated infographic that illustrates the conceptual differences between focal and generalized seizures, particularly regarding seizure initiation and evolution.^1^, ^2^, ^7^ This first step in seizure classification has critical clinical implications, as it guides further diagnostic evaluation and influences treatment choices. For focal seizures, identifying and localizing the seizure focus, using neuroimaging and electroclinical correlations, is essential. This is especially important because some focal epilepsies may be amenable to surgical treatment. Classifying seizures as generalized also carries important implications. Certain antiseizure medications that are effective for focal seizures, such as sodium channel blockers, may worsen generalized seizures and should therefore be avoided.^10^

Although distinguishing between focal and generalized seizures can sometimes be challenging, in most cases this can be achieved — even with the relatively limited information available at the primary care level. Generalized seizures typically begin in childhood or adolescence, although they may persist into adulthood. In contrast, seizure onset in adulthood is strongly suggestive of focal seizures, as generalized seizures very rarely (if ever) begin after the age of 25 years.^11^, ^12^, ^13^, ^14^

Clinical features such as unilateral motor or sensory symptoms and focal abnormalities on EEG support a diagnosis of focal seizures. However, bilateral symptoms do not necessarily indicate generalized seizures. Many cortical areas have bilateral body representations, meaning that seizures originating from these regions produce bilateral semiology. Additionally, focal seizures may spread to both hemispheres, resulting in bilateral manifestations.

Conversely, generalized seizures can sometimes manifest focal features at onset (such as head version, focal tonic manifestation/figure‐of‐four, lateralized gestural automatisms^15^, ^16^), followed by rapid engagement of bilateral networks. Moreover, patients with generalized epilepsy may occasionally show focal EEG discharges alongside bilateral‐synchronous epileptiform activity.^15^, ^16^

To improve diagnostic accuracy, all available clinical and paraclinical data should be integrated into the assessment. When uncertainty remains, documentation of seizures, such as through home videos or long‐term video‐EEG monitoring, may be necessary.

## 3 BASIC VERSION

Figure 1 shows the basic version of the updated seizure classification. There are four main seizure classes. As introduced earlier, these include **focal** and **generalized** seizures.

When some information about the seizure is available but not enough to determine whether it is focal or generalized, the seizure is classified as **unknown whether** it is **focal or generalized**. This classification can be updated later if additional data allow a more precise determination.

In cases where no meaningful information about the seizure characteristics is available, but the clinician is confident that the event is epileptic in nature, the seizure is labeled as **unclassified**. If information about a previously diagnosed epilepsy is limited to old medical records that do not include further details, such as seizure type or classification, the seizures should be considered unclassified. Like the “unknown” category, this classification may also change over time as more information becomes available, allowing the seizure to be reclassified appropriately.

In the basic version of the seizure classification, generalized seizures are divided into three categories: *absences, generalized tonic–clonic seizures, and other generalized seizures*. This level of detail is achievable even in primary care settings, where information is often limited and based mainly on the patient history.

Absence seizures are brief (less than one minute) and are characterized by motor and behavioral arrest, impaired responsiveness, and lack of recall. However, some focal seizures can present in a similar fashion,^17^ so an EEG is often necessary to identify the correct seizure type and confirm the diagnosis.

In Figure 1, generalized tonic–clonic seizure is listed as the last item within the seizure class. A horizontal yellow background links it with bilateral tonic–clonic seizures from the other two main classes (focal and unknown). This visual cue highlights that tonic–clonic seizures, regardless of the main seizure class they belong to, carry the highest risk of injury and mortality and represent the most significant risk factor for sudden unexpected death in epilepsy (SUDEP).^18^, ^19^

The third category, other generalized seizures, includes generalized motor seizures other than tonic–clonic seizures. These are typically shorter in duration. Because it is often difficult to distinguish between these seizure types in primary care, they are grouped together in the basic classification.

Focal seizures and seizures unknown whether focal or generalized are further classified based on the patient's state of consciousness during the event — either preserved or impaired. Consciousness is operationally defined by assessing two components: awareness (i.e., recall of the event) and responsiveness. These are evaluated using information from the patient history^20^ or through direct behavioral testing by medical personnel.^21^ If the state of consciousness cannot be determined, the seizure should be classified under the broader category — either as a focal seizure or a seizure of unknown origin.

To improve understanding among patients and caregivers, these terms are explained as the ability to remember and respond appropriately during the seizure. Rather than asking generally about “consciousness,” one should ask specifically whether the patient could recall what happened and respond normally during the seizure. Impaired responsiveness is defined as either an absent or inappropriate response or a significantly delayed reaction compared to the patient's baseline (interictal) state.^18^, ^20^ It is important to remind patients and caregivers that a person's eyes may be open, and they may appear to interact — yet consciousness may still be impaired.

In clinical practice, information may sometimes be available for only one of the two components — awareness or responsiveness. For pragmatic reasons (for example, driving safety), if only one component was tested and is impaired, the seizure is classified as having impaired consciousness. If consciousness is impaired at any point during the seizure, even if it is preserved at the onset, the seizure is classified as having impaired consciousness.

Careful observation and clinical testing during and after the seizure are essential for accurately assessing impairment of consciousness. Both components – awareness and responsiveness – should be assessed when this information is available. If both are impaired, the seizure is classified as impaired. However, if one is preserved and one is impaired, this requires careful consideration for possible preservation of consciousness. Certain conditions can mimic impaired consciousness — for example, isolated epileptic amnesia may explain impaired recall, while ictal paresis or ictal receptive aphasia may cause apparent unresponsiveness.^22^, ^23^, ^24^ These possibilities should be carefully considered and ruled out when feasible. It is also important to note that impairment of consciousness, as opposed to a complete loss, suggests that some elements of consciousness may be preserved. However, one must differentiate this from localized neurological deficits, such as ictal or postictal aphasia, palsy, or amnesia, which may affect responsiveness or recall independently of consciousness itself. For more details about the evaluation of consciousness during seizures based on assessment of awareness and responsiveness, see Supplementary Material S2.

Earlier editions of the ILAE seizure classification also used consciousness as a classifier.^25^ In the 1981 classification, focal seizures with impaired consciousness were termed complex partial seizures, while those with preserved consciousness were called simple partial seizures.^26^ However, these terms were not self‐explanatory. In the 2017 classification, awareness was introduced as a surrogate for consciousness.^1^, ^2^ However, the term awareness poses challenges in translation (limiting its usefulness in international clinical contexts), and it was defined narrowly as the ability to recall events, which does not fully align with how consciousness is assessed in routine neurological practice. The updated seizure classification addresses these concerns by using self‐explanatory terms for focal seizures with impaired or preserved consciousness, and by including both awareness and responsiveness in the definition, in alignment with other neurological disorders of consciousness.

Changes in consciousness are also important in the classification of generalized seizures. This is implicitly reflected in the names of generalized seizure types. By definition, both absence seizures and generalized tonic–clonic seizures involve impaired consciousness. However, the severity of impaired consciousness can be variable even in generalized seizures.^27^, ^28^, ^29^

Focal seizures can propagate to involve bilateral networks and evolve into bilateral tonic–clonic seizures. This seizure type is called a focal‐to‐bilateral tonic–clonic seizure^1^, ^2^, ^3^ — previously referred to as secondarily generalized tonic–clonic seizure in the 1981 classification.^26^ Consciousness is impaired during these seizures.

To further characterize seizures, semiological descriptors can be added to focal seizures and to seizures unknown whether focal or generalized. This is not necessary for generalized seizures, as the seizure type name already implies the key semiological features.

In the basic version of the classification, a simple dichotomy is used for descriptors: seizures are categorized as either having observable manifestations or no observable manifestations. Observable semiology involves involuntary features that may include motor, aphasic, autonomic, or other signs identifiable by eyewitnesses.^30^ Seizures with impaired consciousness are, by definition, considered to have observable manifestations. Motor and behavioral arrest are also considered observable manifestations. This distinction is particularly valuable in the context of clinical trials.^30^ It also emphasizes an important clinical point: seizures can occur without visible manifestations, a fact that helps reduce diagnostic delays and supports earlier identification and treatment.

## 4 EXPANDED VERSION

**FIGURE 2:**
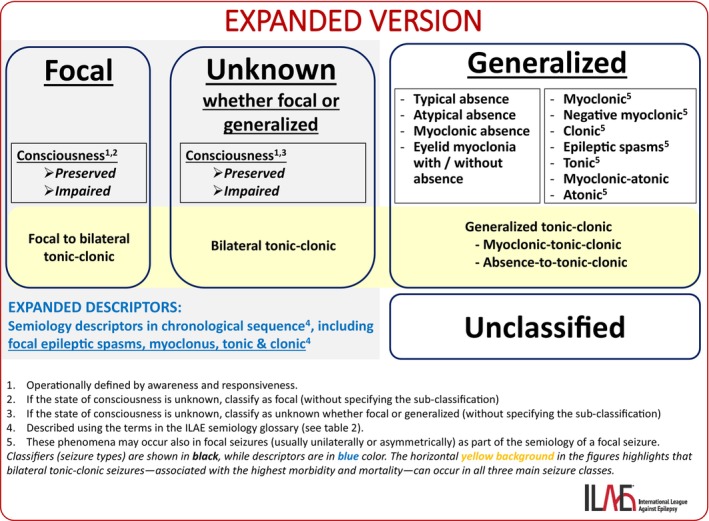
Expanded (advanced) version of the ILAE seizure classification.

In the expanded version of seizure classification (Figure 2), seizure semiology is described in greater detail. Semiology features are listed in the chronological order in which they occur during the seizure,^17^ as originally proposed by Lüders et al. in the semiological seizure classification.^31^, ^32^ Table 1 provides a comprehensive list of semiological features, including motor phenomena (both elementary and complex), sensory, cognitive, and language‐related features, as well as autonomic, emotional (affective), and postictal phenomena. Some features are inherently linked to specific body regions — for example, an epigastric aura is associated with the abdominal area. However, for most semiological features, it is important to specify both the side (right or left) and the body part involved — for example, automatisms in the right hand. This is called somatotopic modifier (Table 1).

**FIGURE 3:**
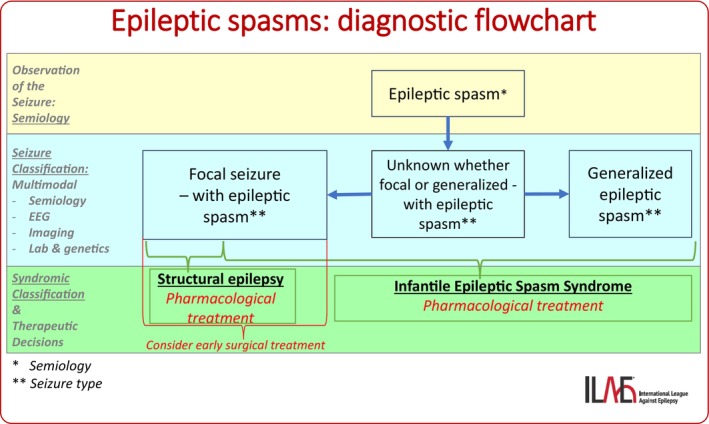
Diagnostic flowchart for epileptic spasms.

**TABLE 1:** Descriptors for Focal seizures and for seizures Unknown whether Focal or Generalized.

Somatotopic modifiers	
Side (left, right, bilateral‐symmetric, bilateral‐asymmetric) + Body part	
Semiology features	
**1. Elementary motor phenomena** ^**a**^ Akinetic Astatic Atonic Clonic Dystonic Epileptic nystagmus Epileptic spasm Eye blinking Eye deviation Gyratory Head orientation Ictal paresis Motor and behavioral arrest Myoclonic Myoclonic‐atonic Epileptic negative myoclonus Tonic (focal tonic, chapeau de gendarme, fencing posture) Tonic–clonic (Figure‐of‐four) Versive **2. Complex motor phenomena** ^**a**^ Automatisms Gestural automatisms‐distal Gestural automatisms‐genital Gestural automatisms‐proximal Ictal grasping Mimic automatisms (Gelastic, dacrystic) Oroalimentary automatisms Verbal automatisms Vocal automatisms Hyperkinetic behavior **3. Sensory phenomena** ^**b**^ Auditory Body‐perception illusion Depersonalization Gustatory Olfactory Somatosensory painful non‐painful Vestibular/Dizziness Visual **4. Cognitive & language phenomena** ^**c**^ Aphasia Confusion/disorientation Dysmnesia Amnesia Déjà vu/déjà vécu/jamais vu/dreamy state /reminiscence Forced thinking Other focal cognitive deficits (e.g., anosognosia, apraxia, neglect)	**5. Autonomic phenomena** ^**c**^ Cardiovascular Ictal asystole Ictal bradycardia Ictal tachycardia Cutaneous/thermoregulatory Flushing Piloerection Sweating Epigastric Gastrointestinal Borborygmi Flatulence Hypersalivation Nausea/Vomiting Polydipsia Sialorrhea Spitting Pupillary Miosis Mydriasis Respiratory Apnea Choking Hyperventilation Hypoventilation Urinary Incontinence Urinary urge **6. Affective (emotional) phenomena** ^**#**^ Anger Anxiety Ecstatic/bliss Fear Guilt Mirth Mystic Sadness Sexual **7. Indescribable aura** ^**b**^ **Postictal phenomena** Autonomic signs Blindness (hemianopsia or amaurosis) Confusion Headache Language dysfunction Nose wiping Palinacousis Paresis (Todd's paresis) Psychiatric signs Unresponsiveness

*Note*: If phenomena not listed above occur during the seizure, they are added in free text. Awareness and responsiveness define consciousness and hence are classifiers. All items in this table are defined in the ILAE glossary of semiology.

^a^ Observable manifestations.

^b^ Not observable manifestations.

^c^ Possibly observable manifestations.

The sequence of semiological features is indicated using arrows (➔) to show the direction of seizure evolution. Semiology features that occur simultaneously are connected in the description with a plus sign (+). For example: epigastric aura ➔ left‐hand automatism + right upper limb dystonia ➔ impaired responsiveness + impaired awareness. Understanding ictal evolution is clinically valuable, as it can help identify specific epilepsy syndromes, such as epilepsy of infancy with migrating focal seizures,^33^ and assist in localizing the cortical regions responsible for seizure generation.^17^ To obtain an accurate and detailed description of the seizure, video documentation or ideally video‐EEG monitoring is often needed. This is particularly important in the presurgical evaluation of patients with drug‐resistant focal epilepsy.

It is also important to note that colloquial terms derived from semiology, such as hyperkinetic (or hypermotor) seizures, focal spasms, focal myoclonic seizures, focal clonic seizures, and focal tonic seizures, refer to focal seizures as the underlying seizure type.

For a more detailed explanation of seizure semiology, readers are referred to the ILAE semiology glossary.^17^ This resource provides clear definitions and video examples of various semiological features and offers an in‐depth discussion of their value in lateralizing and localizing focal seizures.

For focal seizures and seizures of unknown origin, the difference between the basic and expanded versions of the classification lies in the granularity of the seizure descriptors (as discussed above). These additional descriptors provide more detail but do not influence the choice of antiseizure medication, nor are they, on their own, sufficient to guide decisions about surgical treatment. As such, they are considered descriptors, not classifiers.

In contrast, for generalized seizures, the expanded version lists specific seizure types. These have a direct impact on syndrome diagnosis and carry therapeutic implications. We provide in Box 2 the definitions of the generalized seizure types, from the ILAE position paper.^3^

**FIGURE 4:**
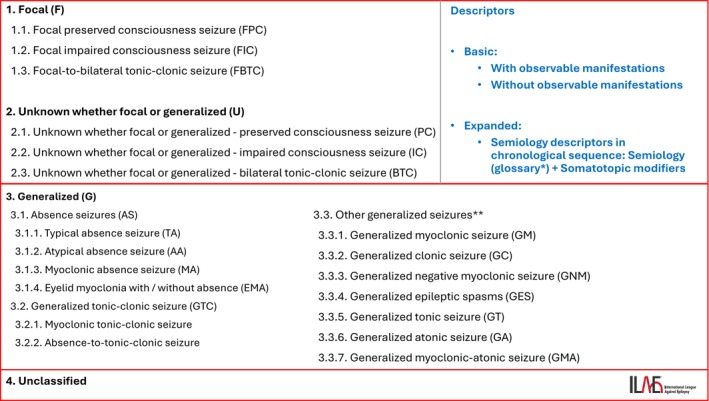
List of the seizure classes and seizure types.

The seizure taxonomy, including the main classes and seizure types, is summarized in Figure 4. The updated classification comprises four main classes and 21 seizure types. Translations are available in 14 different languages.^3^ A recently published paper provides a translation table to match equivalent terms across the 1981, 2017, and 2025 ILAE seizure classifications.^34^

BOX 2 **Definitions of the generalized seizure types.^3^**

**Table:** 

*Typical absence seizure* is a generalized epileptic seizure characterized by sudden onset, interruption of ongoing activities, a blank stare (loss of facial expression), and possibly a brief upward deviation of the eyes. Usually the patient is unresponsive; in most cases, awareness is impaired too. However, occasionally, after the seizure, patients may recall the ictal events (for example test words given during the seizure). Oral and/or manual automatisms occur in 86% of patients and eye involvement with blinking, eye opening, or subtle eyelid or perioral myoclonus in 76.5% of patients. There is immediate return to normal activity, although children may be momentarily confused as they reorient themselves. Duration is a few seconds to half a minute (median 7 s; range: 2–26 s), but rarely they may last >30 s. Ictal EEG is characterized by regular, bilateral‐synchronous (“generalized”) spike–waves. In the first seconds of seizure onset, the frequency of the spike–waves is around 3 Hz; range: 2.5–4 Hz in childhood absence epilepsy (CAE), 3–5.5 Hz in juvenile absence epilepsy (JAE). Disorganized discharges, defined by brief (<1 s) or transient interruptions in the ictal rhythm, or waveforms of different frequency or morphology are significantly less common in CAE than in JAE. The seizures are typically provoked by hyperventilation in most untreated patients with CAE. They may be provoked by intermittent photic stimulation too. In CAE, seizures typically occur multiple times per day but are often under‐recognized. In JAE, typical absence seizures occur less than daily in the untreated state.
*Atypical absence seizure* is a generalized seizure type characterized by episodes of impaired consciousness (awareness and/or responsiveness). Changes in tone (when present) are more pronounced than in typical absence seizures (for example head‐drop as opposed to mild head retropulsion), and the onset and/or cessation is gradual (not abrupt). Duration is usually longer than that of typical absence seizures, but with considerable overlap (median: 15 s; range: 2–10 s). Ictal EEG shows irregular, bilateral‐synchronous and asynchronous/asymmetric spike–waves, with frequency lower than in typical absence (<2.5 Hz) and the ictal activity may be fragmented or include fast activity. Atypical absence seizures may occur in Lennox–Gastaut syndrome.
*Myoclonic absence seizure* is a type of absence seizure with abrupt onset and offset, associated with rhythmic 3‐Hz jerks of the upper limbs, superimposed on tonic abduction of the arms during the seizure (giving a ratcheting appearance). The patient, if standing, typically bends forward during the seizure, but falling is uncommon. The myoclonic jerks are typically bilateral and symmetric but can be unilateral or asymmetric. Perioral myoclonia and rhythmic jerks of the head and legs may also occur. Impairment of consciousness varies from complete loss of awareness and responsiveness to retained awareness and responsiveness. Occasionally, autonomic manifestations, such as a change in breathing or urinary incontinence, or complex gestural automatisms, may be seen. Duration is typically 7–12 s, but occasionally longer (up to 60 s) and may occur multiple times per day. Ictal EEG shows regular 3 Hz, bilateral‐synchronous (“generalized”) spike–waves, time‐locked with the myoclonic jerks. Myoclonic absence seizures occur in a variety of genetic conditions; this seizure type is mandatory for the diagnosis of epilepsy with myoclonic absence syndrome. Polygraphic recordings of EMG with EEG are recommended for ictal recordings.
*Eyelid myoclonia with/without absence*. Eyelid myoclonia, consists of brief, repetitive, and often rhythmic 3–6‐Hz myoclonic jerks of the eyelids, often with simultaneous upward deviation of the eyeballs and extension of the head. Eyelid myoclonia can be associated with absences, but can occur without a corresponding absence. They are typically induced by involuntary or voluntary slow eye closure or exposure to bright sunlight. These seizures are very brief (median duration: 1.5 s; range: 0.5–8 s) and occur multiple times each day, even many times per hour. Ictal EEG shows bilateral‐synchronous (“generalized”) fast spike activity or 3–6 Hz polyspike‐and‐wave discharges, typically elicited by eye closure and intermittent photic stimulation, especially in untreated patients. This seizure type is mandatory for the diagnosis of epilepsy with eyelid myoclonia (formerly called Jeavons syndrome).
*Generalized tonic–clonic seizure* consists of a tonic phase, with sustained muscle activity, followed by a clonic phase with progressive slowing of the clonic jerks, due to the gradual increase in the duration of the silent‐periods interrupting the muscle activation, which eventually terminates the seizure. These motor phenomena are bilateral, but not always symmetric, and focal features (such as forced head version) may be observed in generalized tonic–clonic seizures. Typically, there is loss of consciousness during the seizure and in the postictal period. GTC seizures may be preceded by sporadic or irregular myoclonic jerks (myoclonic‐tonic–clonic seizure) often seen in patients with juvenile myoclonic epilepsy, or by an absence seizure (absence‐to‐bilateral‐tonic–clonic seizure). Median duration of GTCS is 80 s (range: 57–102 s). The ictal EEG is often obscured by movement artifact. Bilateral‐synchronous (“generalized”) fast rhythmic spikes may be seen in the tonic stage, which is followed by bursts of spikes and slow waves, synchronous with clonic jerks, during the clonic phase. A postictal period of generalized EEG suppression (PGES) or irregular, diffuse slow activity follows a GTC seizure. In idiopathic generalized epilepsies, GTC seizures often occur on awakening or with sleep deprivation. In focal conditions, the seizure is classified as focal‐to‐bilateral tonic–clonic seizure (FBTC). When the origin is unknown, the seizure is classified as bilateral tonic–clonic seizure, unknown whether focal or generalized (BTC). Tonic–clonic seizures have the highest associated morbidity and mortality, and represent the major risk factor of SUDEP.
*Generalized myoclonic seizure*. Myoclonic jerks (a.k.a. myoclonus, plural: myoclonia) were defined as sudden, brief (lightening‐like; <100‐msec) involuntary, single or multiple irregular/arrhythmic contractions of muscles or muscle groups. When measured using surface electromyogram (EMG), their median duration was 80 ms (range: 30–140 ms). Generalized myoclonic seizures are bilateral but can predominate on one side of the body, frequently involving the upper extremities. They can also involve the lower limbs and cause falls. Generalized myoclonic seizures can be reflex, triggered by photic stimulation, or praxis. The typical ictal EEG correlate is bilateral‐synchronous (“generalized”) polyspike‐and‐wave discharges (or spike‐and‐wave discharge), time‐locked to the myoclonus. Generalized myoclonic seizures are mandatory for the diagnosis of juvenile myoclonic epilepsy, and they may occur in other generalized epilepsies too. Note that unilateral myoclonic jerks can occur in focal seizures, in which case they are classified as focal seizures, and myoclonus is added as a descriptor of seizure semiology.
*Generalized clonic seizure* consists of myoclonic jerks that are regular and repetitive, at a relatively low frequency (typically 0.2–5 Hz) and involve the same muscle groups. Generalized clonic seizures are bilateral, but not always synchronous and symmetric. Duration is 4 s (range: 1–24 s). Ictal EEG shows generalized spike‐and‐wave or polyspike‐and‐wave discharges, time‐locked to the clonic jerks. Note that unilateral or asymmetric clonic phenomena can occur in focal seizures, in which case they are classified as focal seizures, and clonic is added as a descriptor of seizure semiology. Generalized clonic seizures should be distinguished from myoclonic absence seizures, which exhibit distinct movement patterns.
*Generalized negative myoclonic seizure* is defined as a brief interruption of muscle tone (<500 ms), causing a sudden, brief lapse in movement that may grossly appear like a myoclonic jerk. Generalized negative myoclonic seizures are bilateral, but not always synchronous and symmetric. To document negative myoclonus, it is often necessary to instruct the patient to perform a voluntary muscle activation, such as lifting the arms. The EEG correlate is a spike–wave or a low‐amplitude sharp‐transient. The onset of the EMG silent‐period is related to a negative component of the spike on the EEG, occurring before the slow wave. In progressive myoclonic epilepsies, a cortical involvement has been demonstrated in cortical reflex negative myoclonus. Unilateral or asymmetric negative myoclonus can occur in focal seizures, in which case they are classified as focal seizures, and negative myoclonus is added as a descriptor of seizure semiology. Subcortical negative myoclonus may occur in metabolic encephalopathies.
*Generalized epileptic spasms* consist of brief contractions of axial (predominantly truncal and proximal) muscles, each typically lasting ≤2 s (median: 1 s; range: 0.4–2 s), causing abduction and extension of both arms, hip flexion, and nodding. Subtle forms of spasms, with minimal/discrete manifestations may occur, including head nodding, grimacing, smiling, or chin movement. Epileptic spasms usually occur in clusters, often upon awakening, with increasing prominence of the motor features through the cluster, often over a period of minutes (although clusters may last 30 min or longer). The ictal EEG correlate is characterized by a high‐amplitude, generalized, sharp or slow wave that is followed by low amplitude, fast activity, or a brief, diffuse electrodecrement. Surface EMG helps to distinguish epileptic spasms from myoclonic seizures and tonic seizures. The EMG of an epileptic spasm has a typical diamond shape (gradual increase and gradual decrease in amplitude). Epileptic spasms are mandatory for the diagnosis of infantile epileptic spasms syndrome (IESS). Epileptic spasms may occur in focal/structural epilepsies, in which case they may appear unilateral or asymmetric. However, the bilateral symmetric semiology does not rule out the focal origin, and a complex multimodal investigation, including video‐polygraphic recordings, neuroimaging, laboratory, and genetic tests are needed to correctly classify epileptic spasm. When epileptic spasm occurs in a focal condition, it is classified as focal seizure, and epileptic spasm is added as a descriptor of seizure semiology. When the origin is uncertain, epileptic spasm is classified as unknown whether focal or generalized. While epileptic spasms can be generalized, focal, or of unknown origin, the most critical factor in infants is their early recognition and prompt initiation of spasm‐specific therapies, as treatment delays are associated with poorer developmental outcomes. Determining whether spasms are focal or generalized can be challenging (Figure 3) and often requires a multimodal approach. When interpreted in the context of clinical data — particularly the age of onset — spasms typically lead to a syndromic diagnosis of IESS. In such cases, syndrome‐specific pharmacological treatment must be started without delay. In some cases, spasms may present with unilateral or asymmetric features, suggesting a focal origin. When this is supported by additional findings, such as neuroimaging, and if first‐line treatments fail, early surgical evaluation should be considered (Figure 3). Importantly, epileptic spasms can also occur outside infancy, in older age groups. This broader age range is reflected in the shift in terminology from infantile spasms to epileptic spasms. In these cases, the pharmacological treatment approach differs from that used for IESS (Figure 3).
*Generalized tonic seizure* is defined as sustained muscular contraction resulting in stiffness or tense posture, that usually causes an extension, but it may also affect the flexor muscles. The median duration of generalized tonic seizures is 8 s (range: 3–51 s). Generalized tonic seizures are bilateral, but not necessarily symmetrical. They may be subtle, with slow upward eye rolling or deviation, at times with facial grimace or flexor movements of the head and/or trunk, or more clinically obvious, with a brief cry, apnea, abduction, and elevation of the limbs with a vibratory component and bilateral fist clenching. If occurring while the patient is standing, they may forcefully throw the patient off balance, leading to a fall, with the patient often sustaining an injury. Tonic seizures can be precipitated by startle. During sleep, generalized tonic seizures may be very subtle and not recognized by the family members, and therefore need polygraphic recording of sleep to identify and quantify seizure frequency. Generalized tonic seizures may be preceded or followed by spasms (colloquially termed “tonic spasms”), a myoclonic jerk (“myoclonic‐tonic seizure”), or a hyperkinetic seizure followed by a spasm (“hypermotor‐tonic‐spasms”). The ictal EEG pattern of tonic seizures consists of a burst of bilateral 10 Hz or higher frequency fast activity with a recruiting rhythm, an initial diffuse decrement followed by a gradual increase in amplitude. Generalized tonic seizures are mandatory for diagnosis of the Lennox–Gastaut syndrome. Focal tonic ictal phenomena may occur in focal seizures, in which case they are classified as focal seizures, and tonic is added as a descriptor of the seizure semiology.
*Generalized atonic seizure* is defined as a sudden loss or decrease in muscle tone, without apparent preceding myoclonic or tonic event, involving the head, trunk, jaw, and limbs. Due to the loss of postural tone, atonic seizures frequently cause falls and injury. The median duration is 1 s (range: 0.5–13 s). Ictal EEGs typically show generalized electrodecrement or slow waves. Polygraphic recordings including surface EMG of the antagonist muscles are useful to document this seizure type. Atonic seizures are often observed in Lennox–Gastaut syndrome. They may occur in focal epilepsies too, in which case they are classified as focal seizures and atonic is added as a descriptor of the seizure semiology.
*Generalized myoclonic‐atonic seizure* is characterized by a brief myoclonic jerk affecting the proximal muscles, often associated with a slight vocalization, followed by a very brief atonic component, which may be subtle, with a head nod, or more prominent, with an abrupt fall. Median duration is 1.25 s (range: 0.7–1.5 s). Ictal EEG shows bilateral‐synchronous (“generalized”) polyspike or spike discharges with the myoclonus, followed by a high‐voltage slow wave accompanying the atonic component. Polygraphic recordings of EMG with EEG are recommended for ictal recordings. Myoclonic–atonic seizures are mandatory for diagnosis of Epilepsy with Myoclonic‐Atonic Seizures (Doose syndrome).

## 5 CLINICAL VIGNETTES

To illustrate the application of seizure classification in clinical practice, we present a series of case vignettes. In addition, readers are referred to the examples provided in the ILAE position paper^3^ for further guidance.

- A 7‐year‐old boy had been experiencing brief spells for the past six months. He had normal, age‐appropriate intellectual functioning but began to underperform at school following the onset of his seizures. He had only one seizure type, which could be easily triggered by hyperventilation. During the episode, he stopped hyperventilating, followed by mild upward eye deviation, slight head retropulsion, and rhythmic, slow eye blinks (Supplementary Material S3). He was unresponsive during the episode but immediately followed commands afterward. EEG showed generalized 3 Hz spike–waves (Supplementary Material S3). This seizure is classified as a typical absence seizure.
- An 8‐year‐old boy presents with a three‐year history of blank spells. According to his family, he has multiple daily episodes characterized by a sudden onset, with his eyes rolling back and brief unresponsiveness lasting a few seconds. These episodes interrupt ongoing activities, after which he resumes them as if nothing had happened. More recently, the episodes have been accompanied by a sudden loss of tone, causing him to slump forward, occasionally resulting in minor injuries. Video‐EEG recording shows bilateral‐synchronous 2 Hz spike–waves (Supplementary Material S4). Based on the EEG (<2.5 Hz generalized spike–waves) and the marked atonic phenomenon during the episode, this seizure is classified as atypical absence.
- A 4‐year‐old girl with learning difficulties presented with multiple daily clusters of jerking episodes, first noted between the ages of 2 and 3 years. On observation during admission, she exhibited recurrent, rhythmic myoclonic jerks causing a ratcheting upward movement of the upper limbs, accompanied by flexion of the head. EEG showed bilateral‐synchronous 3 Hz spike‐and‐wave discharges. She immediately resumed activity once the discharges ended (Supplementary Material S5). This seizure is classified as a myoclonic absence seizure.
- An 8‐year‐old girl with normal development and intellect recently has been observed having brief episodes lasting 5–10 seconds, occurring multiple times per day. Her family reported that she would suddenly stop, stare, become unresponsive, and occasionally exhibit upward eye deviation. During EEG recording, hyperventilation immediately provoked these episodes, which were associated with generalized 3 Hz spike–wave discharges. While performing a repetitive motor task, she exhibited arrest of movement and was unresponsive during the events (Supplementary Material S6). This seizure is classified as a typical absence seizure.
- A six‐month‐old boy with an unremarkable pre‐ and perinatal history had normal development until the age of five months, when he began experiencing brief (up to 2 seconds) episodes of bilateral, symmetrical contractions of the limbs. These involved flexion of the legs, extension of the arms, and contraction of facial muscles (Supplementary Material S7). The episodes occurred primarily upon awakening, in clusters of 10–25 contractions. Following seizure onset, the child's development plateaued. Interictal EEG revealed a slow background pattern and multifocal sharp and spike–wave discharges. These seizures are classified as generalized epileptic spasms.
- A 4‐year‐old boy presented with frequent, uncontrollable bouts of inappropriate laughter, sometimes accompanied by frightened facial expressions and fist clenching. According to his mother, he did not respond when spoken to during the episodes, which occurred multiple times per day. His development was within normal limits, although behavioral problems were reported. EEG was inconclusive, but MRI revealed a hypothalamic hamartoma. This seizure is classified as a focal impaired consciousness seizure, with the following semiology: gelastic ➔ impaired responsiveness.
- A 3‐year‐old girl presented with daily episodes of brief staring accompanied by rhythmic jerks of the upper limbs and head, lasting 5–10 seconds. EEG during the episodes revealed 3 Hz spike‐and‐wave discharges. Her developmental milestones were mildly delayed. Genetic testing confirmed a diagnosis of GLUT1 deficiency syndrome. Seizures persisted despite treatment with valproate but significantly decreased within weeks of starting a ketogenic diet, which also led to improved alertness. This seizure is classified as a myoclonic absence seizure.
- A 7‐year‐old right‐handed boy has experienced recurrent afebrile seizures since the age of six. The episodes, as described by his parents, involved behavioral arrest, unresponsiveness, and lip‐smacking automatisms (Supplementary Material S8), followed by postictal sleepiness. At the age of 9 months, the patient had prolonged febrile seizures. During video‐EEG monitoring, a seizure with the reported semiology was captured. In addition, the recording showed slight eye and head deviation to the left, rhythmic non‐clonic hand movements on the right, and verbal automatisms. Neuroimaging revealed mesial temporal sclerosis on the left side (Supplementary Material S8). This seizure is classified as a focal impaired consciousness seizure, with the following semiology: behavioral arrest + staring ➔ oroalimentary automatism + rhythmic non‐clonic hand movement on the right ➔ verbal automatism + unresponsiveness to verbal/tactile stimuli ➔ postictal somnolence.
- A 15‐year‐old girl presented with recurrent episodes of behavioral arrest. A habitual seizure was captured during video‐EEG recording (Supplementary Material S9). The patient reported a rising epigastric sensation and a sense of fear, followed by automatisms in the right upper limb and dystonic posturing in the left upper limb. She became nonresponsive and agitated, displaying hyperkinetic complex motor automatisms, and then nose wiping with the right hand. EEG showed an evolving ictal rhythm in the right temporal region. She was unable to recall the events of the episode. This seizure is classified as a focal impaired consciousness seizure, with the following semiology: epigastric aura + fear ➔ automatisms in the right upper limb ➔ dystonic posturing in the left upper limb ➔ nonresponsive + impaired awareness ➔ hyperkinetic complex automatisms ➔ postictal nose wiping with the right hand.
- A 32‐year‐old right‐handed man has been experiencing brief seizures since the age of 11, primarily during sleep. EEG findings were unremarkable. At seizure onset, he reports an abnormal sensation in the right hand, described as a stretching feeling. During video‐EEG monitoring, he exhibited dystonic posturing of the right upper limb, along with hyperkinetic movements involving the other limbs and trunk. He was able to repeat test words during the seizure but could not recall them afterward and was confused postictally. Due to preserved verbal responsiveness, consciousness is considered preserved. Neuroimaging revealed a focal cortical dysplasia in the left middle frontal gyrus (Supplementary Material S10). This seizure is classified as a focal preserved consciousness seizure, with the following semiology: sensory aura in the right upper limb ➔ dystonic posturing in the right upper limb ➔ hyperkinetic movements in the left upper limb, bilaterally in the lower limbs, and in the trunk + preserved responsiveness ➔ postictal confusion ➔ postictal amnesia.
- A 25‐year‐old right‐handed woman has been experiencing seizures since the age of 13. At the onset of her typical seizures, she perceives bright flashes and moving spots in the left visual field, described as elementary visual phenomena moving horizontally. According to her family, she then becomes unresponsive. Video‐EEG monitoring captured a habitual seizure, beginning with the visual aura, followed by unresponsiveness, spitting, and chewing automatisms (Supplementary Material S11). Postictally, she experiences fatigue, headache, and vomiting. She is able to recall only the aura, but not the subsequent events. EEG showed ictal activity in the right temporal region, emerging after the clinical onset. MRI revealed a dysembryoplastic neuroepithelial tumor (DNET) in the right occipito‐parietal region. This seizure is classified as a focal impaired consciousness seizure, with the following semiology: elementary visual aura in the left hemifield ➔ unresponsiveness + spitting and oroalimentary automatisms ➔ postictal fatigue, headache, and vomiting.
- An 8‐month‐old girl presented with repetitive, brief contractions of axial muscles occurring in clusters upon awakening. EEG showed multifocal epileptiform discharges. One year later, seizures reappeared with two distinct seizure types: 1) Bilateral gestural automatisms with unresponsiveness, and 2) Repetitive contractions with left‐sided hemibody predominance (Supplementary Material S12). EEG during these events was lateralized to the right frontal lobe. MRI, histological analysis, and detection of somatic SLC35A2 variants confirmed a diagnosis of MOGHE (Mild Malformation of Cortical Development with Oligodendroglial Hyperplasia in Epilepsy) in the right frontal lobe. These seizures are classified as: 1) Focal impaired consciousness seizure, with the following semiology: bilateral gestural automatisms + impaired responsiveness, and 2) Focal seizures with epileptic spasms (focal spasms).
- A 5‐year‐old boy experienced episodes characterized by a diffuse shivering sensation, followed by tonic contraction of the left side and hyperkinetic automatisms on the right (Supplementary Material S13). Long‐term video‐EEG confirmed that awareness and responsiveness were preserved throughout the seizure. Neuroimaging showed a lesion in the right precentral area, and histopathology confirmed FCD type II. This seizure is classified as a focal preserved consciousness seizure, with the following semiology: somatosensory aura ➔ left focal tonic + right hyperkinetic behavior + preserved awareness and responsiveness.
- A 29‐year‐old man, who had a few febrile seizures in early childhood, remained seizure‐free until age 20, when unprovoked seizures began occurring almost daily. He occasionally experienced a sinking feeling and anxiety as an aura. During the seizures, he was unresponsive and had no recollection of the events afterward. MRI showed hippocampal sclerosis on the left side (Supplementary Material S14). This seizure is classified as a focal impaired consciousness seizure, with the following semiology: fear, anxiety ➔ oral and bimanual automatisms + nose wiping ➔ postictal confusion.
- A 6‐month‐old boy presented with new‐onset spells, mostly after awakening, that had been ongoing for the last two weeks. With these, he would have 1–2 seconds of extension of his left upper extremity with slight head or eye deviation to the left (Supplementary Material S15). These events would occur in brief clusters every 10–15 seconds for a total of 3–5 minutes, and he would have several clusters per day. He was born at 41 weeks gestation by emergency cesarean section due to failure to progress during his 2nd stage of labor. His Apgar scores were 4 and 8 at one and 5 minutes, and he did require some positive pressure ventilation at birth. He was noted to have a preference to turn his head to the right shortly after birth and was treated for torticollis. He had left hemiparesis on exam. His interictal EEG study was consistent with hypsarrhythmia: high‐amplitude delta slowing with very frequent multifocal epileptiform discharges, which were maximal in the right posterior head region. His ictal EEG recording showed sharp waves that were maximal in the right posterior head region, followed by brief low‐amplitude fast activity lasting 1–3 seconds with his clinical events. This seizure is classified as a focal seizure with infantile spasm (briefly: focal spasm). While epileptic spasms can be generalized, focal, or unknown whether generalized or focal, the most critical aspect is recognizing this unique seizure type and the initiation of spasms‐specific therapy, as delay can result in poor neurodevelopmental outcomes long term. In focal epileptic spasms, a structural etiology should be sought, and early surgical treatment considered if spasms‐specific therapy fails (as it did in this case).
- An 18‐year‐old woman with moderate intellectual disability had onset of epilepsy at age three years, and her seizures have been drug‐resistant. When she was younger, she would have episodes characterized by abrupt falling with loss of tone lasting 5–10 seconds. These have resolved, but more recently she has had increasing episodes of abrupt whole‐body stiffening with upward eye rolling lasting 10–15 seconds. These are not followed by clonic activity. The interictal EEG shows diffuse high amplitude slowing with 2–2.5 Hz slow spike waves during wakefulness (Supplementary Material S16). With her seizure, there is a high‐amplitude generalized spike–wave complex that is followed by diffuse low‐amplitude fast activity/generalized electrodecrement, which correlates with whole‐body stiffening (Supplementary Material S16). MRI shows a diffuse subcortical band heterotopia (Supplementary Material S16), and she was found to have a pathogenic variant in DCX. Because her seizure begins abruptly with generalized body stiffening and upward eye rolling, and her EEG clearly shows a bilateral‐synchronous/generalized onset. Although her eyes can deviate slightly to the left, this is still considered a generalized seizure. This seizure is classified as a generalized tonic seizure.
- A 15‐year‐old right‐handed boy with normal development has experienced jerks in the upper limbs, particularly in the morning, since the age of 12. He now presents with his first generalized convulsion, which was preceded by myoclonic jerks. His mother has a history of epilepsy. EEG revealed bilateral‐synchronous 3 Hz spike‐and‐wave complexes, which were exacerbated by hyperventilation and intermittent photic stimulation (Supplementary Material S17). MRI findings were normal. These seizures are classified as: (1) generalized myoclonic seizures, and (2) generalized myoclonic‐tonic–clonic seizures. The clinical and electrographic features are suggestive of juvenile myoclonic epilepsy (JME).
- A 22‐year‐old right‐handed woman with a known vascular malformation in the right temporal lobe underwent surgical treatment. She experienced her first seizure at age 14. During her seizures, she reported an indescribable sensation, followed by chewing movements and gestural automatisms involving the right hand, along with forced closure of the left fist. She was unable to respond during the episode. Interictal EEG demonstrated epileptiform discharges in the right temporal region (Supplementary Material S18). This seizure is classified as a focal impaired consciousness seizure, with the following semiology: indescribable aura ➔ oroalimentary automatisms + gestural automatisms of the right hand + dystonic posturing of the left hand + impaired responsiveness.
- A 16‐year‐old girl experienced a brief episode of staring and unresponsiveness lasting approximately 15 seconds, immediately followed by a bilateral tonic–clonic seizure lasting about one minute. She was drowsy in the postictal period. Her family reported occasional prior episodes of staring. This seizure is classified as an absence‐to‐tonic–clonic seizure.
- A 12‐year‐old boy presents with his first seizure, which occurred at school. Closed‐circuit television footage shows that he initially rotates his body to the right, followed by a forced head version to the right and eye deviation to the left. He then falls to the ground, exhibiting bilateral stiffening of all limbs and clonic jerks that gradually decrease in frequency toward the end of the episode (Supplementary Material S19). This seizure is classified as a focal‐to‐bilateral tonic–clonic seizure, with the following semiology: gyratory movement to the right → versive movement to the right + eye deviation to the left → bilateral tonic–clonic.
- A 35‐year‐old man presents with frequent episodes of sudden swearing. During long‐term video‐EEG monitoring, he was observed to swear abruptly while fumbling with the blanket using his left hand. He was unresponsive during the episode and had no recollection afterward. The EEG showed an evolving ictal rhythm in the left temporal region (Supplementary Material S20). This seizure is classified as a focal impaired consciousness seizure, with the following semiology: verbal automatism → gestural automatism of the left hand + impaired responsiveness and impaired awareness.
- A 28‐year‐old right‐handed man has presented with recurrent extension spasms since age 3 years ago. These seizures changed at age 12, beginning with a feeling of depersonalization that he describes as “being out of his mind,” followed by chest tightness and facial flushing according to witnesses. He was unable to respond verbally to calls, continuing with hyperkinetic movements (punching and kicking) and screaming for less than a minute. At the end, he reported recalling what happened. These seizures occur daily, both during wakefulness and sleep. The EEG revealed interictal epileptiform discharges in the left temporal region, and the recorded seizure showed an evolving ictal rhythm that began in the same region (Supplementary Material S21). MRI showed multiple cortical tubers, four of them located in the left temporal lobe, but also in the left frontal lobe (medial frontal gyrus), as well as the right cingulate region, among other regions. The patient meets major criteria for tuberous sclerosis: facial angiofibromas, shagreen patch, more than three hypomelanotic macules, subependymal nodules, and multiple cortical tubers (Supplementary Material S21). In this case, the patient's unresponsiveness was due to ictal aphasia and the ongoing hyperkinetic movement, not impaired consciousness, as evidenced by his ability to respond to nonverbal cues and his intact memory. This seizure is classified as a focal preserved consciousness seizure, with the following semiology: depersonalization aura ➔ flushing ➔ hyperkinetic complex automatisms + aphasia ➔ postictal nose wiping with the left hand.
- A 19‐year‐old woman developed seizures triggered by reading with a warning of words floating off the page and moving toward the right side of her body, followed by clonic jerking of the right side of her mouth with head and eye deviation to the right, repetitive blinking, and right arm extension prior to a bilateral tonic–clonic seizure with terminal left arm jerks (Supplementary Material S22). The seizure is classified as focal‐to‐bilateral tonic–clonic seizures with the following semiology: visual aura ➔ clonic jerks of the right side of the mouth + eye and head turning to the right ➔ impaired responsiveness ➔ bilateral tonic–clonic jerking with postictal confusion. Although formally not part of the seizure classification, it is important to add to the description that seizures are triggered by reading.
- A 38‐year‐old right‐handed man had a history of meningoencephalitis at one year of age. A few months later, he began experiencing brief episodes starting with a sensation of fear, which he described as “something paralyzed me.” He was unable to recall what happened afterward. Witnesses reported that during these episodes he became unresponsive, followed by forced head deviation to the left, and subsequently a bilateral convulsive seizure. Despite treatment with antiseizure medications, he continued to have approximately one seizure per month. During long‐term video‐EEG monitoring, interictal epileptiform discharges were recorded in the right temporal region, and the captured seizure showed an evolving ictal rhythm beginning in the same area (Supplementary Material S23). The MRI showed right hippocampal sclerosis. This seizure is classified as a focal‐to‐bilateral tonic–clonic seizure, with the following semiology: ictal fear ➔ bilateral gestural automatisms ➔ impaired responsiveness ➔ bipedal hyperkinetic automatisms ➔ mimic automatism ➔ versive head turning to the left ➔ asymmetrical tonic posture in the left upper limb ➔ clonic jerks in the left upper limb and left hemiface ➔ bilateral tonic phase ➔ figure‐of‐four posture with extension of the right elbow ➔ bilateral tonic–clonic ➔ final clonic jerks on the right ➔ postictal unresponsiveness.
- A 41‐year‐old right‐handed man presents with brief episodes of unusual behavior. According to his wife, he does not respond when spoken to during these episodes. During a routine EEG recording, the patient experienced a seizure in which he failed to follow verbal commands, but he responded to visual cues and gestural commands. He also developed oroalimentary automatisms (lip smacking). After the seizure, he was able to fully recall the events that occurred during the episode. The EEG revealed interictal epileptiform discharges in the left temporal region, and the recorded seizure showed an evolving ictal rhythm starting in the same region. Neuroimaging identified a tumor in the left temporal lobe. In this case, the patient's unresponsiveness was due to ictal aphasia, not impaired consciousness, as evidenced by his ability to respond to nonverbal cues and his intact recall. The seizure is therefore classified as a focal preserved consciousness seizure, with the following semiology: ictal aphasia ➔ oroalimentary automatisms ➔ postictal aphasia.
- An 18‐year‐old right‐handed woman is undergoing presurgical evaluation for drug‐resistant epilepsy. MRI revealed left hippocampal sclerosis. During long‐term video‐EEG monitoring, the patient reported an ascending epigastric sensation, followed by automatisms in the left hand and oroalimentary automatisms. She was able to follow verbal commands and repeat test words during the seizure. However, during the postictal interview, she was unable to recall what had occurred during the episode. In this case, the lack of recall is attributed to postictal amnesia, not impaired consciousness, as the patient was fully responsive throughout the seizure. The seizure is therefore classified as a focal preserved consciousness seizure, with the following semiology: epigastric aura ➔ left‐hand automatism + oroalimentary automatism ➔ postictal amnesia.
- A 22‐year‐old right‐handed man is undergoing presurgical evaluation for drug‐resistant epilepsy. MRI was unrevealing. During long‐term video‐EEG monitoring, the patient reported a tingling sensation on the left side of the body. Behavioral testing was initiated by the nurse after the patient signaled the onset of the aura. He was able to follow the first command to raise his right arm but did not follow the next command to raise his left arm. The patient then indicated that he was unable to move his left arm and leg. Approximately five minutes after the seizure, he had full motor recovery, with the ability to move all limbs. He retained full recollection of the entire episode. Although he was unable to follow one of the commands during the seizure, this was due to ictal motor impairment (ictal paresis) rather than impaired consciousness. The seizure is therefore classified as a focal preserved consciousness seizure, with the following semiology: paresthesia on the left side of the body ➔ left‐sided ictal paresis.
- A 24‐year‐old right‐handed man with seizures since the age of 3 years has been subsequently diagnosed with a grade 3 astrocytoma in the left temporo‐parieto‐occipital regions abutting the midbrain. He underwent subsequent resection and then chemotherapy. He was seizure‐free until seizure recurrence at age 7. The patient is now referred for presurgical evaluation. He describes seizures with a preceding sensation of a “feeling in his eyes as if dragged to the right side” (in video right gaze deviation), on some occasions followed by right head version, right arm sensation, and inability to speak. He may perceive a right arm flexion like “it is locked in”. The overall duration is 10–20 seconds prior to self‐resolution. He recalls the seizure. Current seizure frequency is 3 per day. He has right homonymous hemianopsia. EEG points to the left posterior temporo‐occipital region. MRI shows the remote resection with removal of large portions of the left parietal lobe and anterior/medial temporal lobe. The seizure is classified as focal preserved consciousness seizure with the following semiology: eye and head deviation to the right ➔ somatosensory non‐painful sensation in the right upper limb ➔ expressive aphasia + dystonia in the right upper limb.
- A 24‐year‐old right‐handed woman has had seizure onset at age 23. Her seizures are characterized by eye blinking, drooling, and pouting. Bystanders report that she would be only partially responsive during these episodes. The patient vaguely remembers the seizures. After the seizure, the patient rapidly recuperates. Seizure frequency is 5–6 seizures per month, usually occurring in clusters. Interictal EEG is normal, but the ictal EEG points to the right fronto‐central region. The MRI reveals a lesion suspicious of DNET in the right superior mid insula. The seizure is classified as a focal impaired consciousness seizure with the following semiology: eye blinking ➔ hypersalivation + “chapeau‐de‐gendarme” + partial responsiveness ➔ postictal rapid recuperation with partial awareness of the seizure.
- A 22‐year‐old man developed seizures at 17 years of age. Weekly seizures consist of two semiologies, one beginning with an aura of déjà vu and another as a heat sensation appearing flushed in the face, with retained responsiveness and awareness (Supplementary Material S24). Focal seizures were described as typically occurring in prolonged clusters, intermittently evolving to left head deviation prior to bilateral convulsions and status epilepticus. Interictal and ictal EEG showed epileptiform activity in the right centro‐temporal region, and Stereo‐EEG showed the seizure‐onset zone in the right hippocampus. These seizures are classified as 1) focal preserved consciousness seizure, with the following semiology: aura (flushed, “strange,” feeling or déjà vu) + preserved responsiveness and awareness, and 2) focal‐to‐bilateral tonic–clonic seizure, with semiology starting as type #1 and then evolving to head deviation to the left and bilateral tonic–clonic phase.
- A 21‐month‐old girl experiences daily episodes of behavioral arrest. During video‐EEG monitoring, she was observed to suddenly stop playing, becoming inactive with a fixed gaze and no blinking for 46 seconds (Supplementary Material S25). Throughout the episode, she repeatedly manipulated her pacifier with her left hand. No tonic or clonic movements were noted, and she returned to baseline without postictal confusion. Interictal EEG showed sharp waves in the left temporoparietal region, and the underlying etiology was identified as a left temporal tumor. This seizure is classified as a focal seizure, with the following semiology: motor and behavioral arrest ➔ automatisms in the left hand.
- A previously healthy 17‐year‐old girl experienced sudden bilateral arm jerks upon awakening, followed within seconds by tonic stiffening and bilateral convulsions lasting approximately 90 seconds (Supplementary Material S26). She was postictally confused for about 10 minutes. This seizure is classified as a myoclonic‐tonic–clonic seizure, which is typical of JME.

## 6 CONCLUSION

The updated ILAE seizure classification offers a flexible, clinically meaningful framework to improve communication, diagnosis, and management of patients across the epilepsy care continuum. By distinguishing between focal, generalized, unknown origin, and unclassified seizure classes, and incorporating both basic and expanded versions of the classification, clinicians can apply the system at varying levels of detail based on available information and resources. Key concepts, such as the role of consciousness, the use of semiological descriptors, and the identification of well‐defined and documented seizure types, are critical for accurate classification and guide appropriate therapeutic choices.

The inclusion of illustrative case vignettes and supplementary materials demonstrates the practical application of the classification in diverse clinical contexts. Whether used in primary care, epilepsy centers, or presurgical evaluations, this system facilitates a shared language and consistent decision‐making pathway.

Ultimately, the updated seizure classification serves not only as a diagnostic tool but also as a foundation for personalized, evidence‐based epilepsy care. Its structured, yet adaptable, design ensures it remains relevant and useful across the spectrum of clinical practice and research.

## Supporting information


Data S1.

Data S2.

Data S3.

Data S4.

Data S5.

Data S6.

Data S7.

Data S8.

Data S9.

Data S10.

Data S11.

Data S12.

Data S13.

Data S14.

Data S15.

Data S16.

Data S17.

Data S18.

Data S19.

Data S20.

Data S21.

Data S22.

Data S23.

Data S24.

Data S25.

Data S26.

## Data Availability

Data sharing not applicable to this article as no datasets were generated or analysed during the current study.

## References

[1] Fisher RS , Cross JH , French JA , Higurashi N , Hirsch E , Jansen FE , et al. Operational classification of seizure types by the international league against epilepsy: position paper of the ILAE Commission for Classification and Terminology. Epilepsia. 2017;58:522–530. 10.1111/epi.13670 28276060

[2] Fisher RS , Cross JH , D'Souza C , French JA , Haut SR , Higurashi N , et al. Instruction manual for the ILAE 2017 operational classification of seizure types. Epilepsia. 2017;58:531–542. 10.1111/epi.13671 28276064

[3] Beniczky S , Trinka E , Wirrell E , Abdulla F , Al Baradie R , Alonso Vanegas M , et al. Updated classification of epileptic seizures: position paper of the international league against epilepsy. Epilepsia. 2025;66(6):1804–1823. 10.1111/epi.18338 40264351 PMC12169392

[4] Beniczky S , Trinka E , Wirrell E , Specchio N , Cendes F , Helen Cross J . Updating the ILAE seizure classification. Epilepsia. 2025;66:1824–1826. 10.1111/epi.18399 40264360

[5] Trinka E , Cock H , Hesdorffer D , Rossetti AO , Scheffer IE , Shinnar S , et al. A definition and classification of status epilepticus‐‐report of the ILAE task force on classification of status epilepticus. Epilepsia. 2015;56:1515–1523. 10.1111/epi.13121 26336950

[6] Pressler RM , Cilio MR , Mizrahi EM , Moshé SL , Nunes ML , Plouin P , et al. The ILAE classification of seizures and the epilepsies: modification for seizures in the neonate. Position paper by the ILAE task force on neonatal seizures. Epilepsia. 2021;62:615–628. 10.1111/epi.16815 33522601

[7] Berg AT , Berkovic SF , Brodie MJ , Buchhalter J , Cross JH , van Emde Boas W , et al. Revised terminology and concepts for organization of seizures and epilepsies: report of the ILAE commission on classification and terminology, 2005‐2009. Epilepsia. 2010;51:676–685. 10.1111/j.1528-1167.2010.02522.x 20196795

[8] Koutroumanidis M , Arzimanoglou A , Caraballo R , Goyal S , Kaminska A , Laoprasert P , et al. The role of EEG in the diagnosis and classification of the epilepsy syndromes: a tool for clinical practice by the ILAE neurophysiology task force (part 1). Epileptic Disord. 2017;19:233–298. 10.1684/epd.2017.0935 28984246

[9] Koutroumanidis M , Arzimanoglou A , Caraballo R , Goyal S , Kaminska A , Laoprasert P , et al. The role of EEG in the diagnosis and classification of the epilepsy syndromes: a tool for clinical practice by the ILAE neurophysiology task force (part 2). Epileptic Disord. 2017;19:385–437. 10.1684/epd.2017.0952 29350182

[10] Asadi‐Pooya AA , Beniczky S , Rubboli G , Sperling MR , Rampp S , Perucca E . A pragmatic algorithm to select appropriate antiseizure medications in patients with epilepsy. Epilepsia. 2020;61:1668–1677. 10.1111/epi.16610 32697354

[11] Brigo F , Tavernelli V , Nardone R , Trinka E . De novo late‐onset absence status epilepticus or late‐onset idiopathic generalized epilepsy? A case report and systematic review of the literature. Epileptic Disord. 2018;20(2):123–131. 10.1684/epd.2018.0961 29620008

[12] Reichsoellner J , Larch J , Unterberger I , Dobesberger J , Kuchukhidze G , Luef G , et al. Idiopathic generalised epilepsy of late onset: a separate nosological entity? J Neurol Neurosurg Psychiatry. 2010;81:1218–1222. 10.1136/jnnp.2009.176651 20802210

[13] Trinka E . Absences in adult seizure disorders. Acta Neurol Scand. 2005;182:12–18. 10.1111/j.1600-0404.2005.00522.x 16359428

[14] Vorderwülbecke BJ , Wandschneider B , Weber Y , Holtkamp M . Genetic generalized epilepsies in adults ‐ challenging assumptions and dogmas. Nat Rev Neurol. 2022;18:71–83. 10.1038/s41582-021-00583-9 34837042

[15] Vlachou M , Ryvlin P , Armand Larsen S , Beniczky S . Focal electroclinical features in generalized tonic‐clonic seizures: decision flowchart for a diagnostic challenge. Epilepsia. 2024;65:725–738. 10.1111/epi.17895 38279904

[16] Leutmezer F , Lurger S , Baumgartner C . Focal features in patients with idiopathic generalized epilepsy. Epilepsy Res. 2002;50(3):293–300. 10.1016/s0920-1211(02)00084-0 12200220

[17] Beniczky S , Tatum WO , Blumenfeld H , Stefan H , Mani J , Maillard L , et al. Seizure semiology: ILAE glossary of terms and their significance. Epileptic Disord. 2022;24:447–495. 10.1684/epd.2022.1430 35770761

[18] Sveinsson O , Andersson T , Mattsson P , Carlsson S , Tomson T . Clinical risk factors in SUDEP: a nationwide population‐based case‐control study. Neurology. 2020;94:e419–e429. 31831600 10.1212/WNL.0000000000008741 PMC7079690

[19] Harden C , Tomson T , Gloss D , Buchhalter J , Cross JH , Donner E , et al. Practice guideline summary: sudden unexpected death in epilepsy incidence rates and risk factors: report of the guideline development, dissemination, and implementation Subcommittee of the American Academy of neurology and the American Epilepsy Society. Neurology. 2017;88:1674–1680. 28438841 10.1212/WNL.0000000000003685

[20] Contreras Ramirez V , Vaddiparti A , Blumenfeld H . Testing awareness in focal seizures: clinical practice and interpretation of current guidelines. Ann Clin Transl Neurol. 2022;9:762–765. 10.1002/acn3.51552 35485201 PMC9082375

[21] Beniczky S , Neufeld M , Diehl B , Dobesberger J , Trinka E , Mameniskiene R , et al. Testing patients during seizures: a European consensus procedure developed by a joint taskforce of the ILAE ‐ commission on European affairs and the European epilepsy monitoring unit association. Epilepsia. 2016;57:1363–1368. 10.1111/epi.13472 27440172

[22] Contreras Ramirez V , Patedakis Litvinov B , Gunawardane NA , Zhao CW , Yotter C , Quraishi IH , et al. Evaluating consciousness and awareness during focal seizures: responsiveness testing versus recall testing. Epileptic Disord. 2022;24:899–905. 10.1684/epd.2022.1472 35904040 PMC10042123

[23] Blumenfeld H , Meador K , Jackson GD . Commentary: the return of consciousness to epilepsy seizure classification. Epilepsia. 2015;56:345–347. 10.1111/epi.12922 25740196 PMC4688007

[24] Blumenfeld H , Meador KJ . Consciousness as a useful concept in epilepsy classification. Epilepsia. 2014;55:1145–1150. 10.1111/epi.12588 24981294 PMC4149314

[25] Gastaut H . Clinical and electroencephalographic classification of epileptic seizures. Epilepsia. 1970;11:102. 5268244 10.1111/j.1528-1157.1970.tb03871.x

[26] Commission on Classification and Terminology of the International League Against Epilepsy . Proposal for revised clinical and electroencephalographic classification of epileptic seizures. From the commission on classification and terminology of the international league against epilepsy. Epilepsia. 1981;22:489–501. 10.1111/j.1528-1157.1981.tb06159.x 6790275

[27] Guo JN , Kim R , Chen Y , Negishi M , Jhun S , Weiss S , et al. Impaired consciousness in patients with absence seizures investigated by functional MRI, EEG, and behavioural measures: a cross‐sectional study. Lancet Neurol. 2016;15:1336–1345. 27839650 10.1016/S1474-4422(16)30295-2 PMC5504428

[28] Bell WL , Walczak TS , Shin C , Radtke RA . Painful generalised clonic and tonic‐clonic seizures with retained consciousness. J Neurol Neurosurg Psychiatry. 1997;63(6):792–795. 9416819 10.1136/jnnp.63.6.792 PMC2169852

[29] Blumenfeld H . Consciousness and epilepsy: why are patients with absence seizures absent? Prog Brain Res. 2005;150:271–286. 16186030 10.1016/S0079-6123(05)50020-7 PMC3153469

[30] Steriade C , Sperling MR , DiVentura B , Lozano M , Shellhaas RA , Kessler SK , et al. Proposal for an updated seizure classification framework in clinical trials. Epilepsia. 2022;63:565–572. 10.1111/epi.17120 34997581 PMC9302660

[31] Lüders H , Acharya J , Baumgartner C , Benbadis S , Bleasel A , Burgess R , et al. Semiological seizure classification. Epilepsia. 1998;39:1006–1013. 9738682 10.1111/j.1528-1157.1998.tb01452.x

[32] Lüders H , Akamatsu N , Amina S , Baumgartner C , Benbadis S , Bermeo‐Ovalle A , et al. Critique of the 2017 epileptic seizure and epilepsy classifications. Epilepsia. 2019;60:1032–1039. 10.1111/epi.14699 30924146

[33] Zuberi SM , Wirrell E , Yozawitz E , Wilmshurst JM , Specchio N , Riney K , et al. ILAE classification and definition of epilepsy syndromes with onset in neonates and infants: position statement by the ILAE task force on nosology and definitions. Epilepsia. 2022;63:1349–1397. 10.1111/epi.17239 35503712

[34] Tavella‐Burka S , Hartnett P , Quigg M . Seizure classifications across the years: a translation table. Epilepsia. 2025. 10.1111/epi.18608 40956031

