# Supplementary material for: A practical guide to the updated seizure classification 2025
Source: Epileptic Disord. 2025 Oct 13;27(6):1087–104. doi: 10.1002/epd2.70110 (PMC12747708; doi:10.1002/epd2.70110)
Supplement: Supplementary file 1 — Data S1. [file EPD2-27-1087-s002.pptx]

## Slide 1
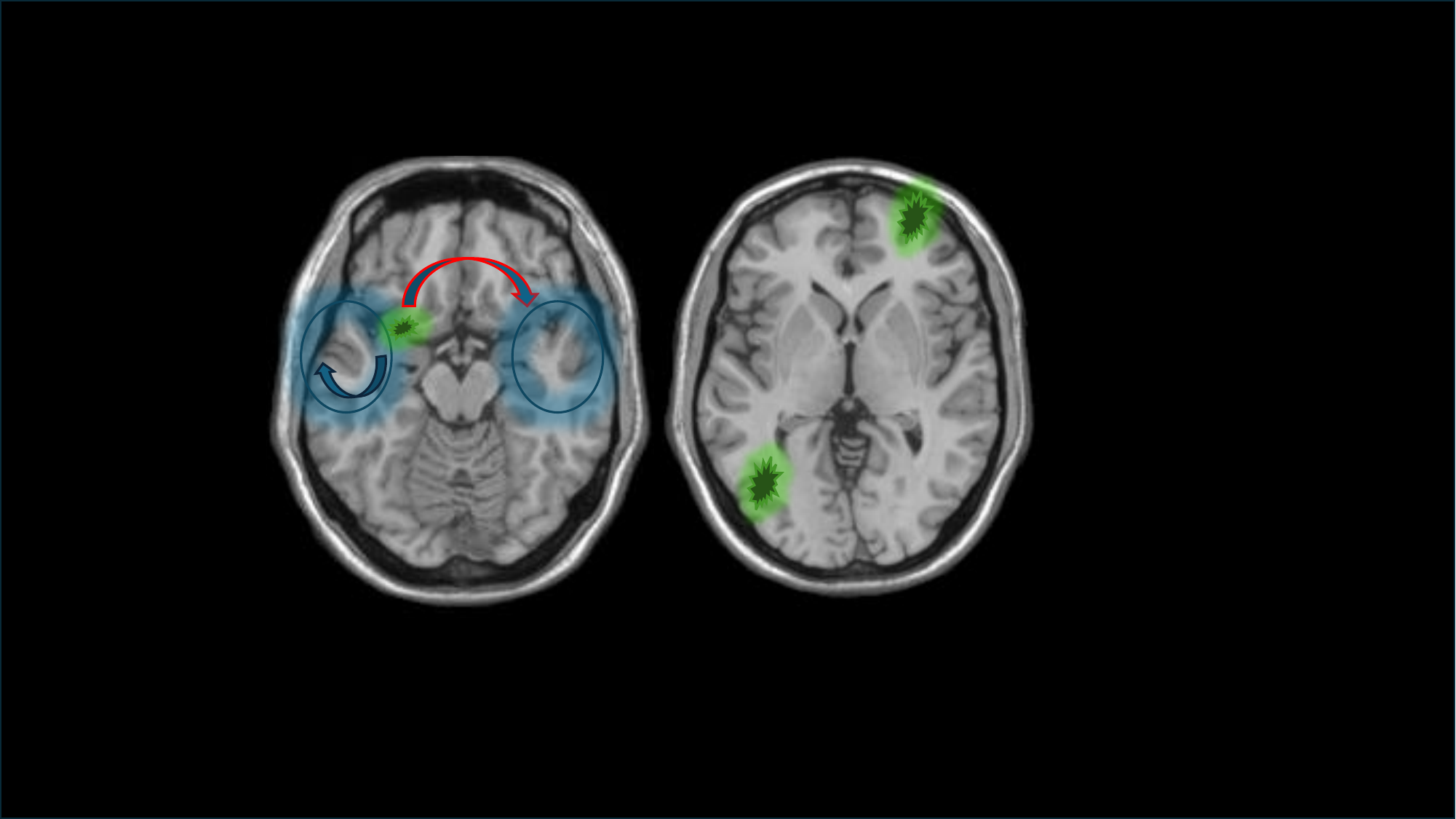

## Slide 2
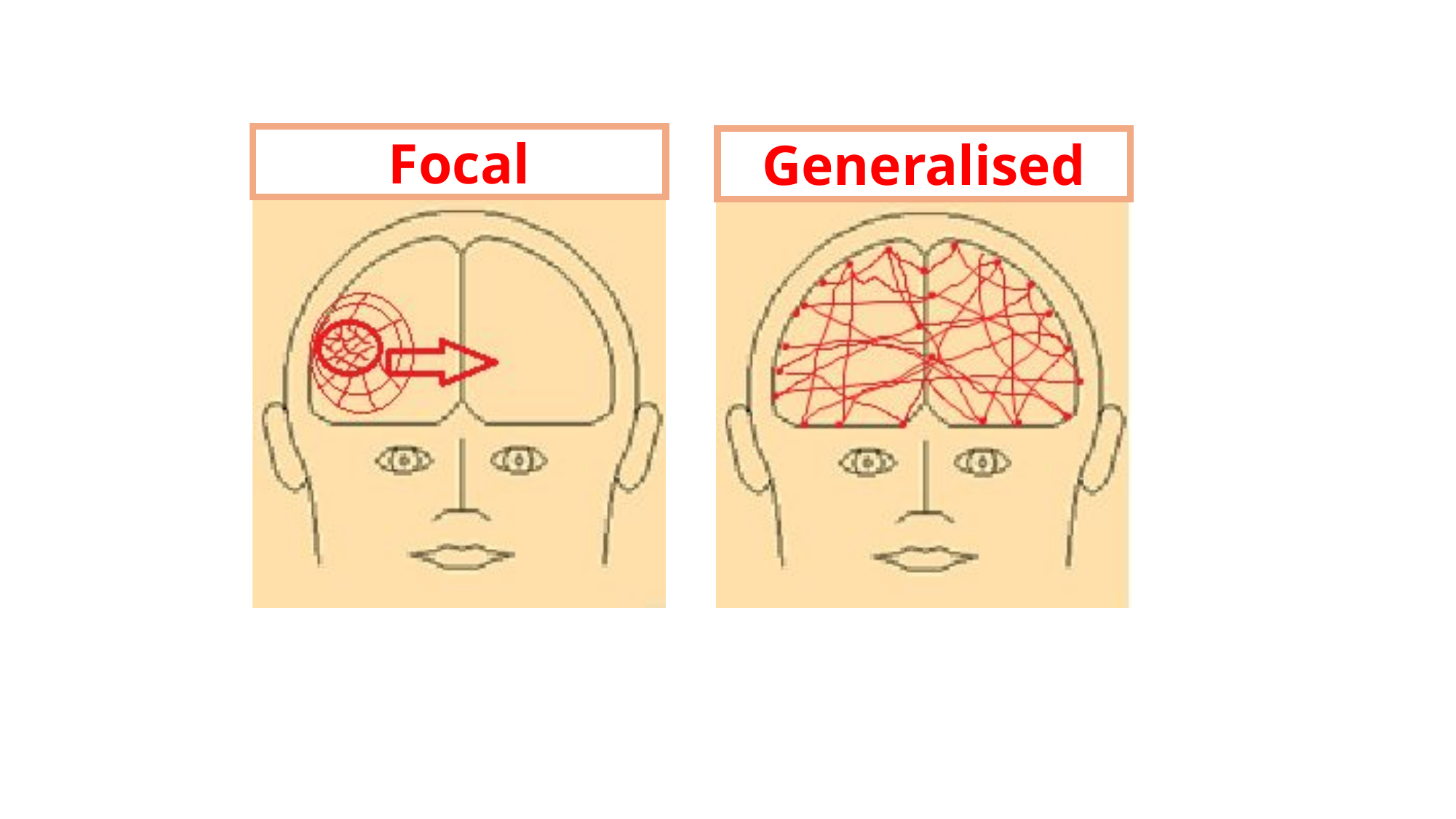

Focal
Generalised

## Slide 3
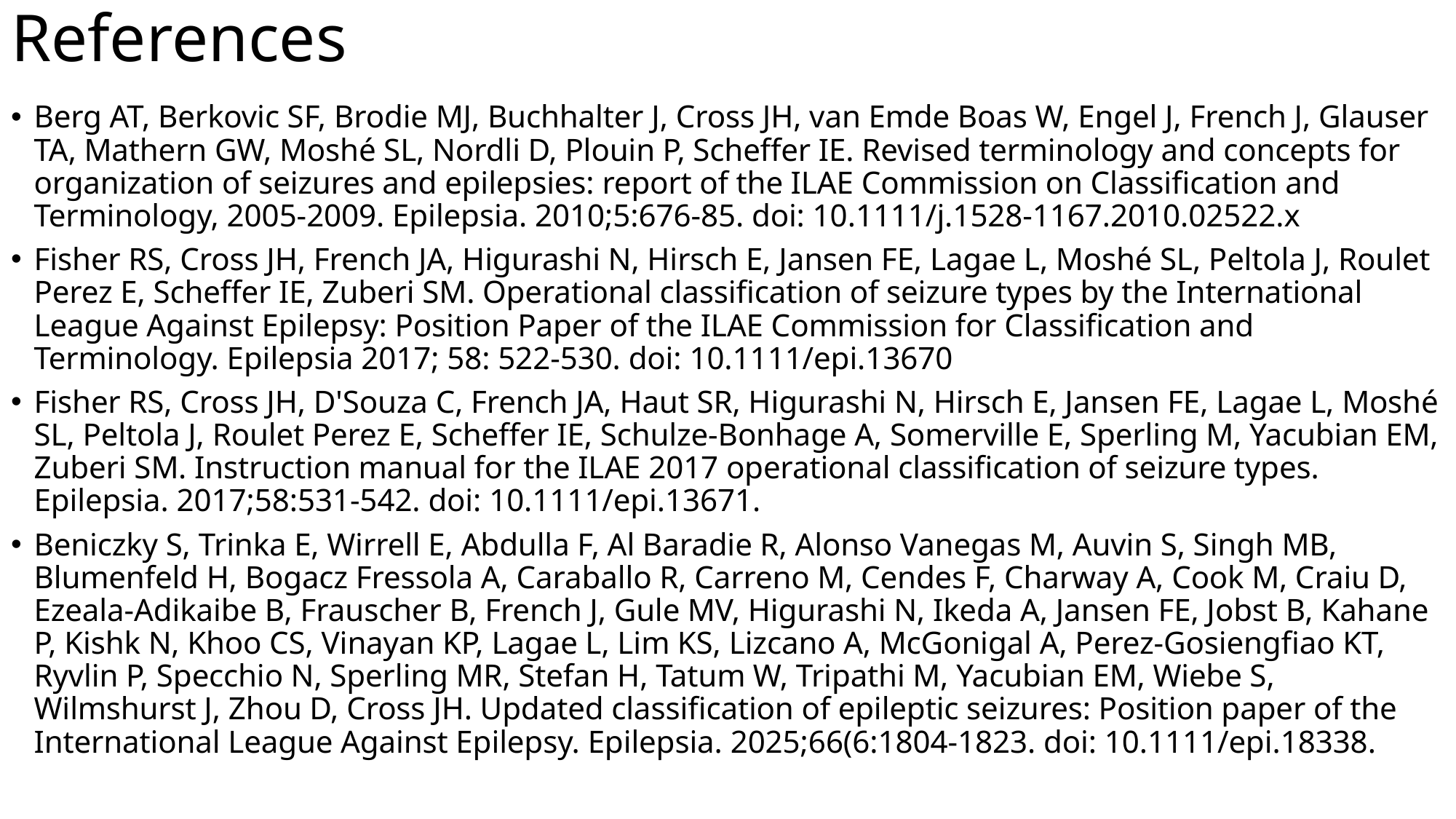

# References
Berg AT, Berkovic SF, Brodie MJ, Buchhalter J, Cross JH, van Emde Boas W, Engel J, French J, Glauser TA, Mathern GW, Moshé SL, Nordli D, Plouin P, Scheffer IE. Revised terminology and concepts for organization of seizures and epilepsies: report of the ILAE Commission on Classification and Terminology, 2005-2009. Epilepsia. 2010;5:676-85. doi: 10.1111/j.1528-1167.2010.02522.x
Fisher RS, Cross JH, French JA, Higurashi N, Hirsch E, Jansen FE, Lagae L, Moshé SL, Peltola J, Roulet Perez E, Scheffer IE, Zuberi SM. Operational classification of seizure types by the International League Against Epilepsy: Position Paper of the ILAE Commission for Classification and Terminology. Epilepsia 2017; 58: 522-530. doi: 10.1111/epi.13670
Fisher RS, Cross JH, D'Souza C, French JA, Haut SR, Higurashi N, Hirsch E, Jansen FE, Lagae L, Moshé SL, Peltola J, Roulet Perez E, Scheffer IE, Schulze-Bonhage A, Somerville E, Sperling M, Yacubian EM, Zuberi SM. Instruction manual for the ILAE 2017 operational classification of seizure types. Epilepsia. 2017;58:531-542. doi: 10.1111/epi.13671.
Beniczky S, Trinka E, Wirrell E, Abdulla F, Al Baradie R, Alonso Vanegas M, Auvin S, Singh MB, Blumenfeld H, Bogacz Fressola A, Caraballo R, Carreno M, Cendes F, Charway A, Cook M, Craiu D, Ezeala-Adikaibe B, Frauscher B, French J, Gule MV, Higurashi N, Ikeda A, Jansen FE, Jobst B, Kahane P, Kishk N, Khoo CS, Vinayan KP, Lagae L, Lim KS, Lizcano A, McGonigal A, Perez-Gosiengfiao KT, Ryvlin P, Specchio N, Sperling MR, Stefan H, Tatum W, Tripathi M, Yacubian EM, Wiebe S, Wilmshurst J, Zhou D, Cross JH. Updated classification of epileptic seizures: Position paper of the International League Against Epilepsy. Epilepsia. 2025;66(6:1804-1823. doi: 10.1111/epi.18338.
