# Supplementary material for: A practical guide to the updated seizure classification 2025
Source: Epileptic Disord. 2025 Oct 13;27(6):1087–104. doi: 10.1002/epd2.70110 (PMC12747708; doi:10.1002/epd2.70110)
Supplement: Supplementary file 2 — Data S2. [file EPD2-27-1087-s013.docx]

***Supplementary Material 2***

**Evaluation of Consciousness During Seizures
Based on Responsiveness and Awareness**

In concordance with other neurological disorders, the updated seizure classification uses both responsiveness and awareness to classify impairment of consciousness during seizures. This can be evaluated based on patient history, ideally including family members or other observers, and when possible, using video recordings of seizures obtained at home or in the hospital.

Instead of asking about consciousness, which can be misunderstood, while taking the history, patients or observers should be asked if the patient is able to respond normally during seizures, and if they are able to recall events during the seizures (as an indication of awareness). If videos of seizures are available, the entire ictal period should be reviewed to evaluate responsiveness. At least five minutes or more of the postictal period should be reviewed, if possible, to determine if the patient recalls and describes events accurately that occurred during the seizure.

Combined information from the assessment of both awareness and responsiveness are used to determine consciousness (*Supplementary Table 1*). If both are impaired, then the seizure is classified as having impaired consciousness. If either one is definitely preserved, then the seizure is classified as having preserved consciousness. This can occur for example with impaired awareness (recall) but preserved responsiveness in cases of ictal amnesia with preserved consciousness. The converse situation with impaired responsiveness but preserved awareness (recall) can occur with ictal motor impairment or ictal aphasia without impaired consciousness.

***Supplementary Table 1:*** *Classification of consciousness in focal seizures when both assessment of awareness and responsiveness are available*

***Responsiveness Awareness Classification***

*Impaired Impaired Focal Impaired Consciousness (FIC) Seizure*

*Spared Spared Focal Preserved Consciousness (FPC) Seizure*

*Impaired Spared Focal Preserved Consciousness (FPC) Seizure*

*Spared Impaired Focal Preserved Consciousness (FPC) Seizure*

Often in practice, information is only available about one of the two – either responsiveness, or awareness but not both. In that case without further information the determination is based completely on whichever of the two is available (*Supplementary Table 2*).

***Table 2:*** *Classification of consciousness in focal seizures when only assessment of awareness or responsiveness is available, but not both*

***Responsiveness Awareness Classification***

*Impaired Not Available Focal Impaired Consciousness (FIC) Seizure*

*Not Available Impaired Focal Impaired Consciousness (FIC) Seizure*

*Spared Not Available Focal Preserved Consciousness (FPC) Seizure*

*Not Available Spared Focal Preserved Consciousness (FPC) Seizure*

Objective evaluation of responsiveness requires an external question, command or other stimulus during the seizure. In some seizures no stimulus is presented, but the patient has a fixed stare, behavioral arrest or automatisms, making it tempting to speculate that the patient was unresponsive. However, determination of unresponsiveness, with no external stimulus, remains speculative. Similarly, determination of awareness requires an external query or spontaneous report by the patient about events during the seizure that can be objectively verified.

In most cases, the evaluation of consciousness is straightforward, and it is possible to decide if consciousness is impaired or preserved. However, as described in further detail in the sections that follow, there are situations where the state of consciousness remains uncertain due to a combination of factors. In such uncertain cases, consciousness can be omitted from the classification, and the parent term used without further specification (for example: focal seizure). However, if there is clinical suspicion that consciousness may be impaired, for driving safety considerations, the seizure can provisionally be classified as having impaired consciousness until further confirmatory information is obtained.

**Responsiveness**

**Timing for evaluation of responsiveness:**

Responsiveness assessment is based on verbal or nonverbal responses during seizures. It is important to note that responsiveness may vary during the course of a seizure. If responsiveness is impaired at any time during the seizure, then it should be classified as impaired.

Assessment of responsiveness should be during the ictal, not preictal or postictal periods. However, if responsiveness was only assessed in the postictal period, then postictal impairment might suggest that there was impairment during the ictal period as well. On the other hand, *preserved* postictal responsiveness does *not* imply preserved function in the ictal period.

**Stimuli for evaluating responsiveness:**

Useful stimuli for evaluating responsiveness are those that would typically elicit a response under normal conditions. Valid stimuli are usually questions, commands or other salient verbal or non-verbal stimuli for which a response is expected in a normal individual. Examples of valid stimuli include calling the patient’s name, a loud sound in the room, waving hello/goodbye, reaching out to shake the patient's hand in greeting, or vigorously shaking the patient’s shoulder. However, touching the patient in a neutral or soothing way, or other neutral sounds or conversation in the room not directed at the patient should not be considered valid stimuli, because a normal individual might ignore these. If unsure or unclear, the rating of impaired consciousness should remain unknown.

**Responses:**

Preserved responses are meaningful, purposeful responses to stimuli, which are cortically-mediated. As in the assessment of patients in coma or vegetative state, simple orientation of head or eyes towards a salient visual, auditory or tactile stimulus is not considered as positive evidence for consciousness. Examples of impaired responses include no response; verbal responses that are slurred, incorrect, aphasic or incomprehensible (unless there is evidence that this is only due to impaired language/motor function without impaired consciousness); non-verbal responses that are performed incorrectly; or responses with significantly longer response latency than the patient’s baseline. If unsure or unclear whether the responses were normal and meaningful, the rating of impaired consciousness should remain unknown.

Automatic behaviors as part of the patient’s typical seizure do not count as valid preserved responses, e.g. manual or oral automatisms, verbal or non-verbal vocalizations, clonic or tonic movements, version, RINCH, etc. If unsure or unclear whether the behavior was meaningful or part of the seizure behavior, the rating of impaired consciousness should remain unknown.

Responses may be seen in some circumstances even if an external stimulus was not observed. For example, if the patient has a normal phone conversation even though the other speaker cannot be heard, or has normal spontaneous speech even if not spoken to, or carries out other spontaneous behavior that can be verified as meaningful and normal, e.g. writing (only if normal writing can be verified), or carrying out other meaningful verifiable tasks, these should be considered normal responses. However, if the patient’s actions cannot be verified, e.g. appearing to play a game on a phone, appearing to write (without verification), or moving the mouth where spoken words can’t be interpreted, these should not be considered normal responses. Again, if unsure or unclear whether the behavior was meaningful or not, the rating of impaired consciousness should remain unknown.

Some responses may be difficult to classify as normal or impaired consciousness, and should be investigated in future research studies. For example, if the patient responds slowly but otherwise normally and meaningfully, this might indicate some preserved consciousness. In addition, simple, cortically-mediated behaviors such as consistent visual tracking (which differs from the visual orienting response), grasping a ball placed on the dorsum of the hand, blink to visual threat, or withdrawal from a painful stimulus may be seen in some patients who otherwise appear to be unconscious. Such behaviors in chronic disorders of consciousness are considered as supportive evidence for the minimally conscious state, and with further investigation could be relevant for epilepsy assessment as well.

**Awareness**

**Timing for evaluation of awareness:**

Awareness assessment is based on report of events that occur during a seizure. Usually, this report occurs after the seizure is over and therefore requires memory. In some cases, the patient may be able to accurately describe events occurring while the seizure is ongoing. Like responsiveness, it is important to note that awareness may vary during the course of a seizure. If awareness is impaired for events that occurred at any time during the seizure, then the seizure should be classified as impaired.

Report of events that occur before seizure onset (preictal) or after the seizure end (postictal) should not be considered as evidence for ictal awareness. However, if patients are unaware of events that occurred in the postictal period, this might suggest that they were unaware in the ictal period as well.

**Events for awareness assessment:**

The best assessment of awareness is based on verbal report of externally verifiable events that occurred during the seizure. Examples of externally verifiable events that could be reported by the patient include words or phrases presented deliberately to the patient for testing purposes, details of conversations with others, entry of someone into the room, or other externally observable events.

Certain events are not reliable for assessment of awareness during seizures. Internal events cannot be observed externally or objectively verified, and therefore cannot provide definite evidence of awareness during seizures. Examples include sensations, thoughts or feelings during the seizure. It is also very important to distinguish between patient awareness of external events during the seizure, and patient awareness of the seizure itself. After the seizure has ended, if the patient reports that a seizure has occurred, this usually cannot be used as evidence that they were aware during the seizure for the following reasons: 1. Patients often experience postictal symptoms such as muscle aches, head pain, confusion, etc. that provide clues that a seizure has occurred; 2. Other people often will tell the patient or provide nonverbal clues that a seizure has occurred; 3. Patient description of their own seizure behavior, even if externally verifiable (e.g. right hand twitching) usually is not good evidence for awareness during the seizure, because such behaviors are typically stereotyped and the patient may be describing what they know about previous seizures, not the specific one that just occurred. On the other hand, if *during* the seizure the patient says that they are having a seizure or describes their seizure behavior while the seizure is ongoing, this can be taken as reliable evidence of awareness. If the patient pushes an event button during the seizure to report that they are having a seizure, this might be an indication of awareness at least at that specific time during the seizure. However, this should be interpreted cautiously because patients will often press the event button at seizure onset, and may then lose consciousness at later times. As with the evaluation of responsiveness, if the assessment of awareness is uncertain or unclear then the classification of consciousness can be left undetermined when appropriate.
