# Supplementary figures and images for: A practical guide to the updated seizure classification 2025
Source: Epileptic Disord. 2025 Oct 13;27(6):1087–104. doi: 10.1002/epd2.70110 (PMC12747708; doi:10.1002/epd2.70110)

## Slide 1
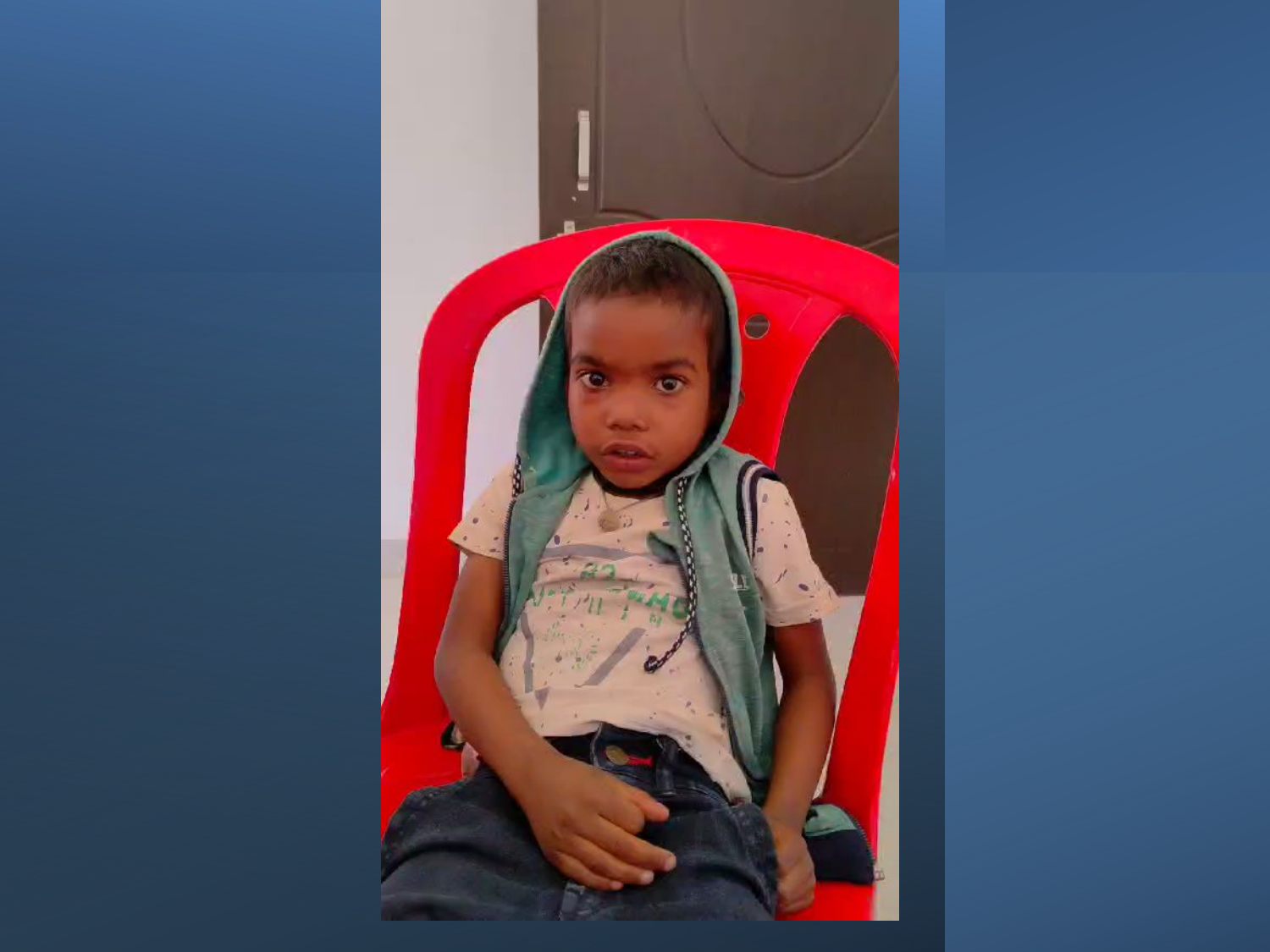

## Slide 2
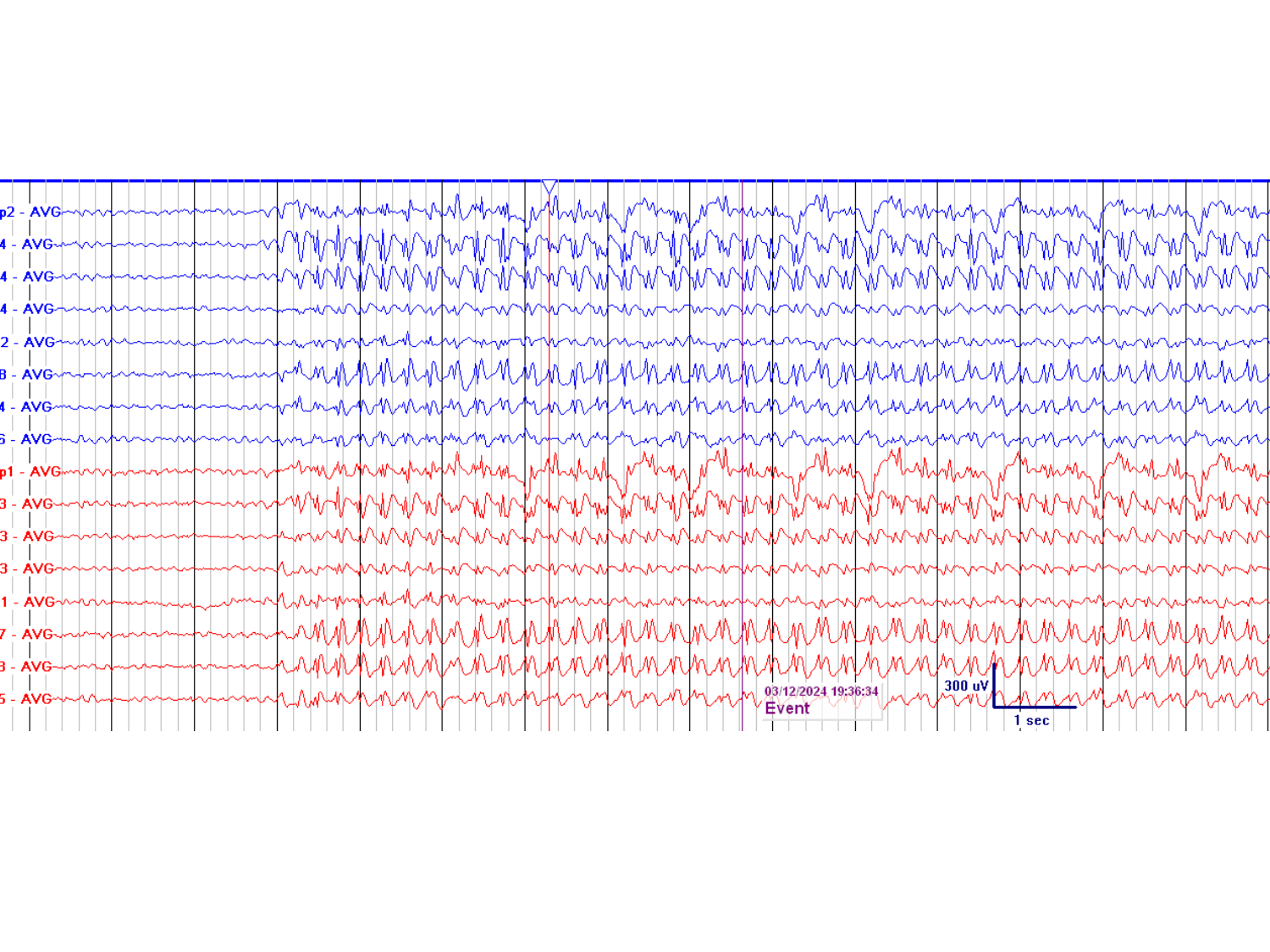

Supplement: Supplementary file 3 — Data S3. [file EPD2-27-1087-s017.pptx]

## Slide 1
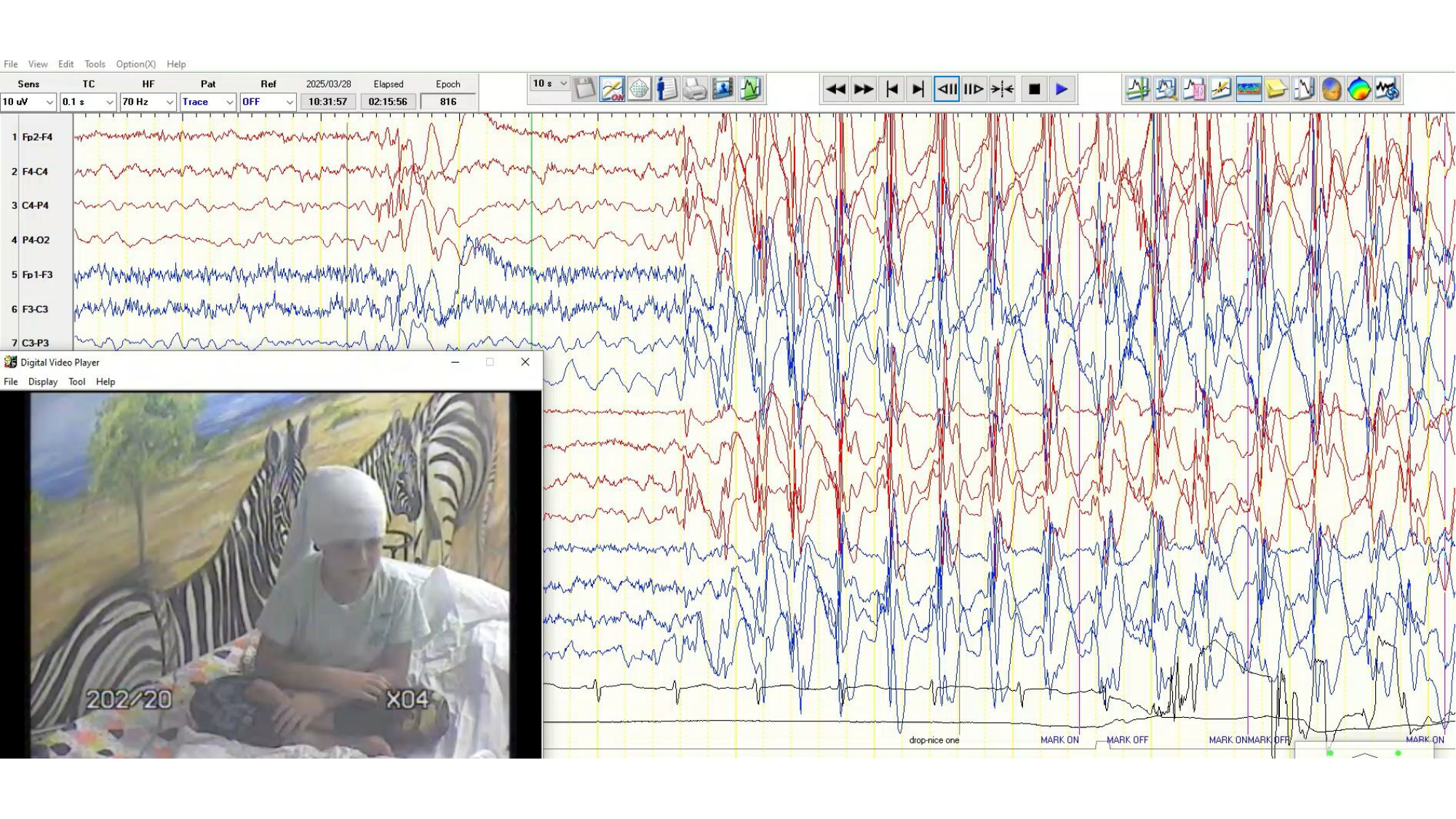

## Slide 2
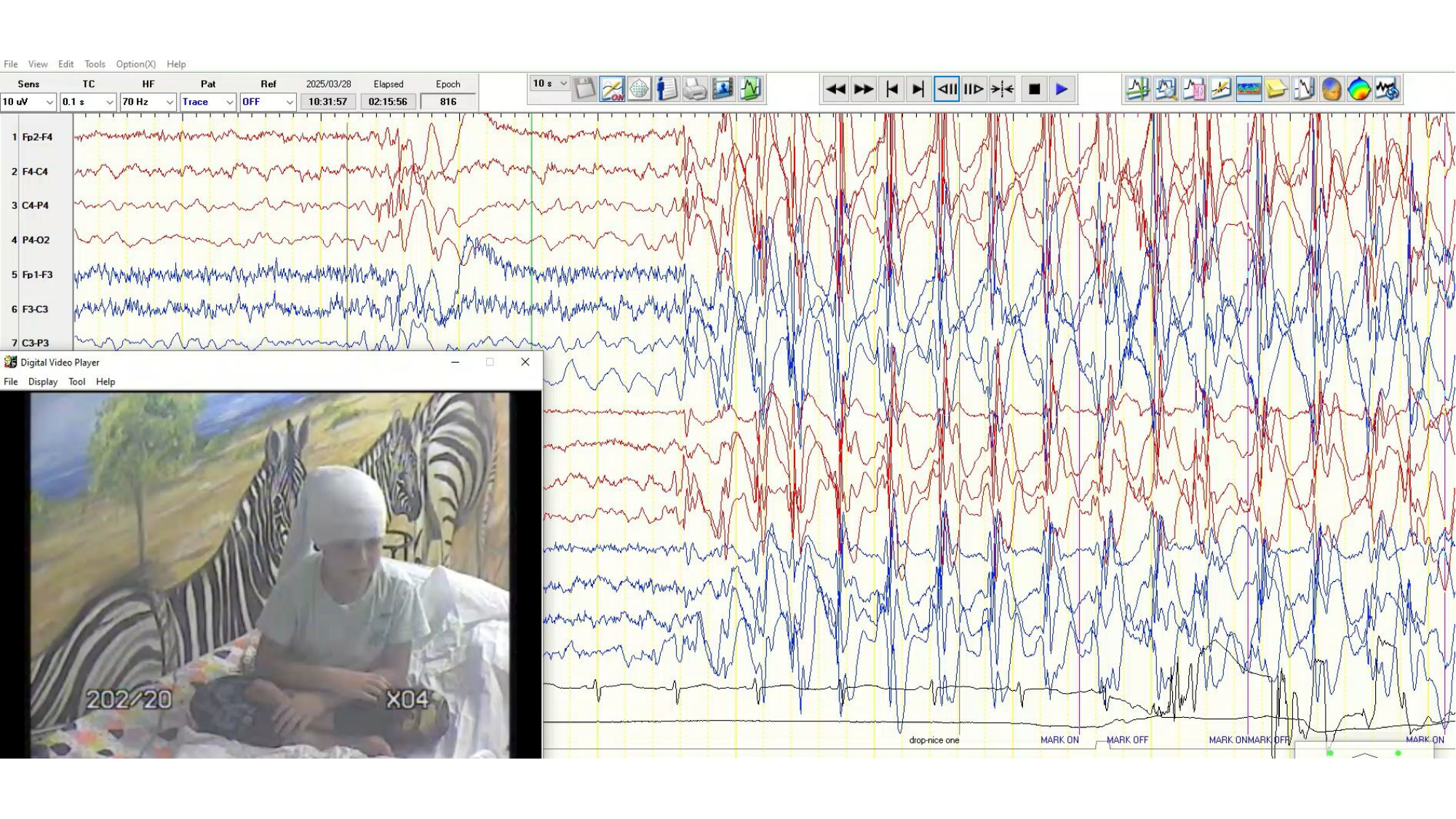

## Slide 3
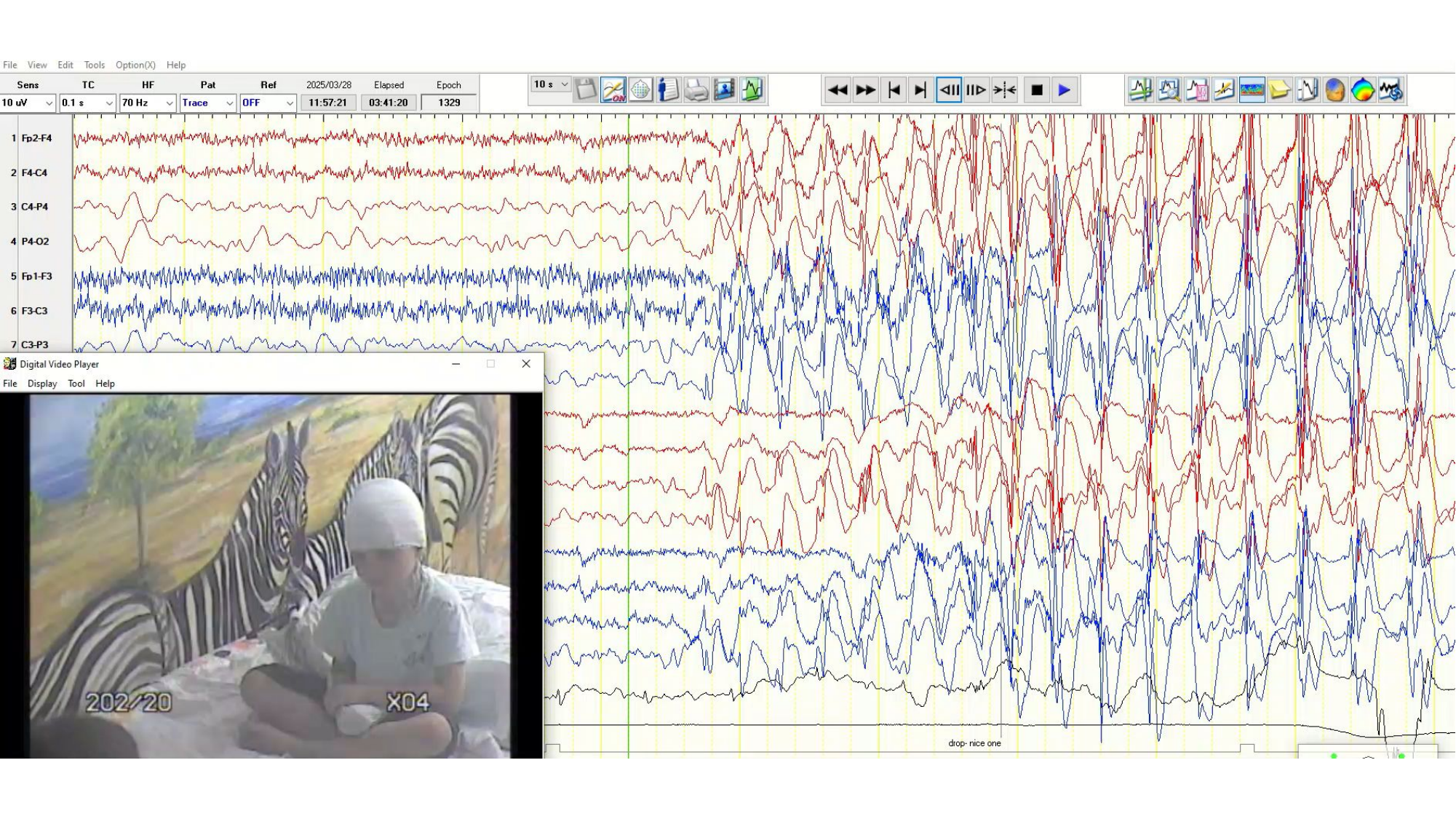

Supplement: Supplementary file 4 — Data S4. [file EPD2-27-1087-s015.pptx]

## Slide 1
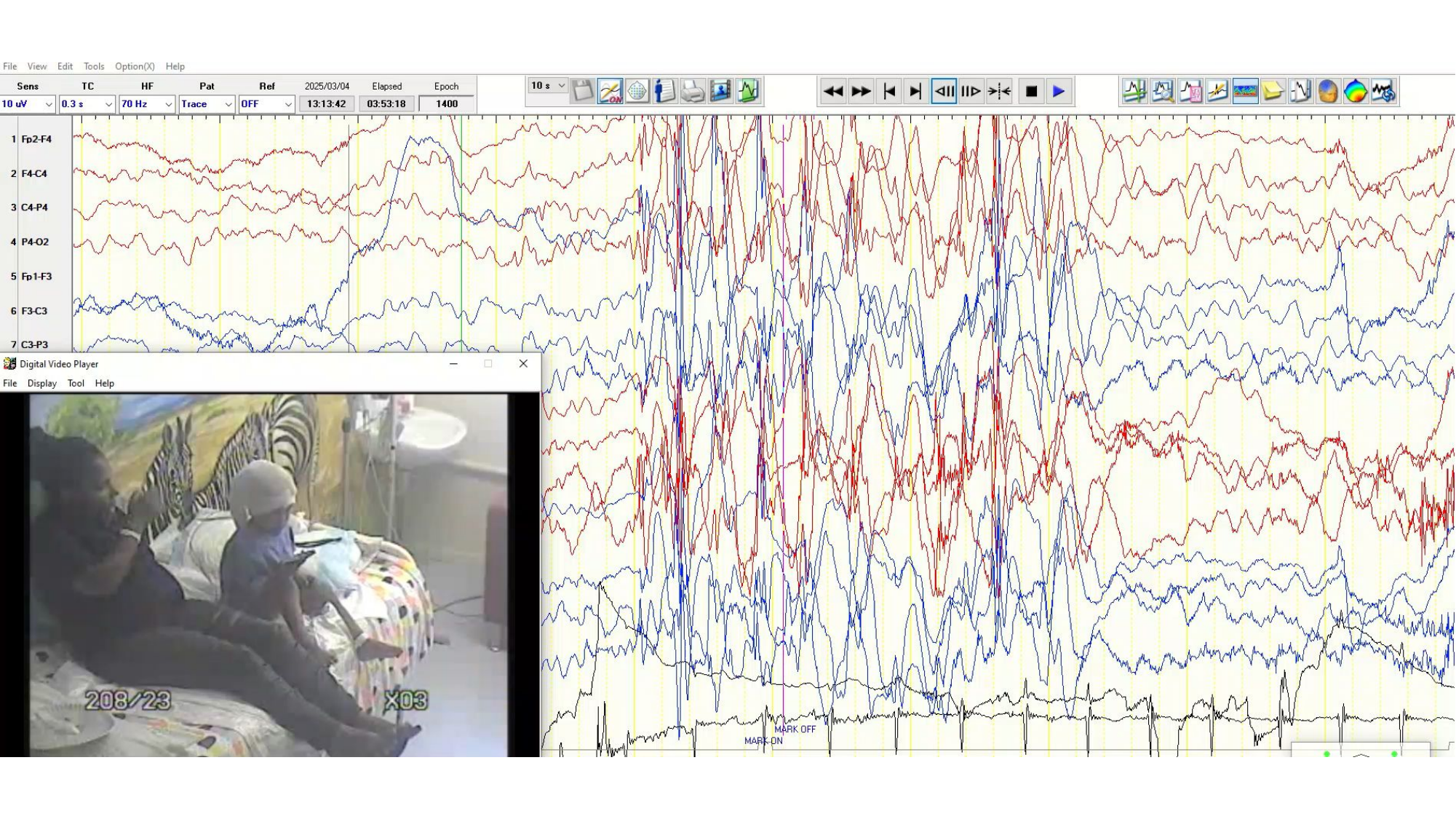

## Slide 2
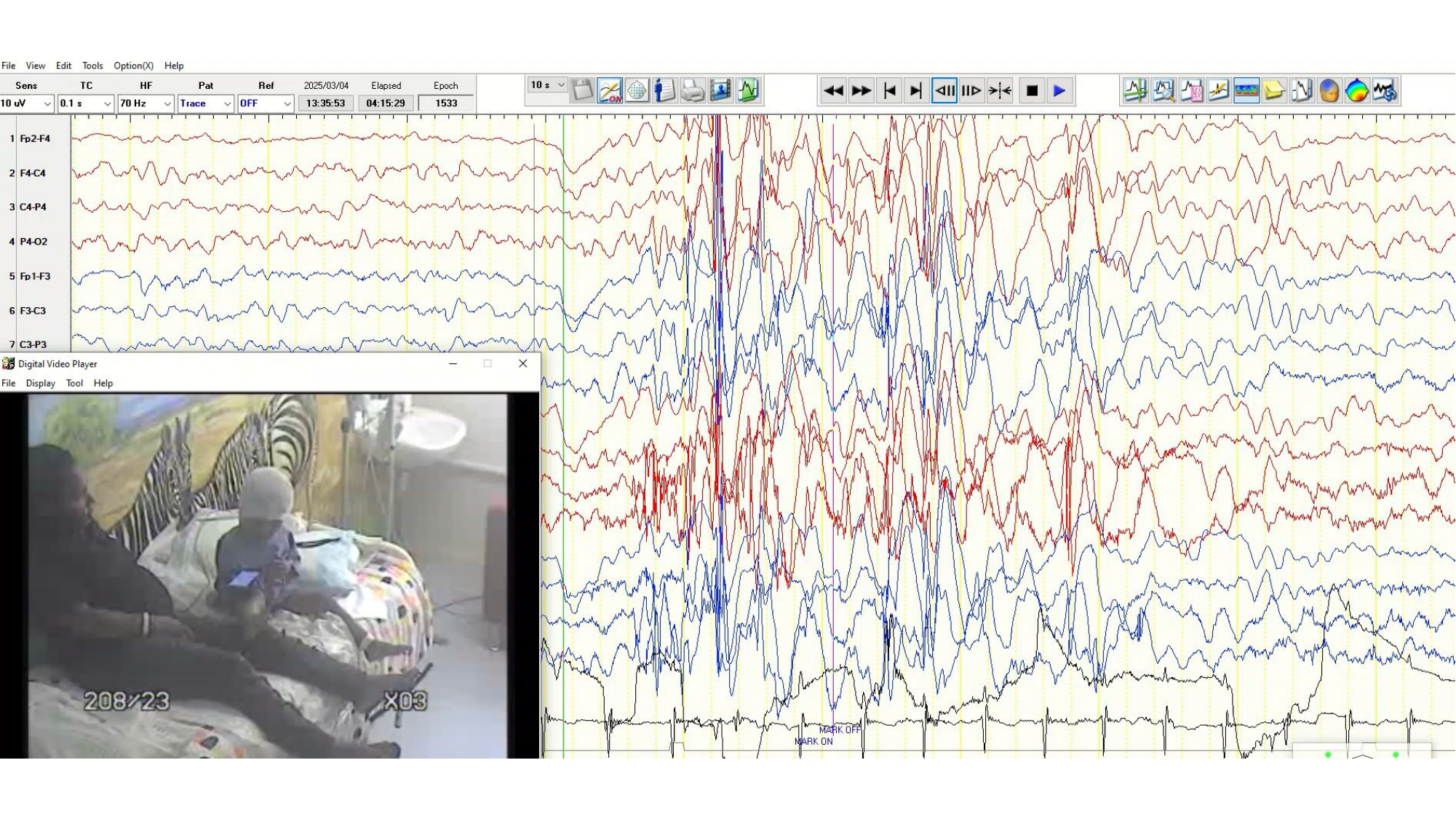

## Slide 3
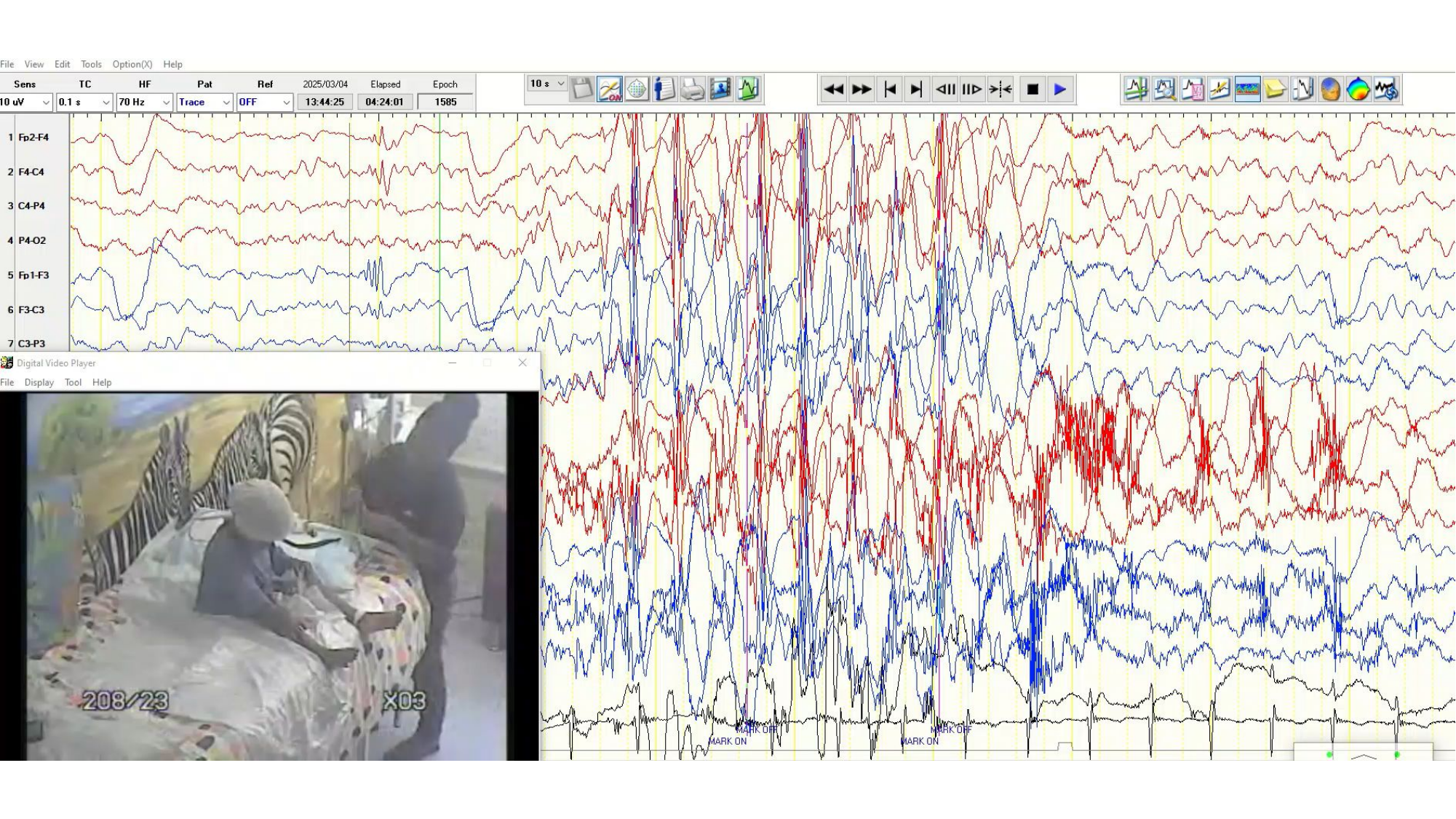

Supplement: Supplementary file 5 — Data S5. [file EPD2-27-1087-s004.pptx]

## Slide 1
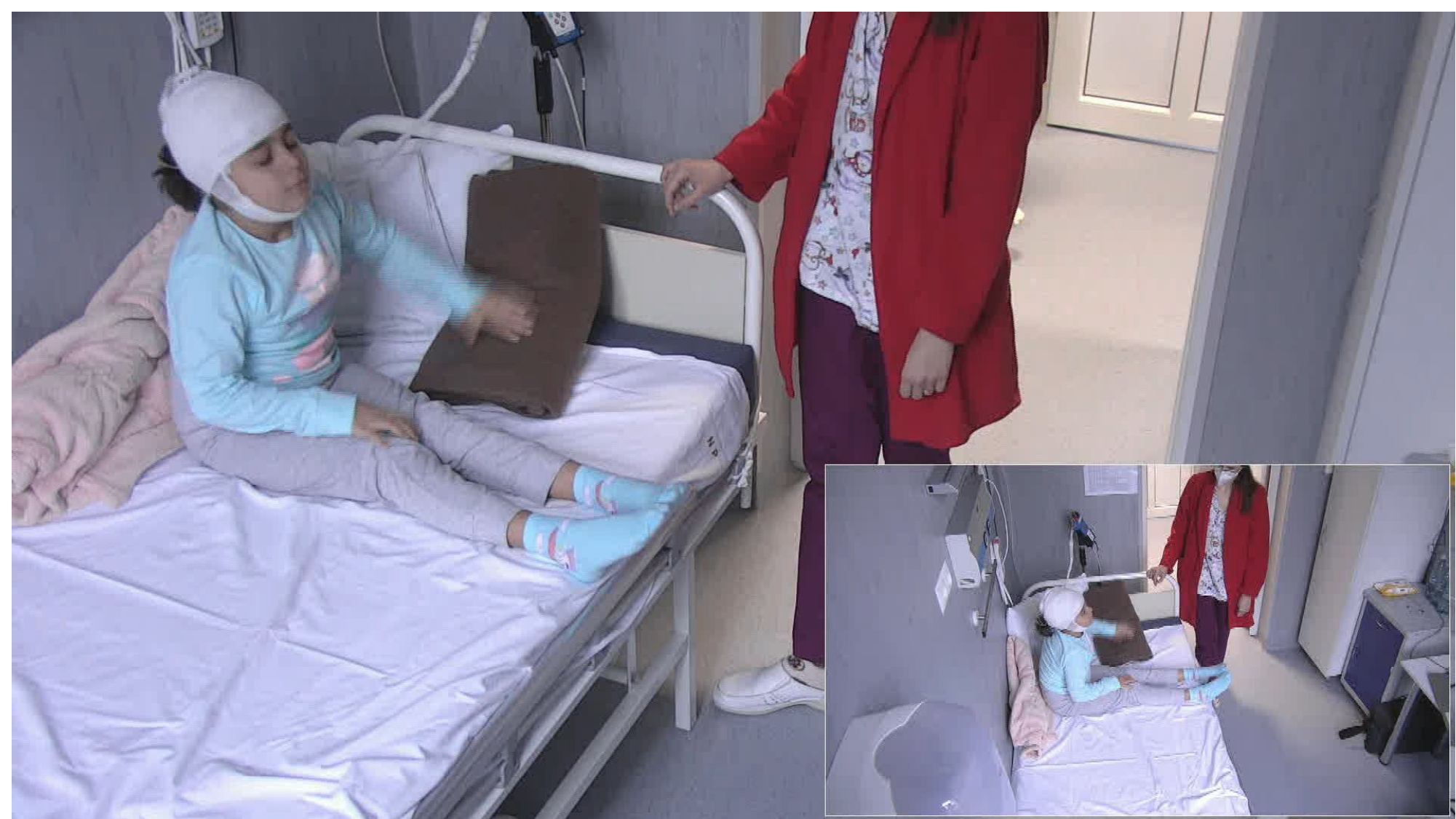

## Slide 2
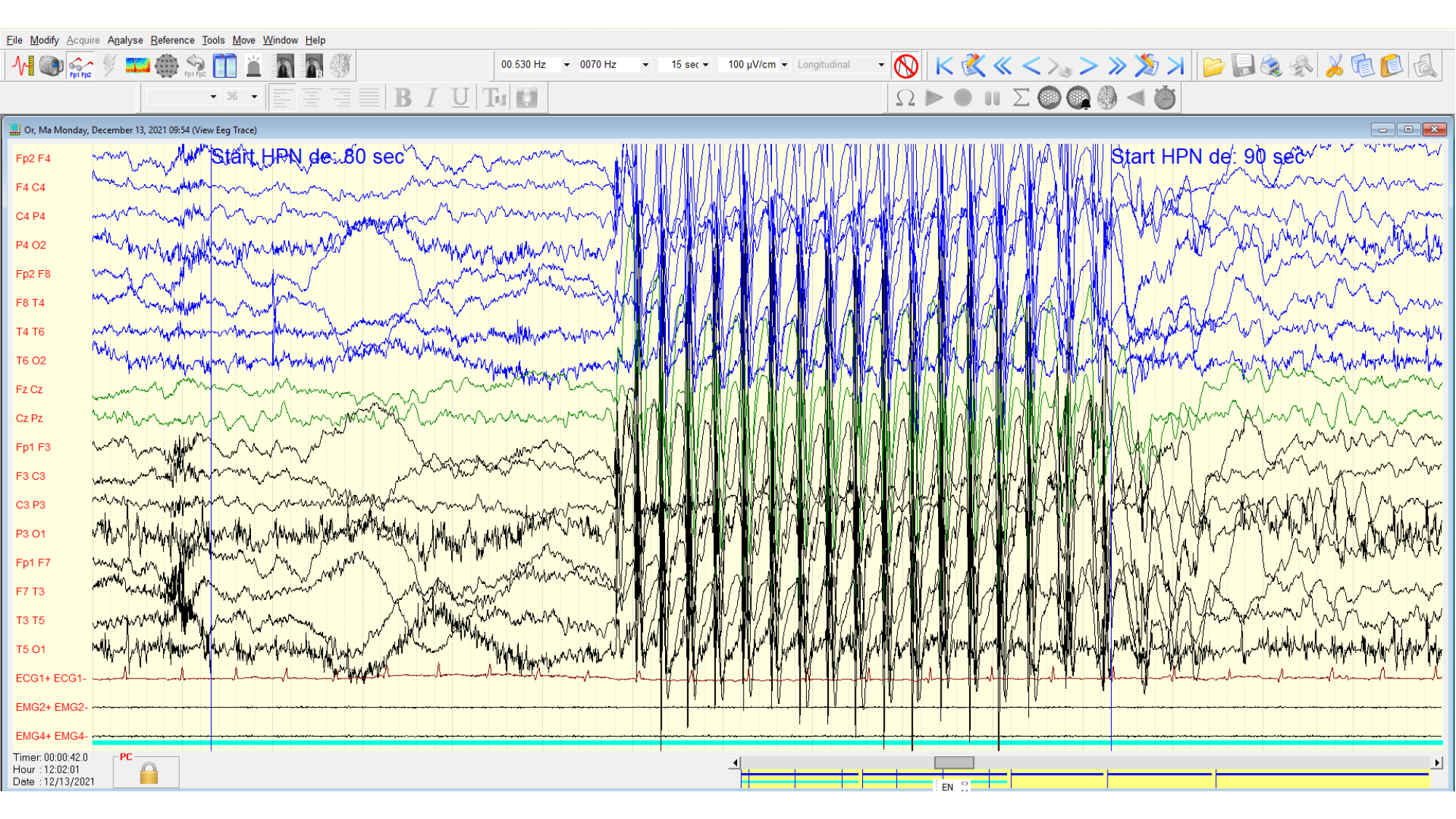

#

## Slide 3
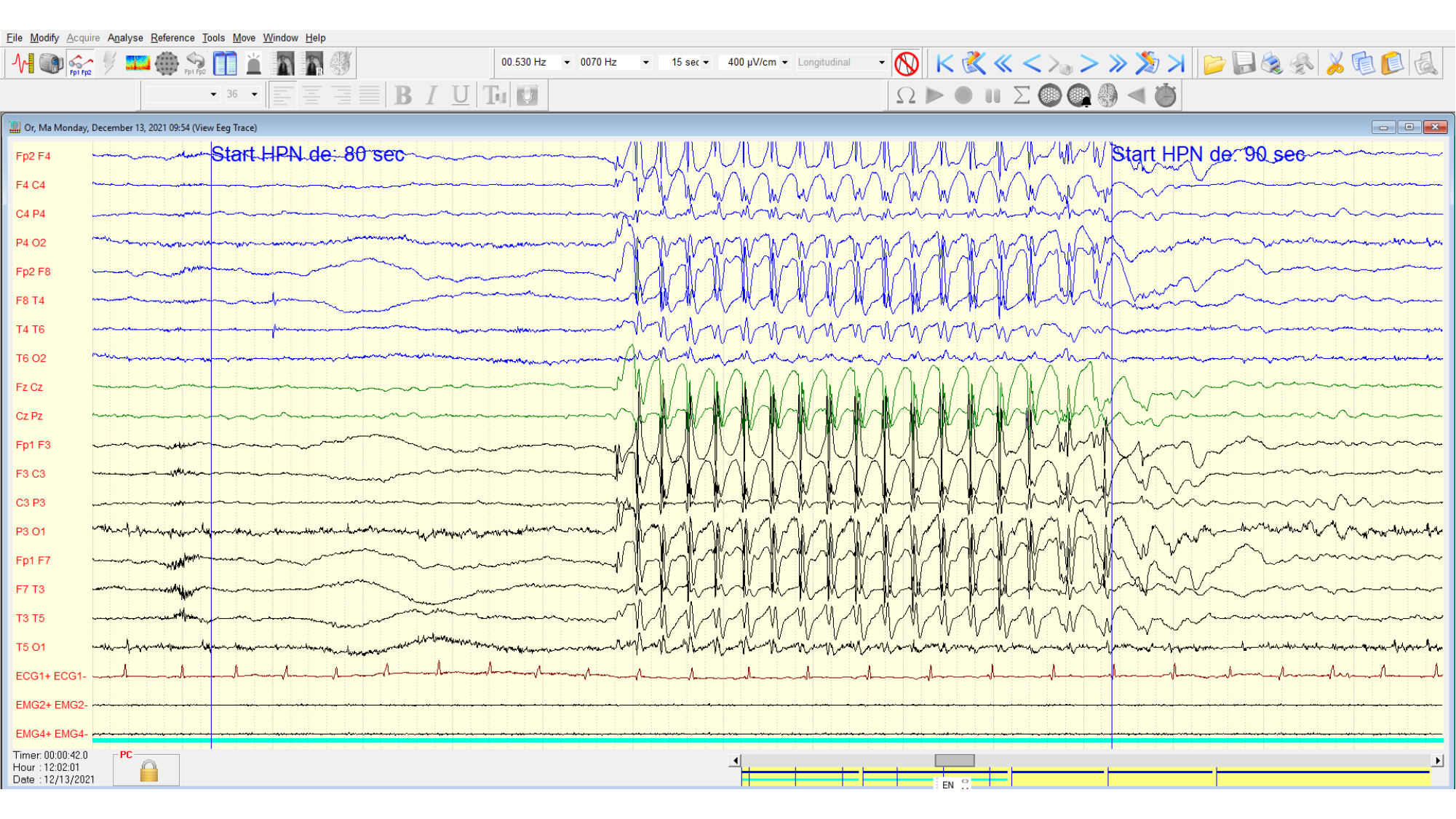

#

Supplement: Supplementary file 6 — Data S6. [file EPD2-27-1087-s005.pptx]

## Slide 1
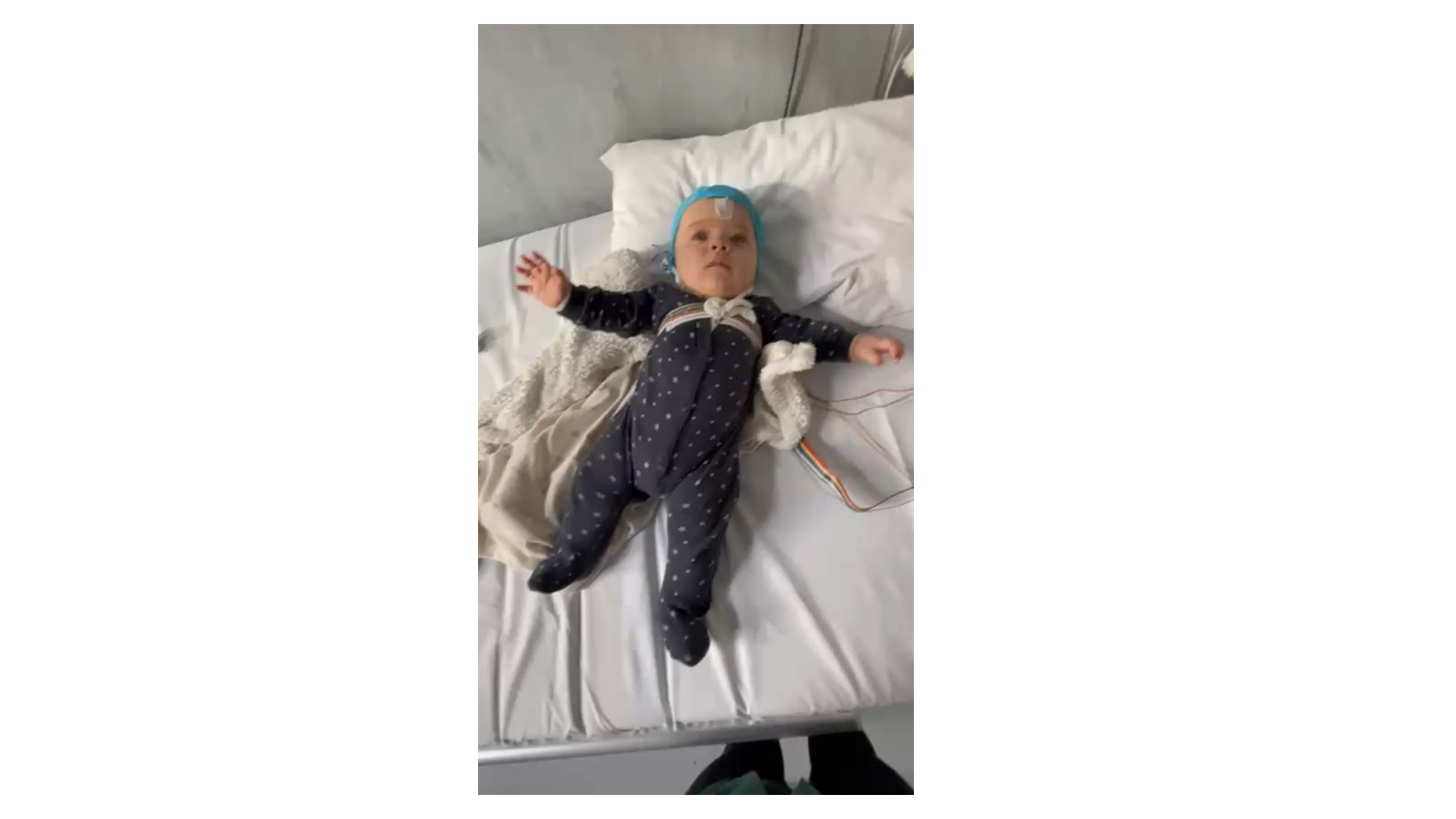

## Slide 2
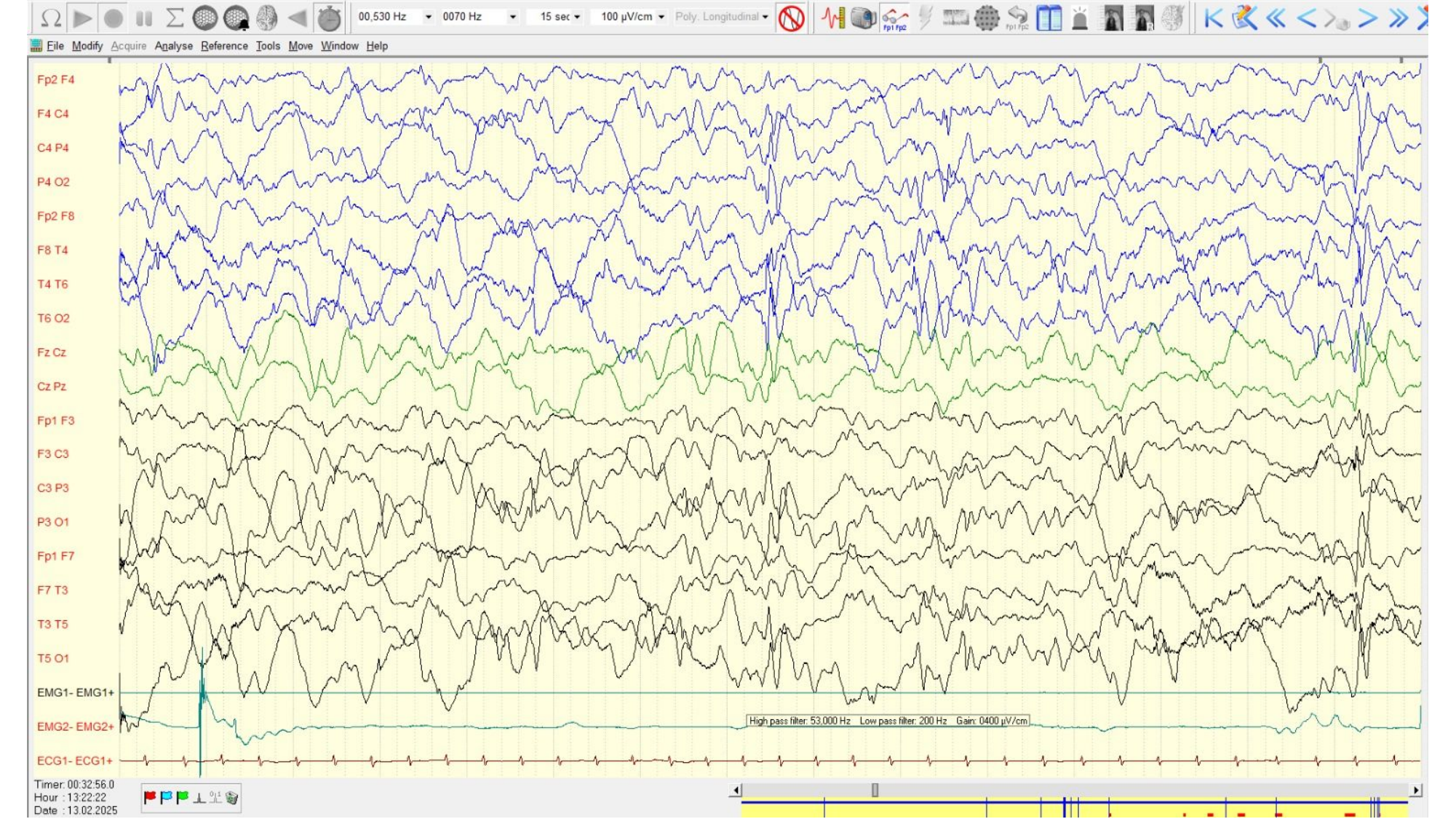

#

## Slide 3
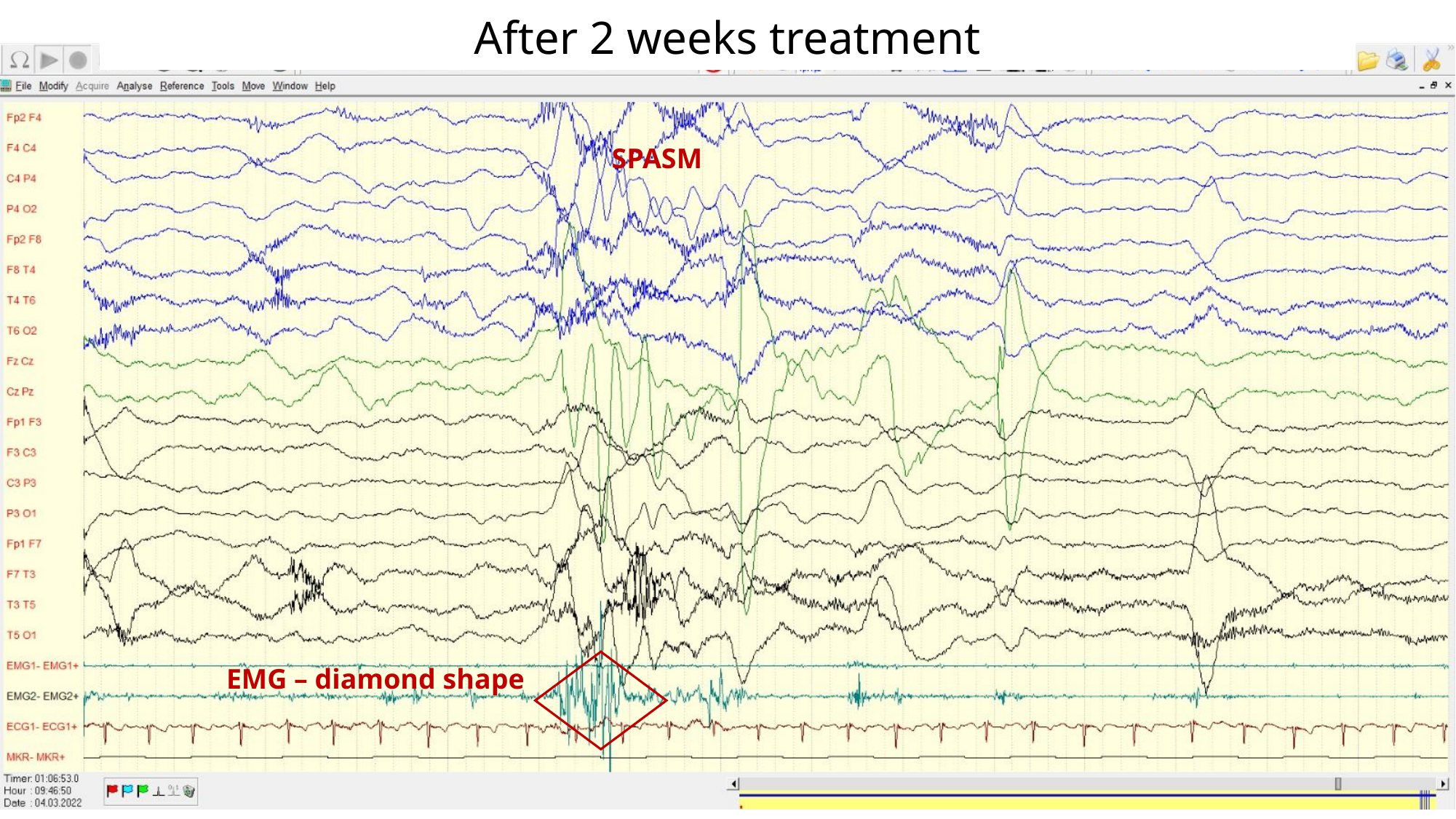

After 2 weeks treatment
SPASM
EMG – diamond shape

Supplement: Supplementary file 7 — Data S7. [file EPD2-27-1087-s018.pptx]

## Slide 1
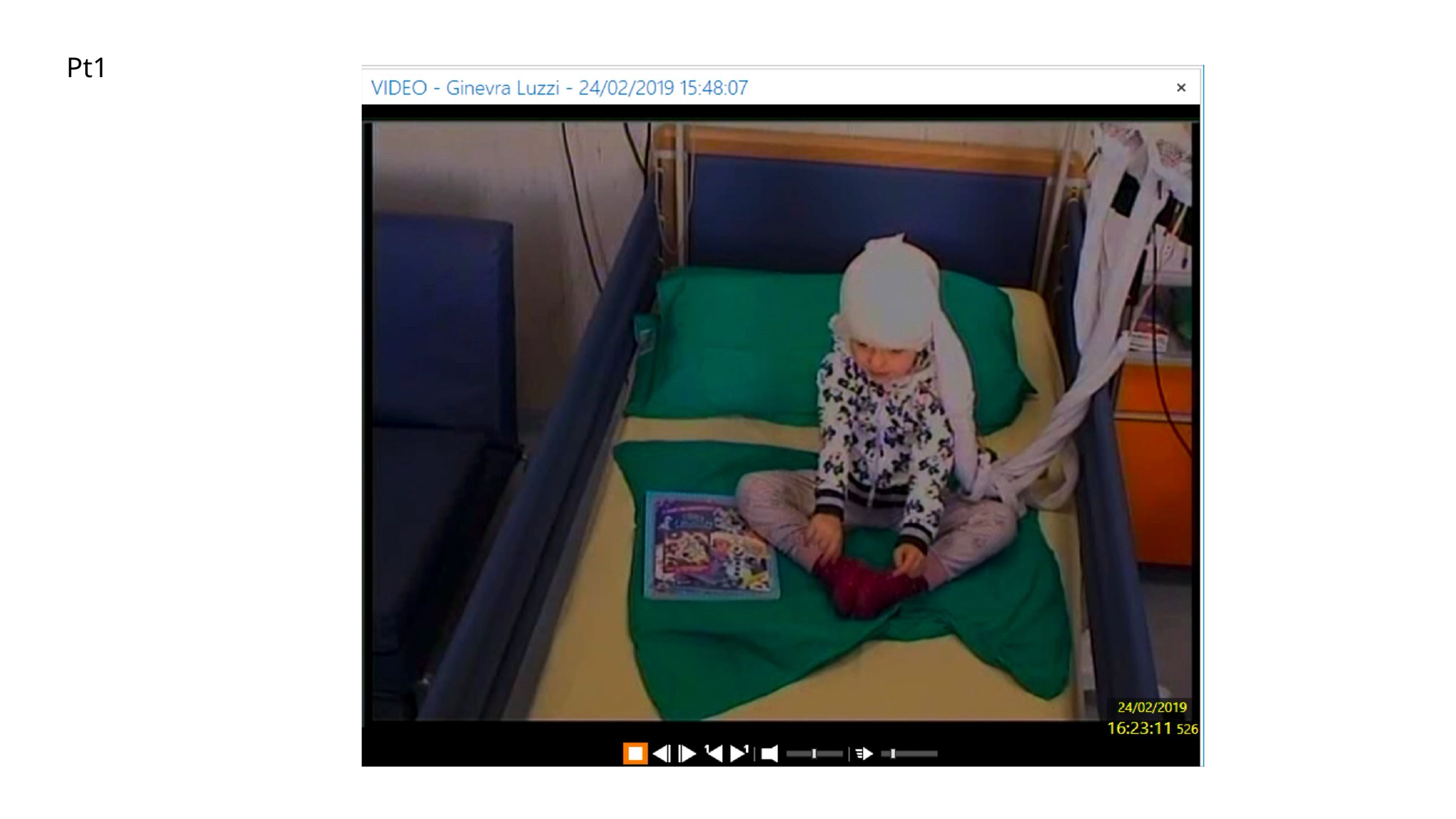

Pt1

## Slide 2
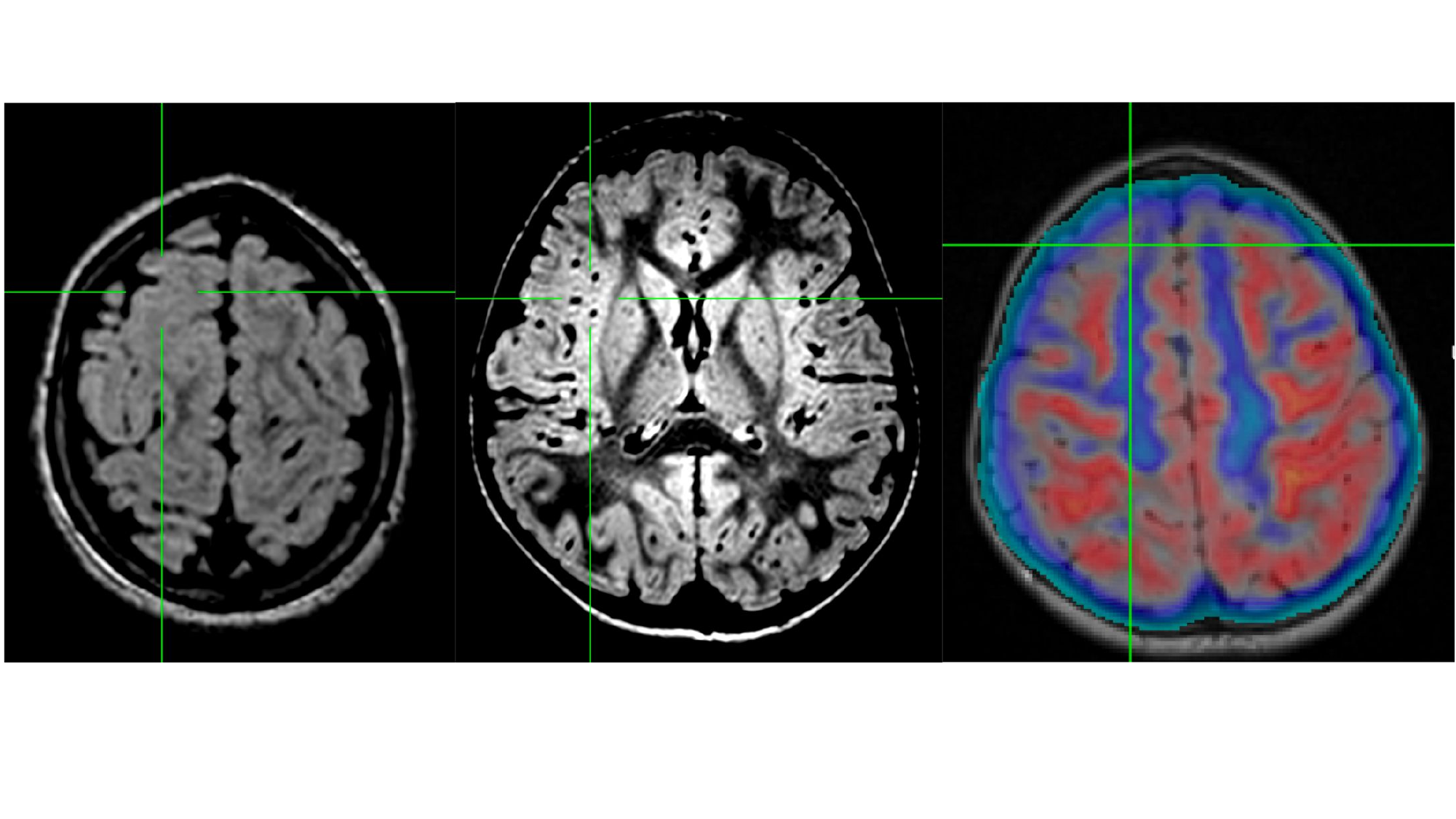

Supplement: Supplementary file 12 — Data S12. [file EPD2-27-1087-s019.pptx]

## Slide 1
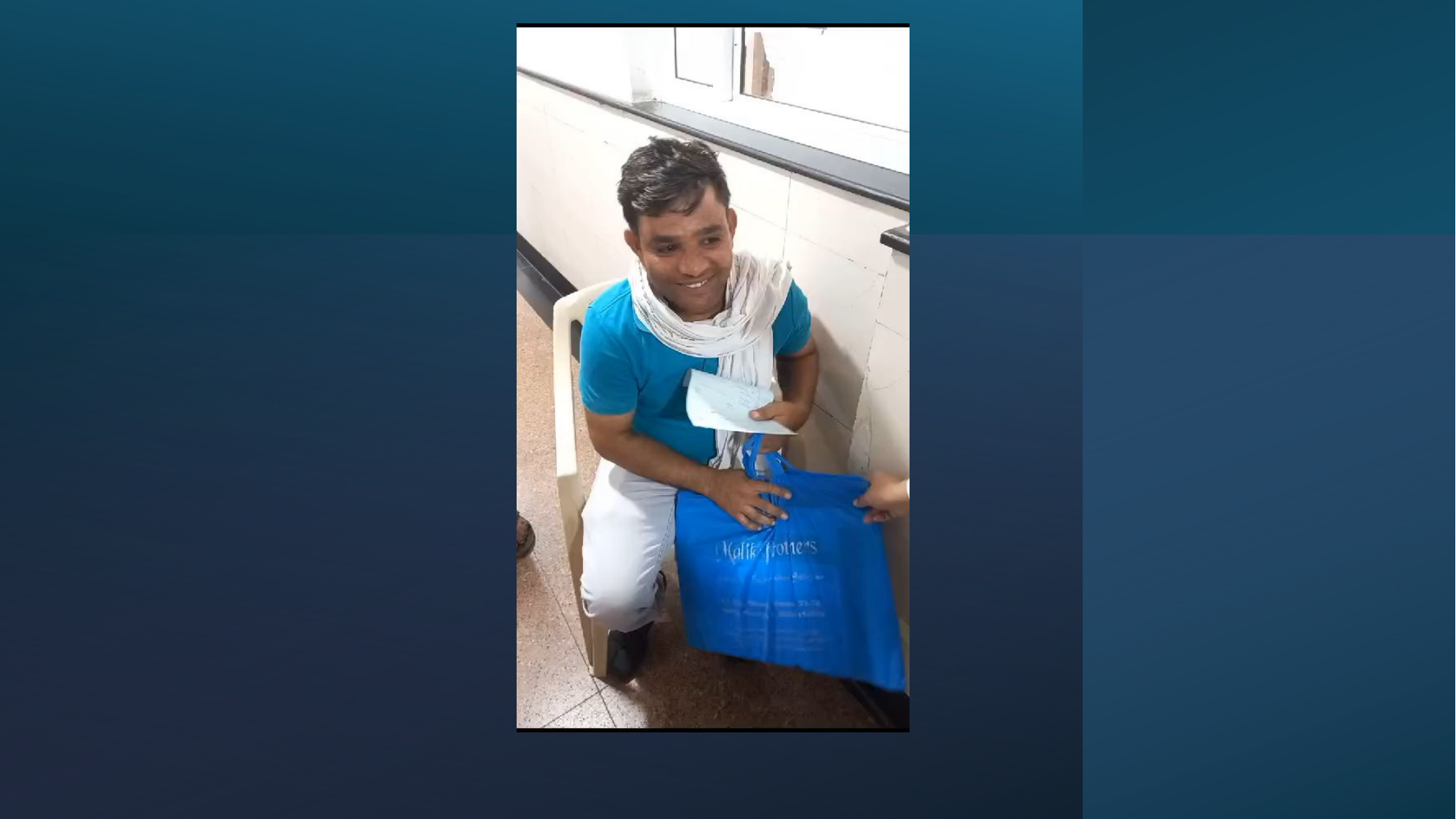

## Slide 2
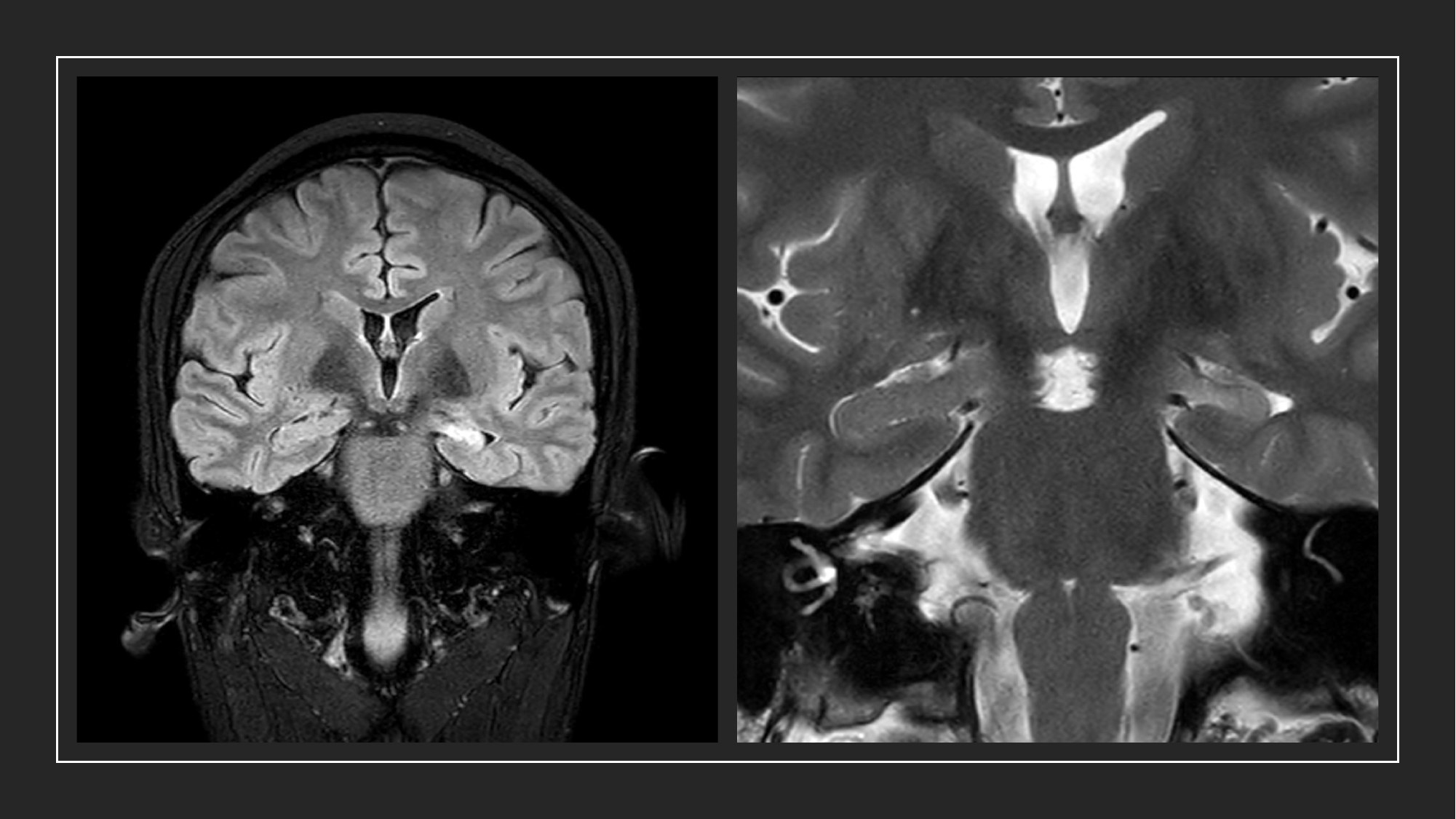

Supplement: Supplementary file 14 — Data S14. [file EPD2-27-1087-s003.pptx]

## Slide 1
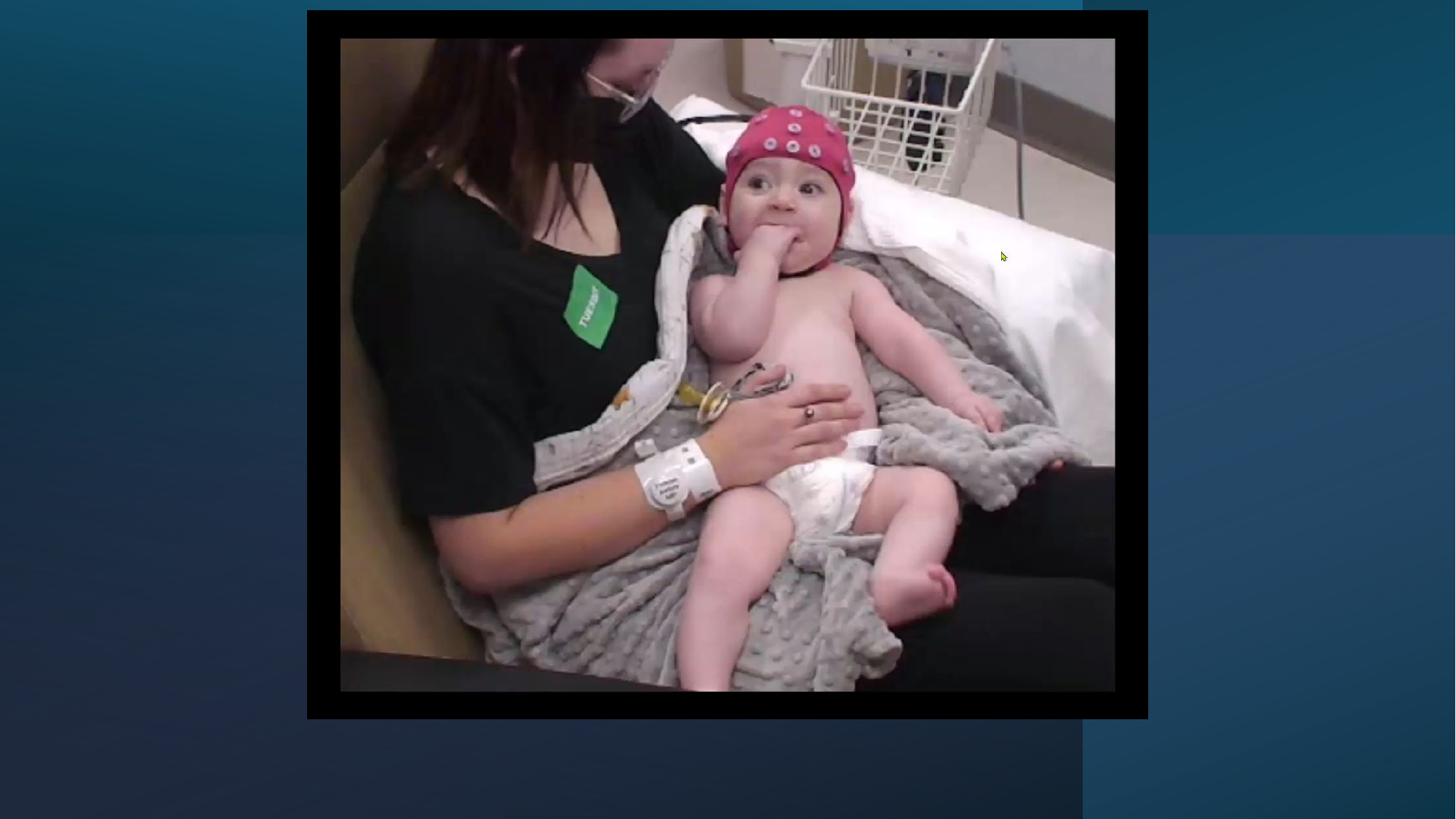

## Slide 2
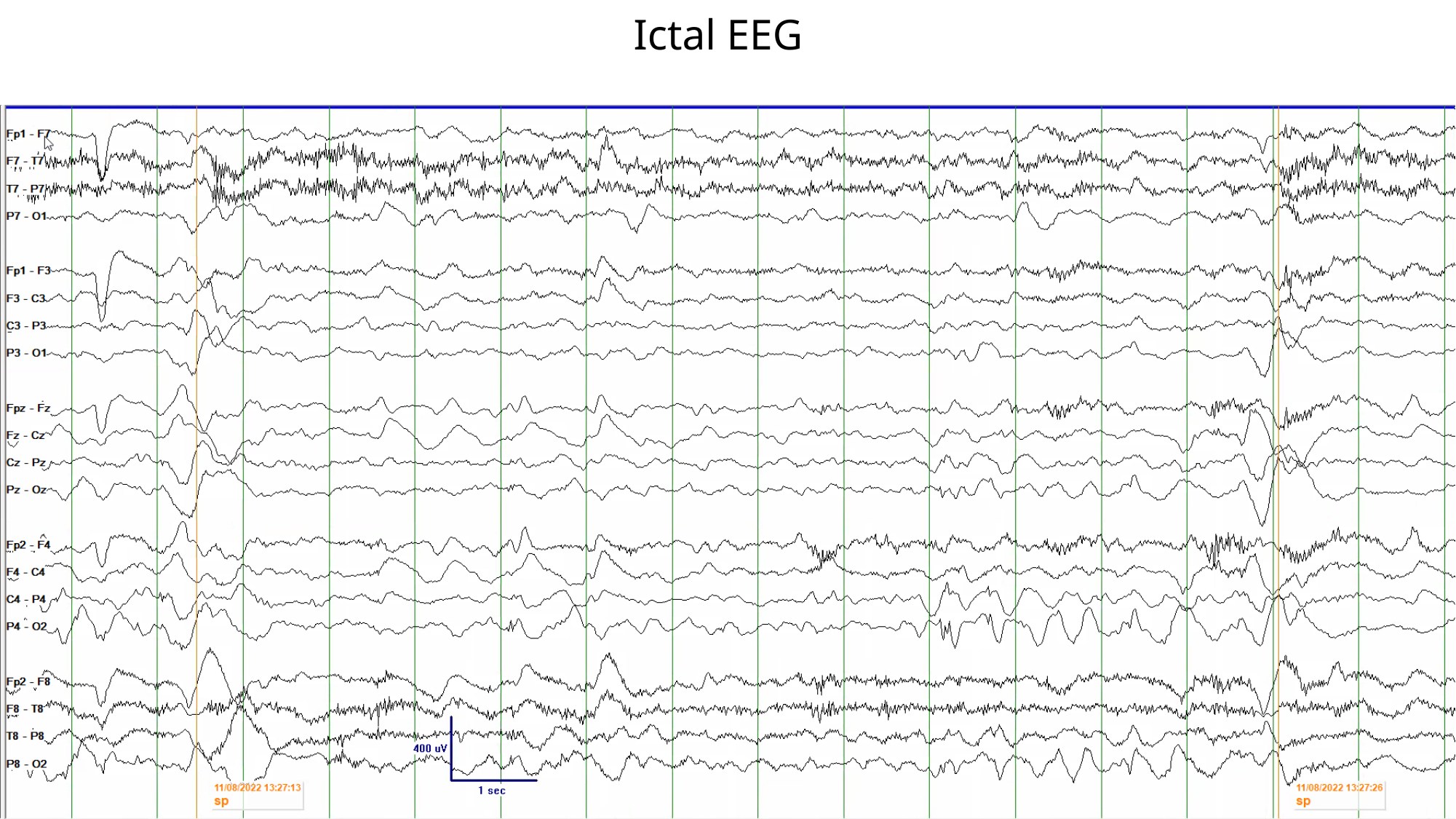

Ictal EEG

## Slide 3
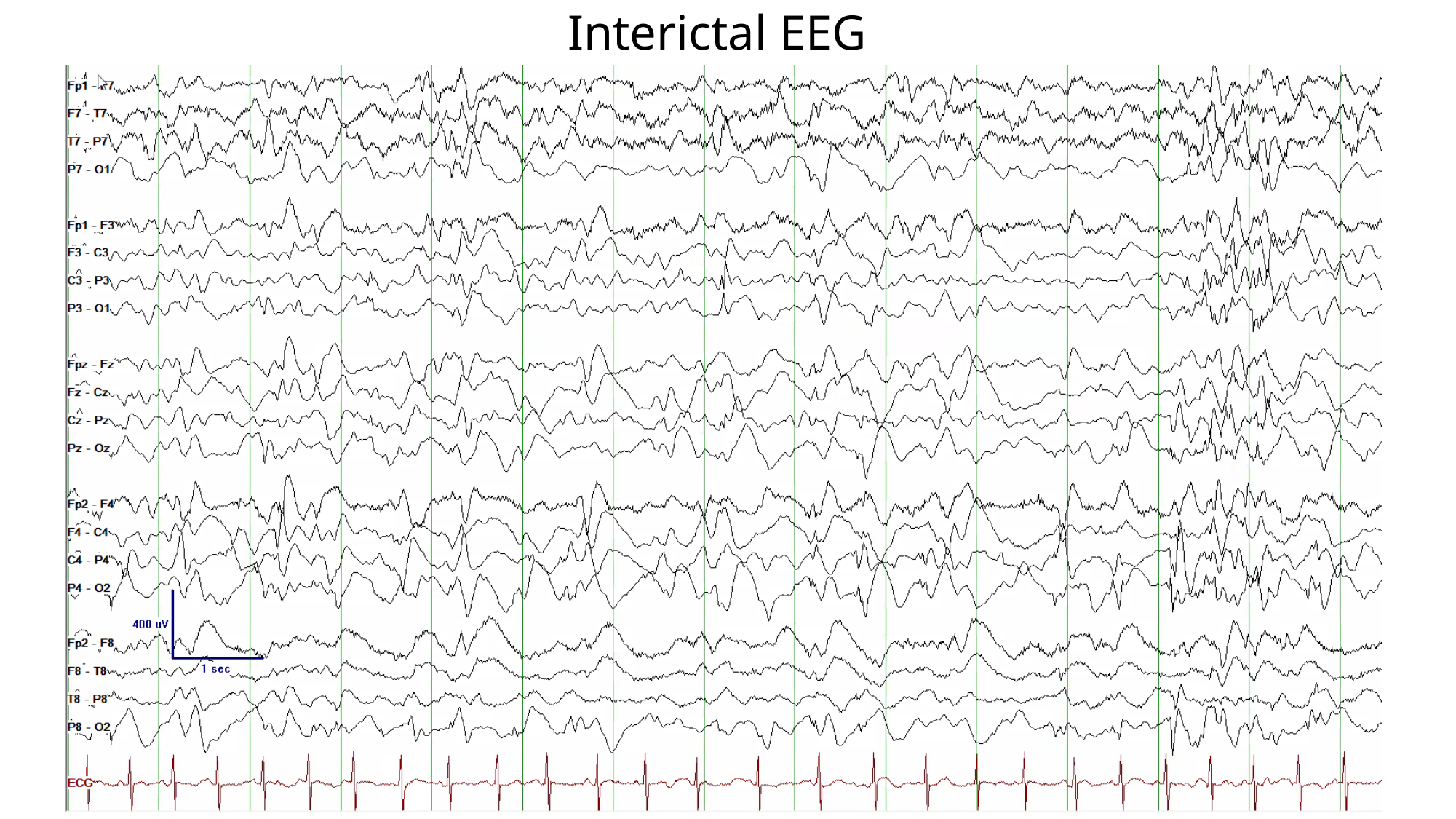

Interictal EEG

Supplement: Supplementary file 15 — Data S15. [file EPD2-27-1087-s009.pptx]

## Slide 1
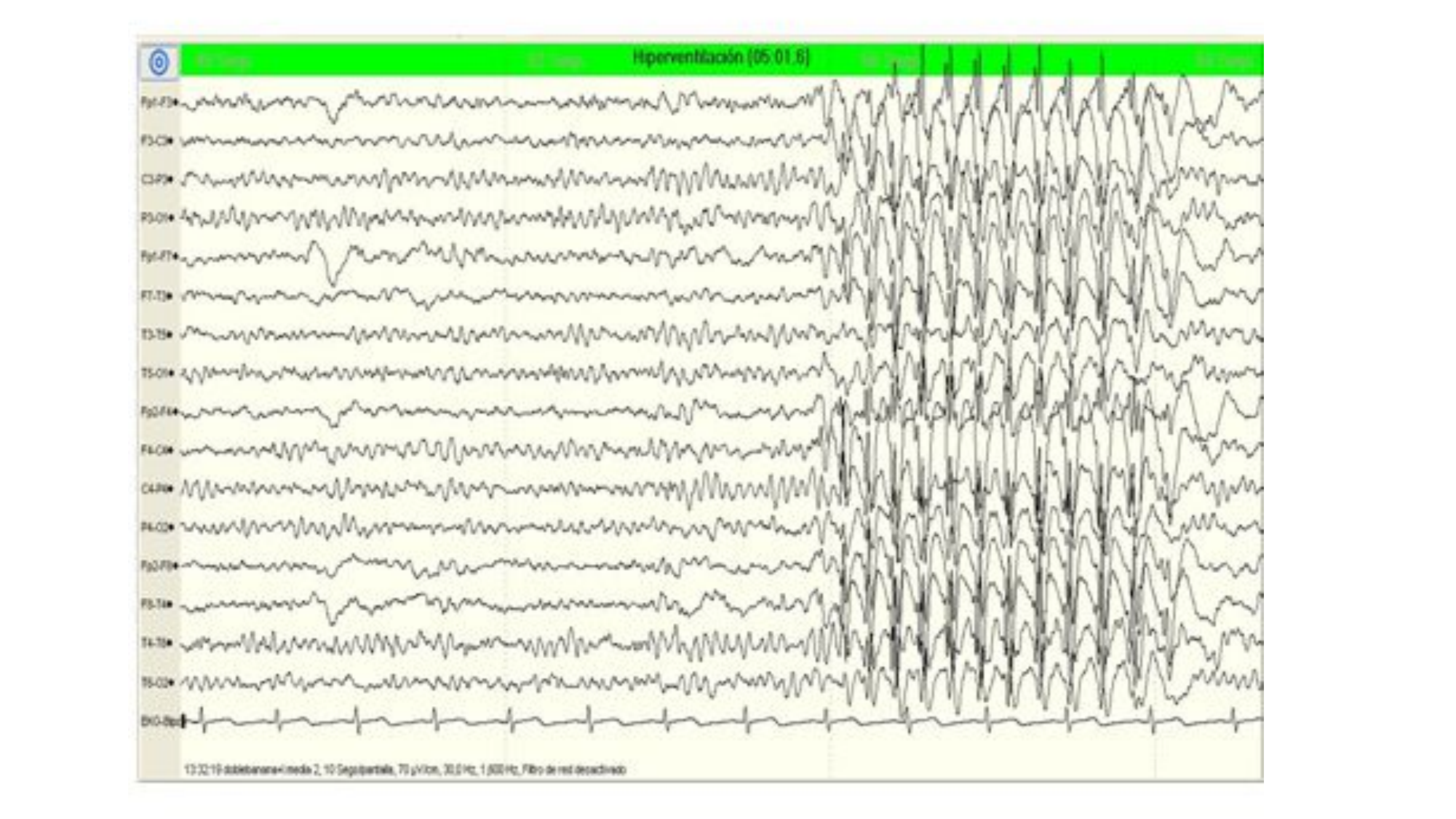

## Slide 2
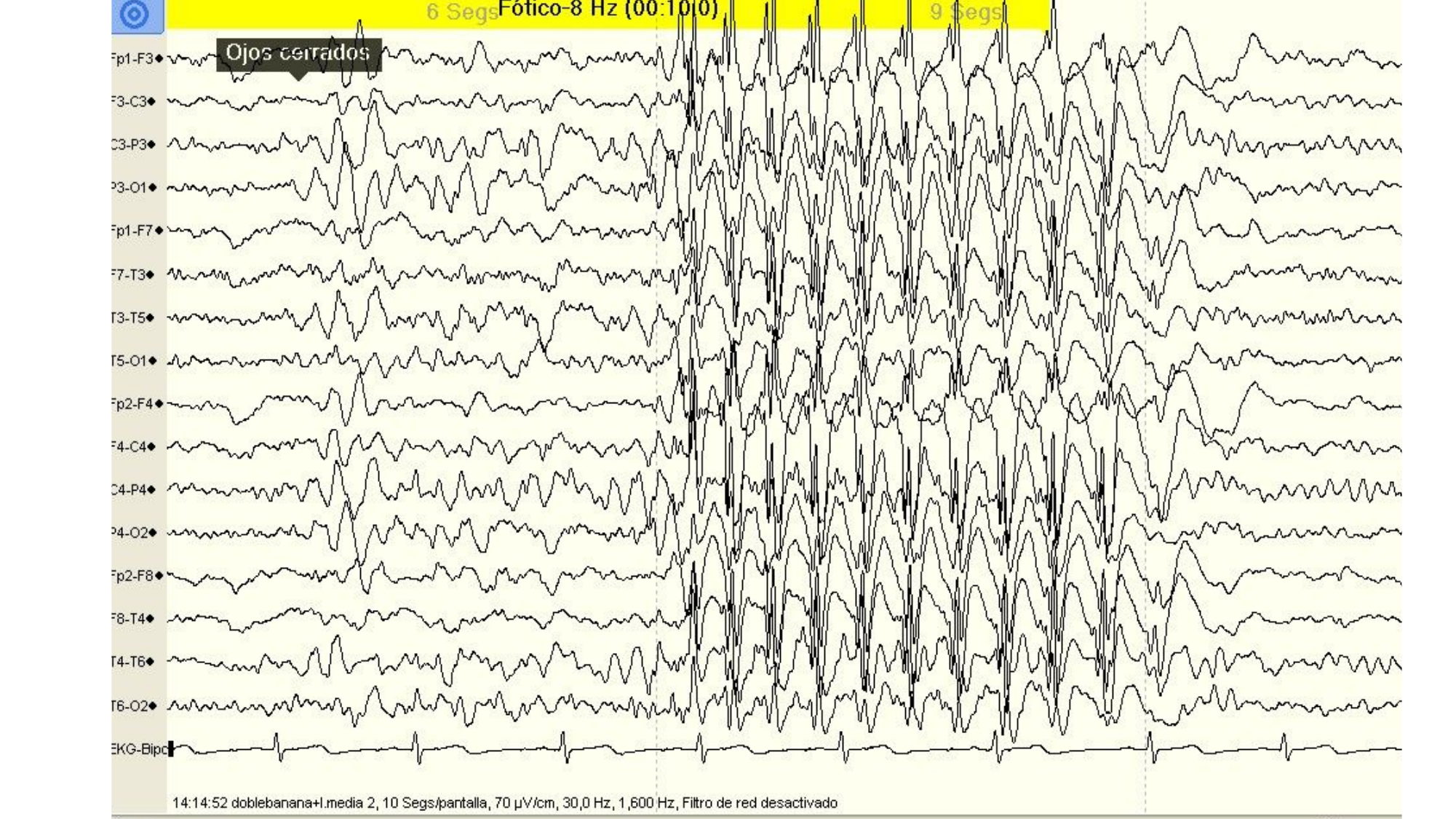

Supplement: Supplementary file 17 — Data S17. [file EPD2-27-1087-s006.pptx]

## Slide 1
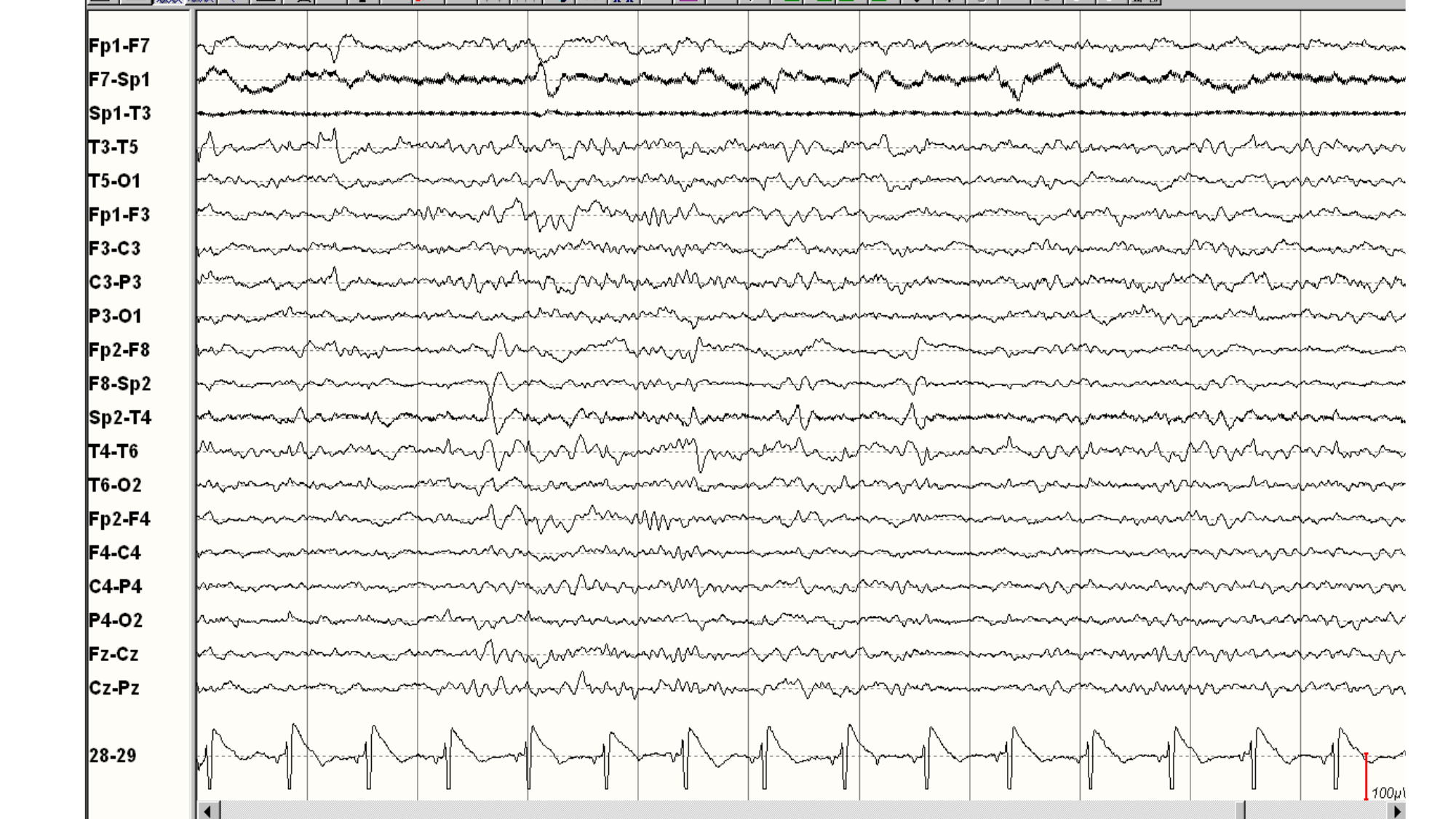

Supplement: Supplementary file 18 — Data S18. [file EPD2-27-1087-s001.pptx]

## Slide 1
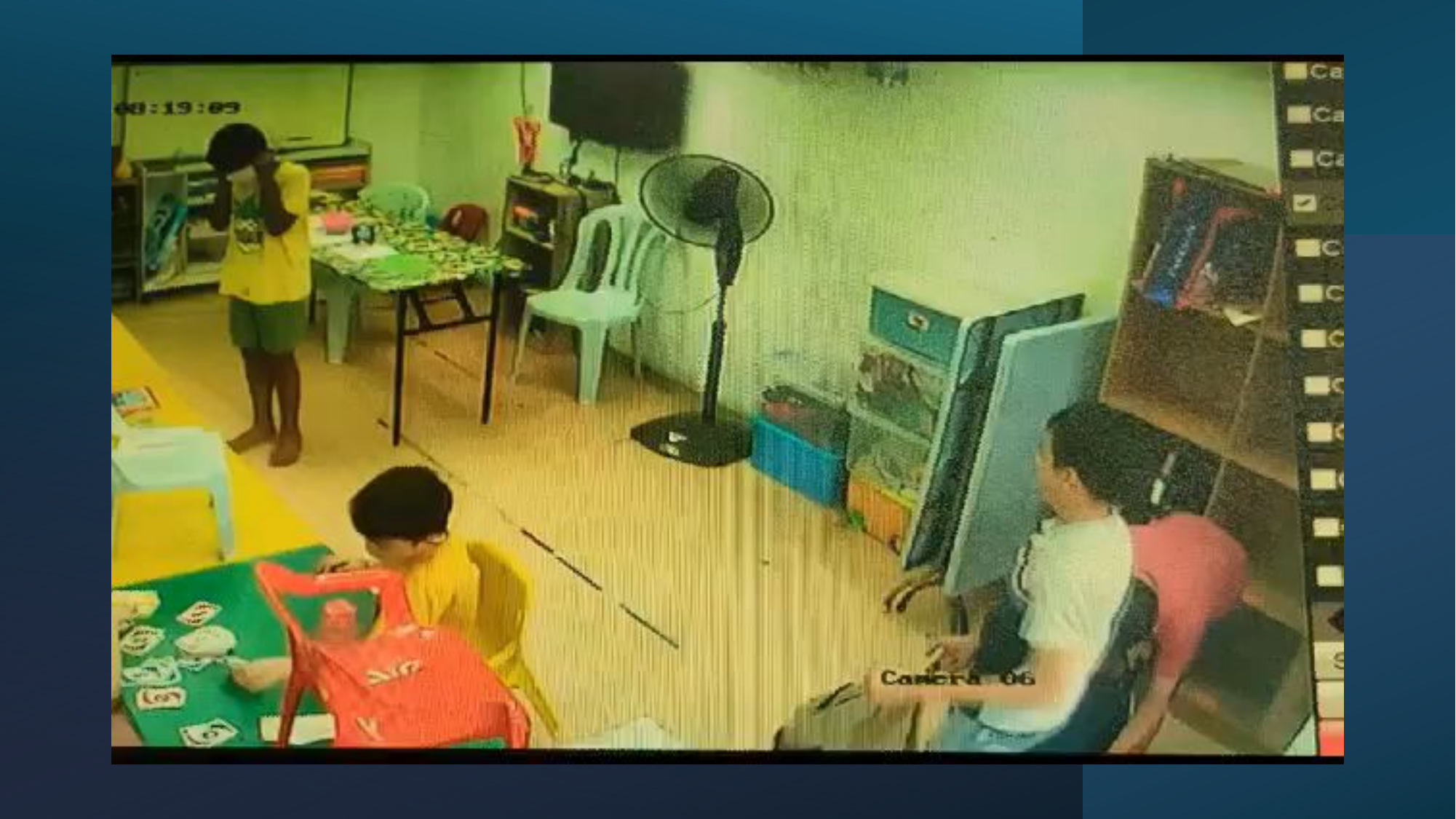

Supplement: Supplementary file 19 — Data S19. [file EPD2-27-1087-s016.pptx]

## Slide 1
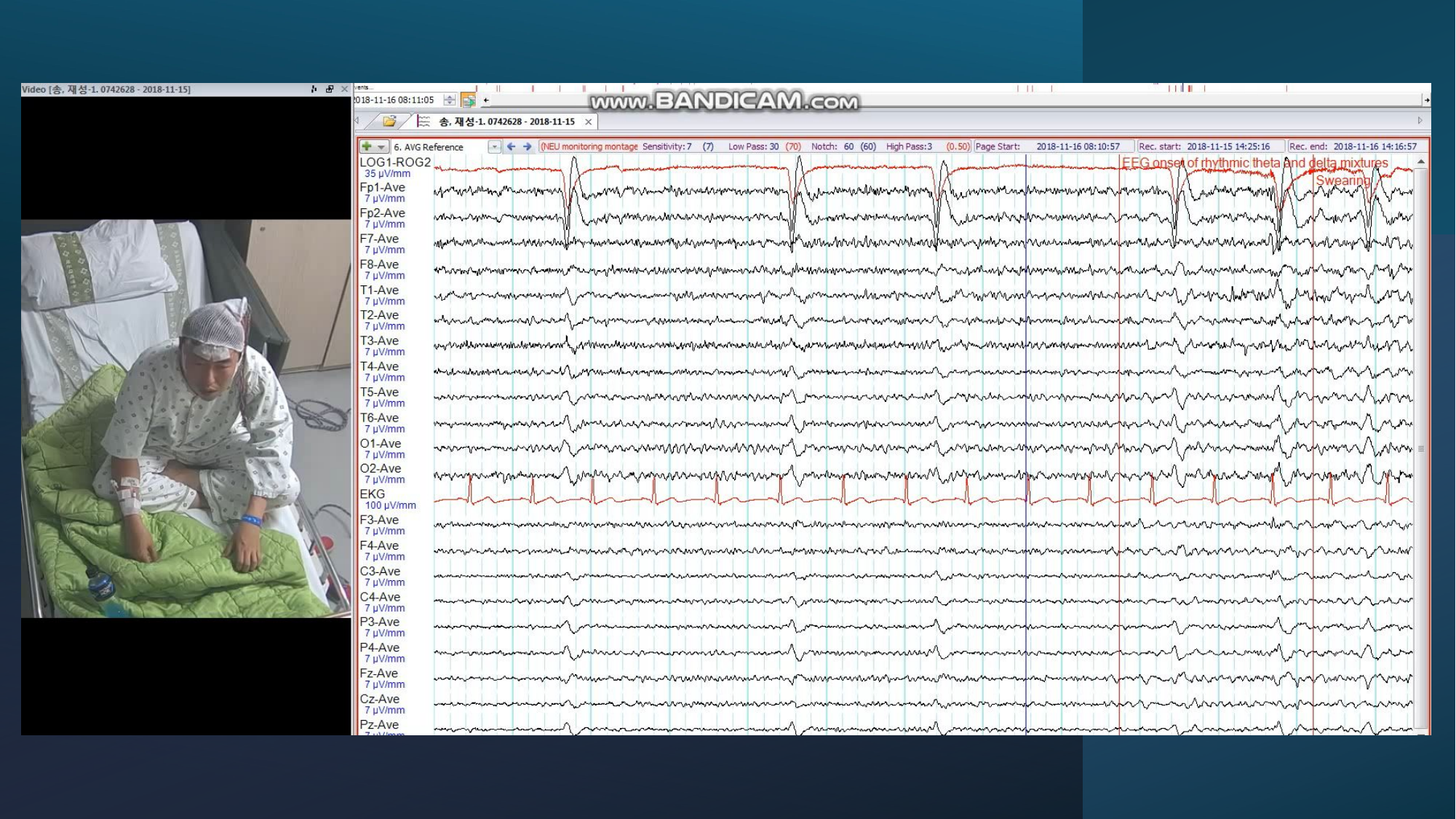

Supplement: Supplementary file 20 — Data S20. [file EPD2-27-1087-s026.pptx]

## Slide 1
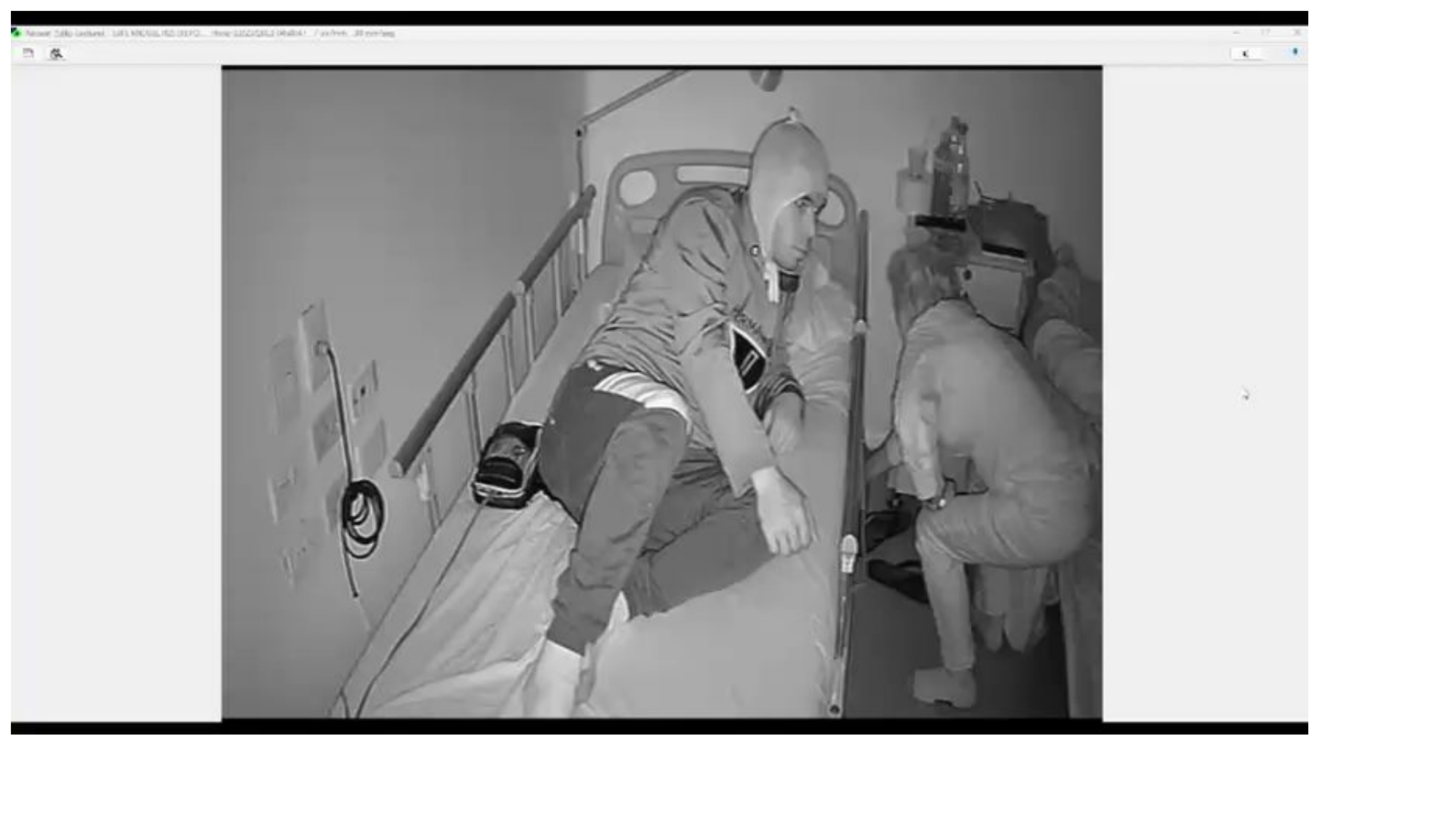

## Slide 2
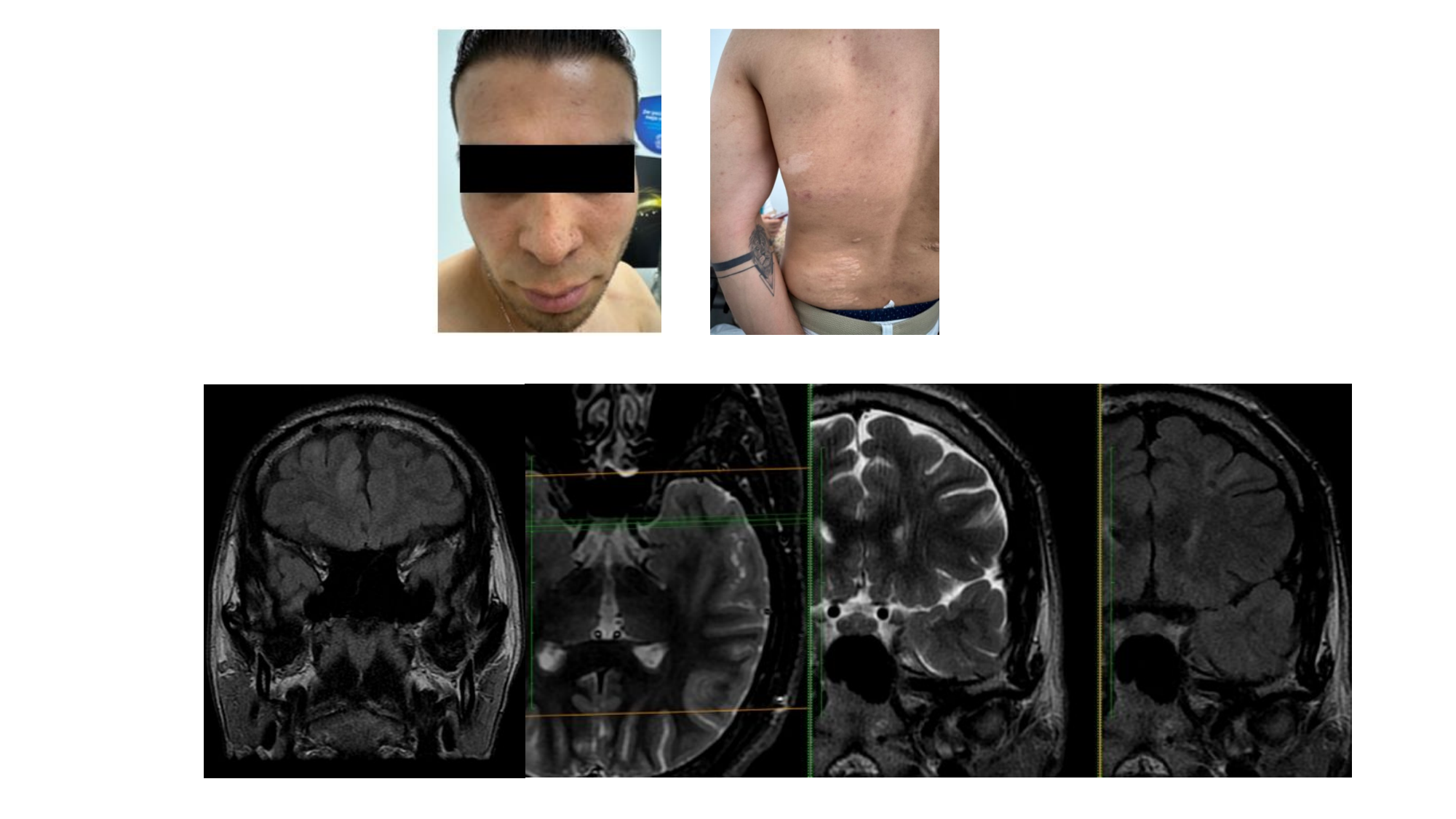

Supplement: Supplementary file 21 — Data S21. [file EPD2-27-1087-s011.pptx]

## Slide 1
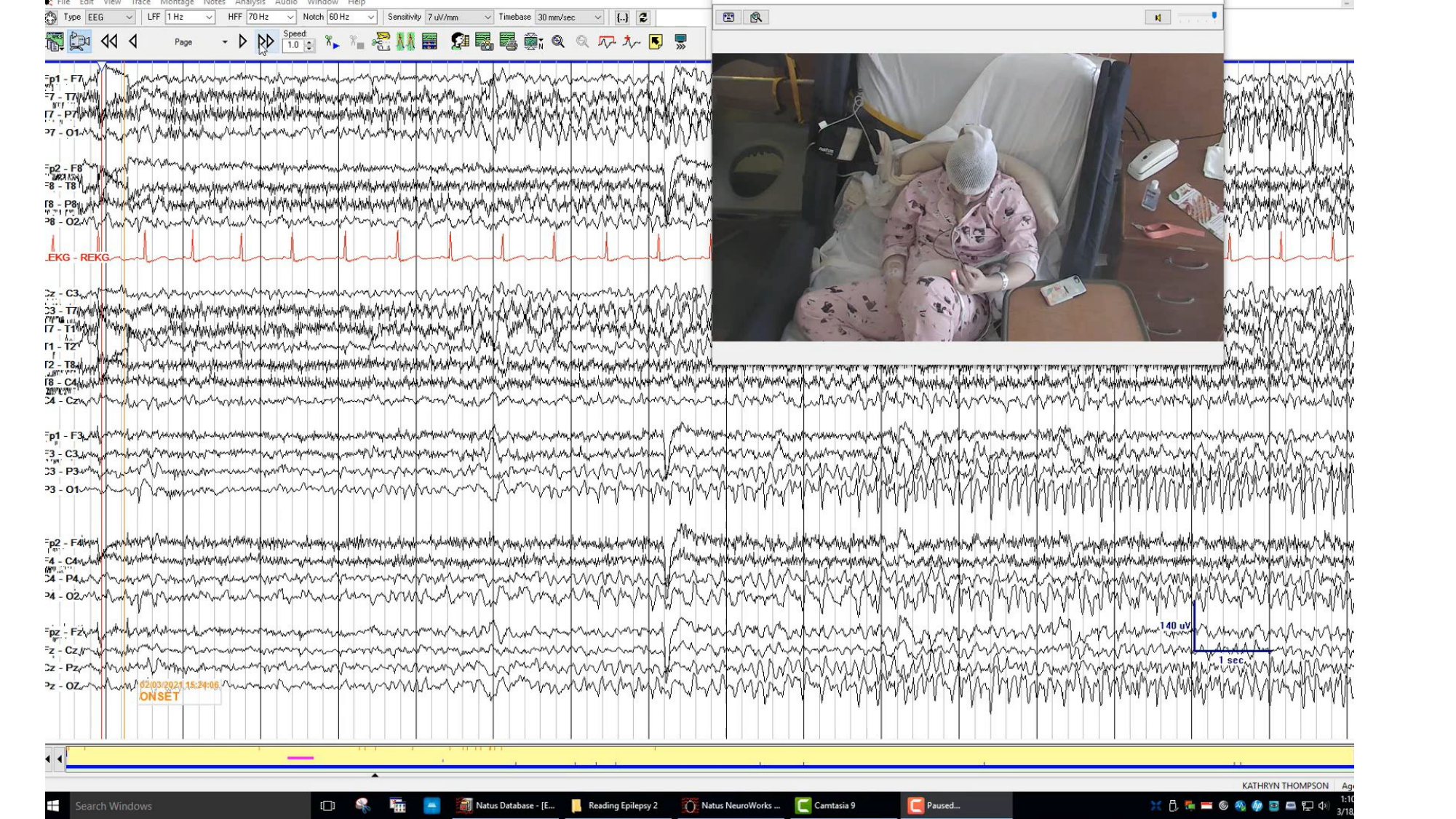

Supplement: Supplementary file 22 — Data S22. [file EPD2-27-1087-s021.pptx]
