# Supplementary material for: A practical guide to the updated seizure classification 2025
Source: Epileptic Disord. 2025 Oct 13;27(6):1087–104. doi: 10.1002/epd2.70110 (PMC12747708; doi:10.1002/epd2.70110)
Supplement: Supplementary file 8 — Data S8. [file EPD2-27-1087-s010.pptx]

## Slide 1
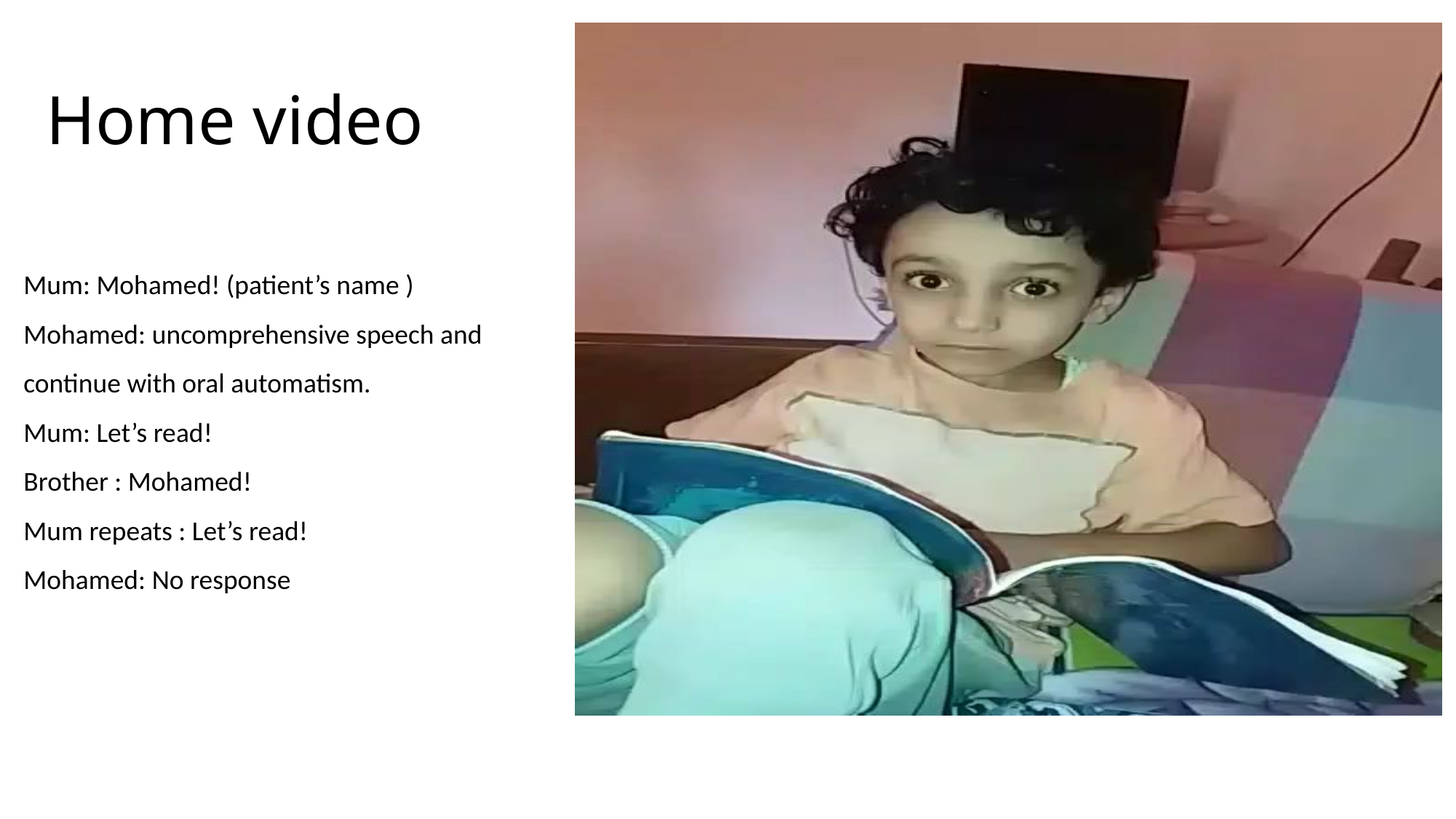

# Home video
Mum: Mohamed! (patient’s name )
Mohamed: uncomprehensive speech and continue with oral automatism.
Mum: Let’s read!
Brother : Mohamed!
Mum repeats : Let’s read!
Mohamed: No response

## Slide 2
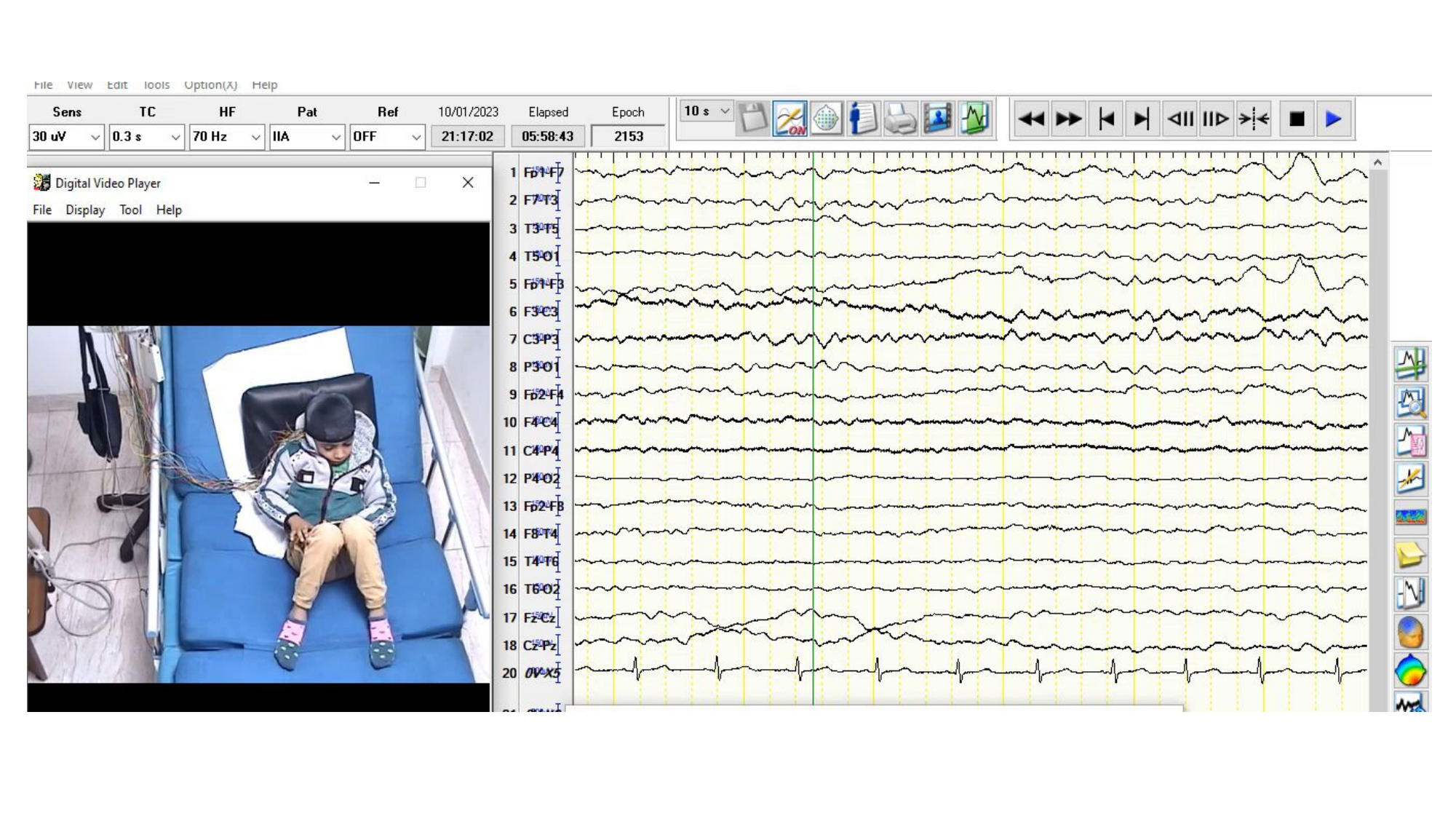

## Slide 3
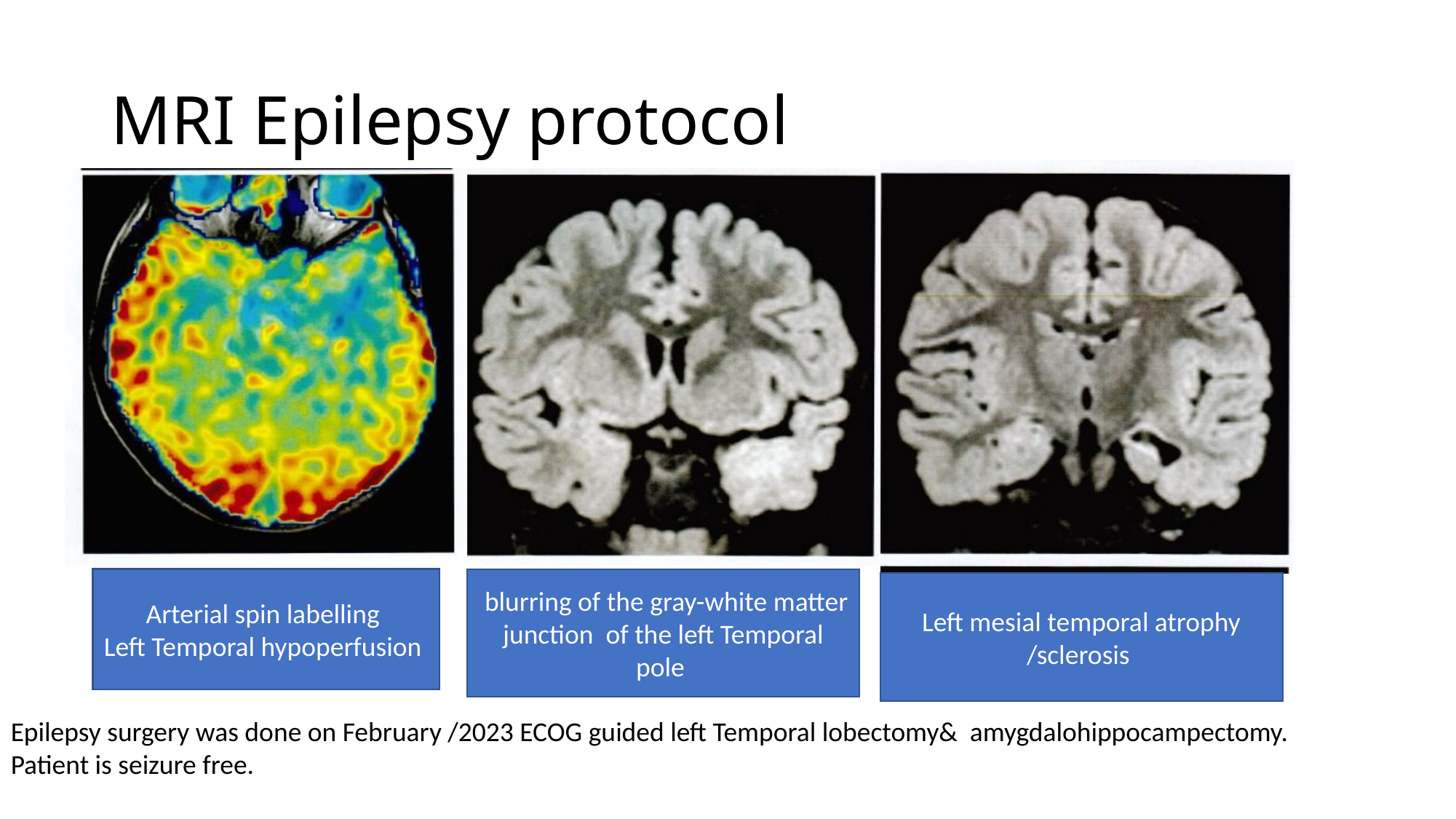

# MRI Epilepsy protocol
Arterial spin labelling
Left Temporal hypoperfusion
 blurring of the gray-white matter junction of the left Temporal pole
Left mesial temporal atrophy /sclerosis
Epilepsy surgery was done on February /2023 ECOG guided left Temporal lobectomy& amygdalohippocampectomy.
Patient is seizure free.
