# Supplementary material for: A practical guide to the updated seizure classification 2025
Source: Epileptic Disord. 2025 Oct 13;27(6):1087–104. doi: 10.1002/epd2.70110 (PMC12747708; doi:10.1002/epd2.70110)
Supplement: Supplementary file 9 — Data S9. [file EPD2-27-1087-s025.pptx]

## Slide 1
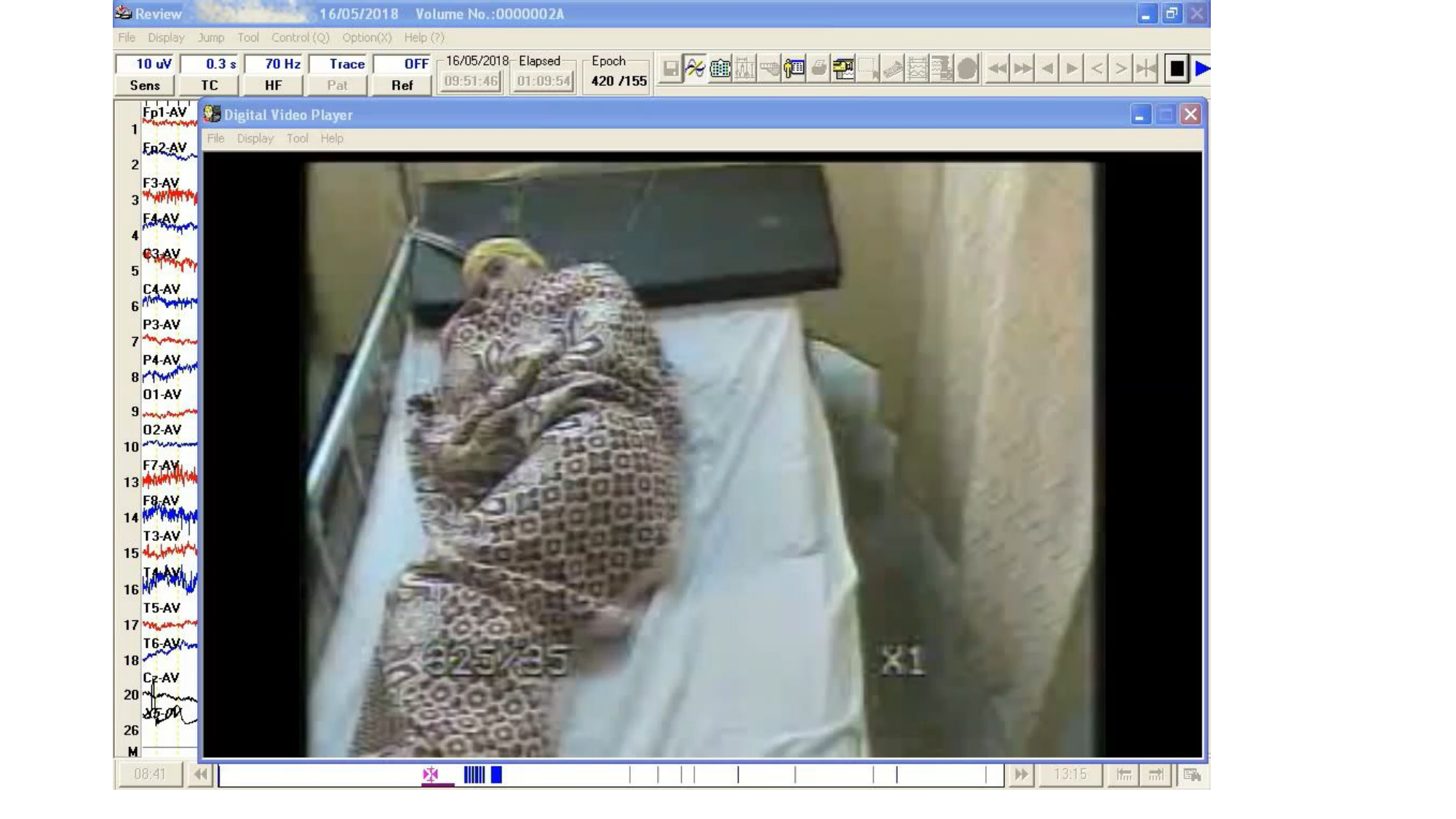

## Slide 2
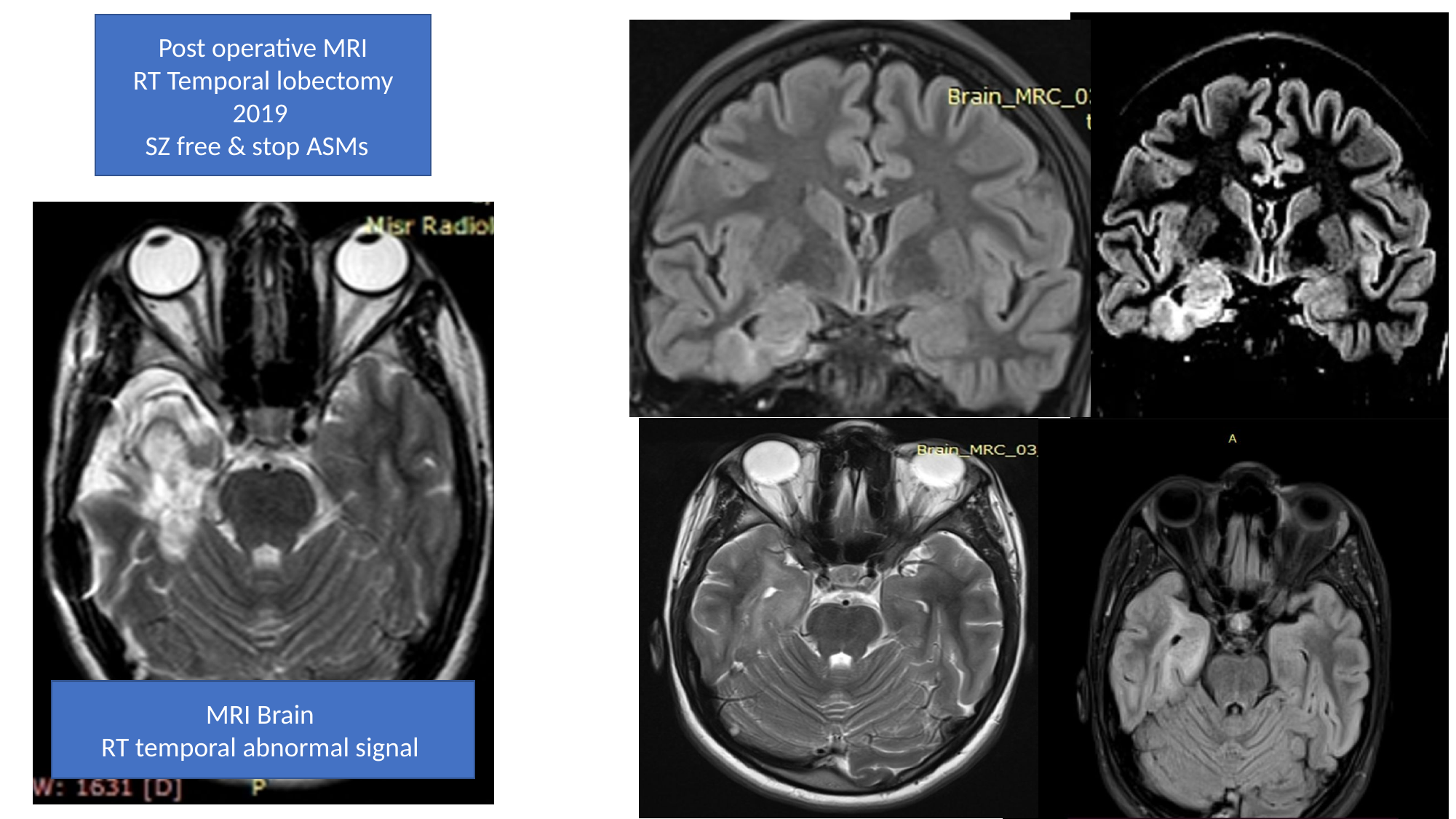

Post operative MRI
RT Temporal lobectomy 2019
SZ free & stop ASMs
MRI Brain
RT temporal abnormal signal
