# Supplementary material for: A practical guide to the updated seizure classification 2025
Source: Epileptic Disord. 2025 Oct 13;27(6):1087–104. doi: 10.1002/epd2.70110 (PMC12747708; doi:10.1002/epd2.70110)
Supplement: Supplementary file 10 — Data S10. [file EPD2-27-1087-s023.pptx]

## Slide 1
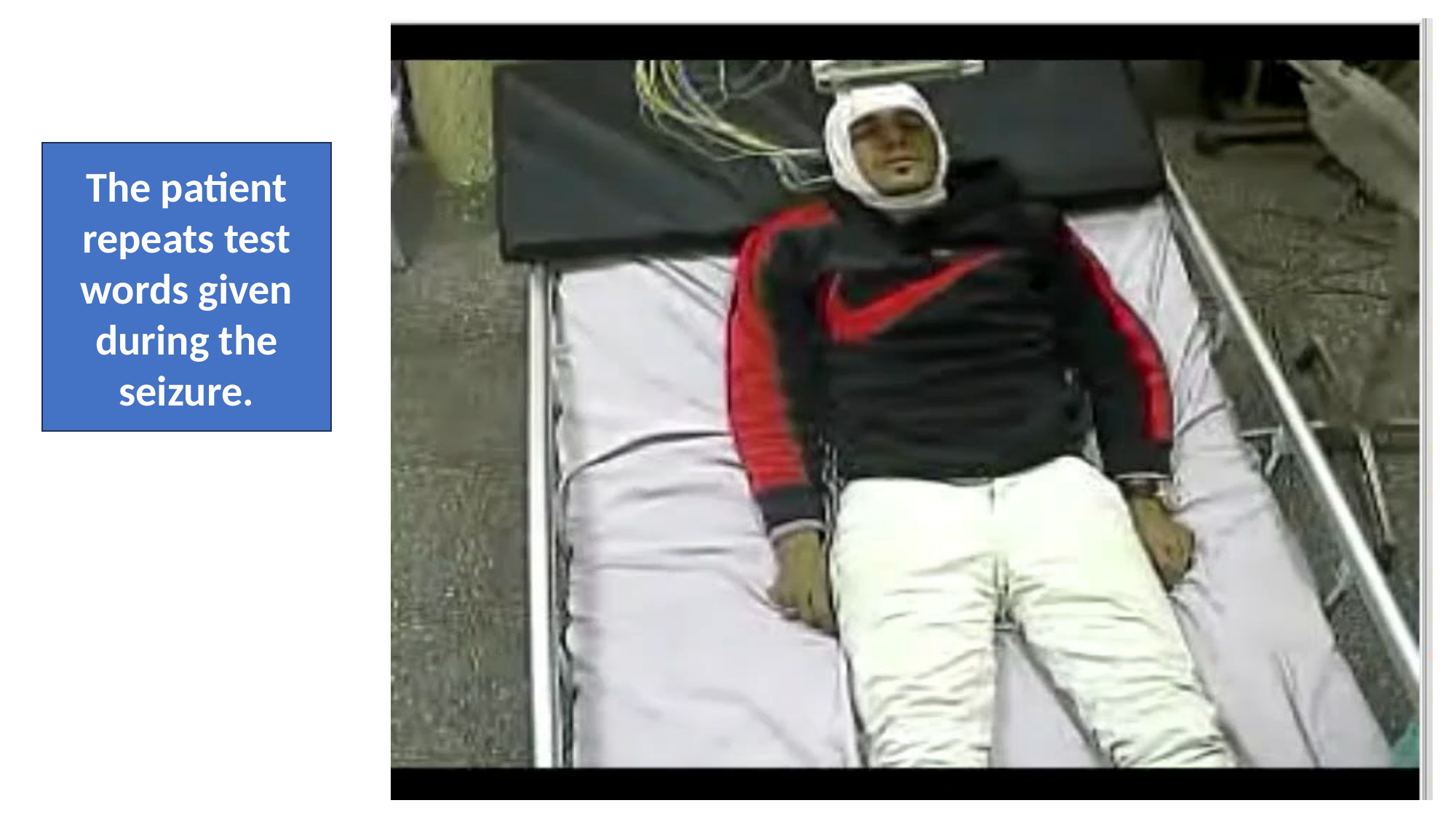

The patient repeats test words given during the seizure.

## Slide 2
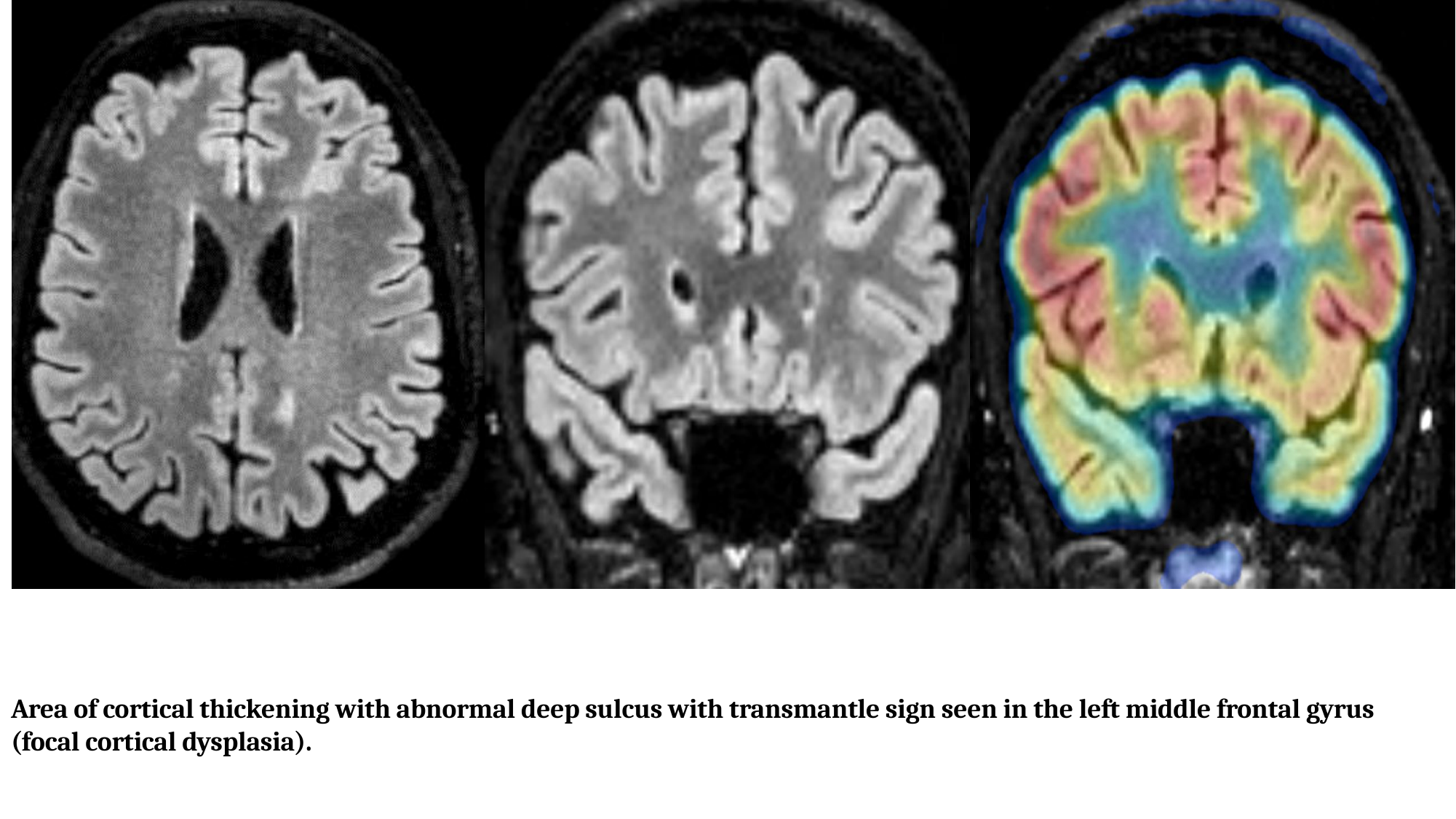

Area of cortical thickening with abnormal deep sulcus with transmantle sign seen in the left middle frontal gyrus (focal cortical dysplasia).
