# Supplementary material for: A practical guide to the updated seizure classification 2025
Source: Epileptic Disord. 2025 Oct 13;27(6):1087–104. doi: 10.1002/epd2.70110 (PMC12747708; doi:10.1002/epd2.70110)
Supplement: Supplementary file 16 — Data S16. [file EPD2-27-1087-s022.pptx]

## Slide 1
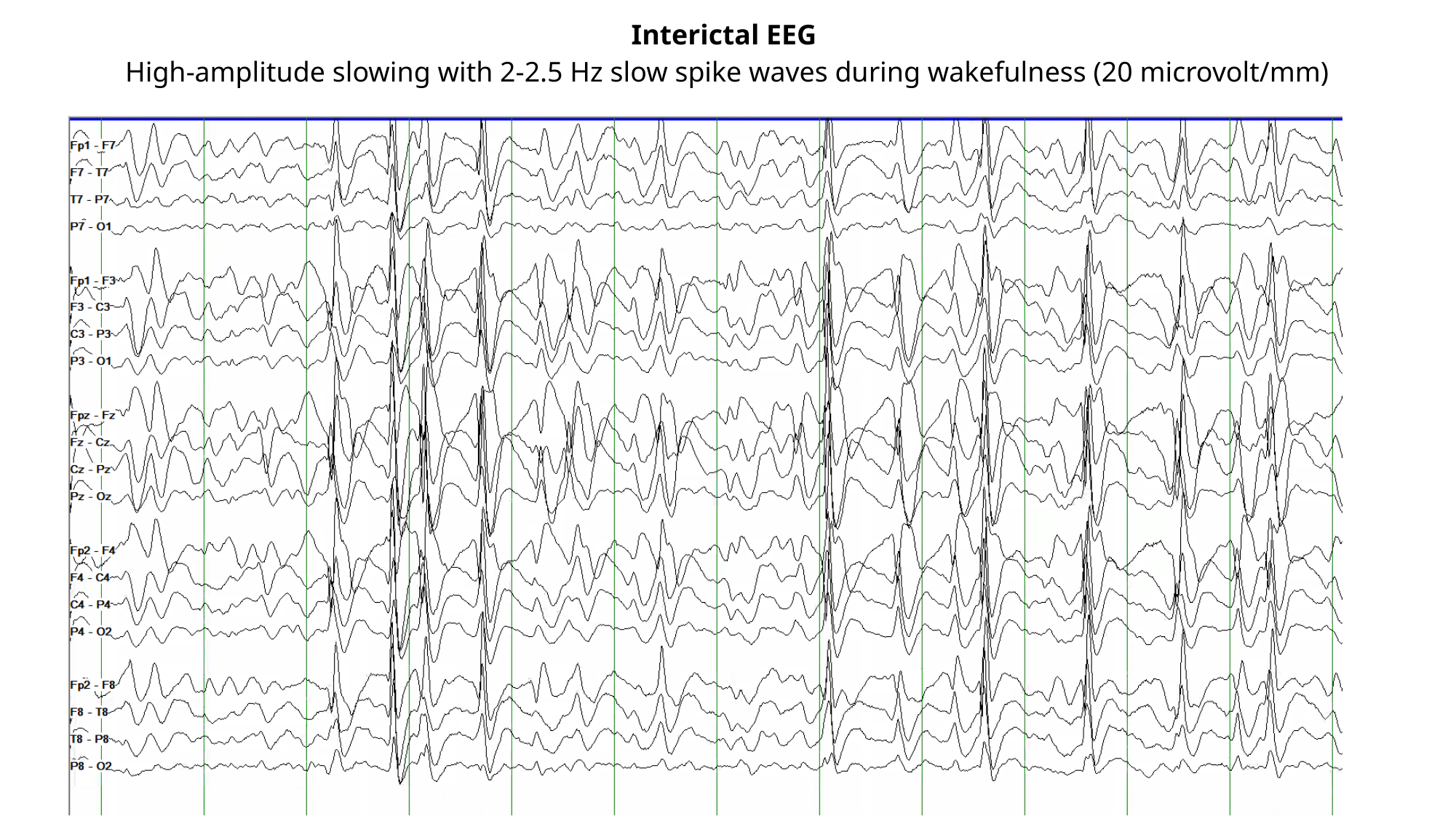

# Interictal EEG High-amplitude slowing with 2-2.5 Hz slow spike waves during wakefulness (20 microvolt/mm)

## Slide 2
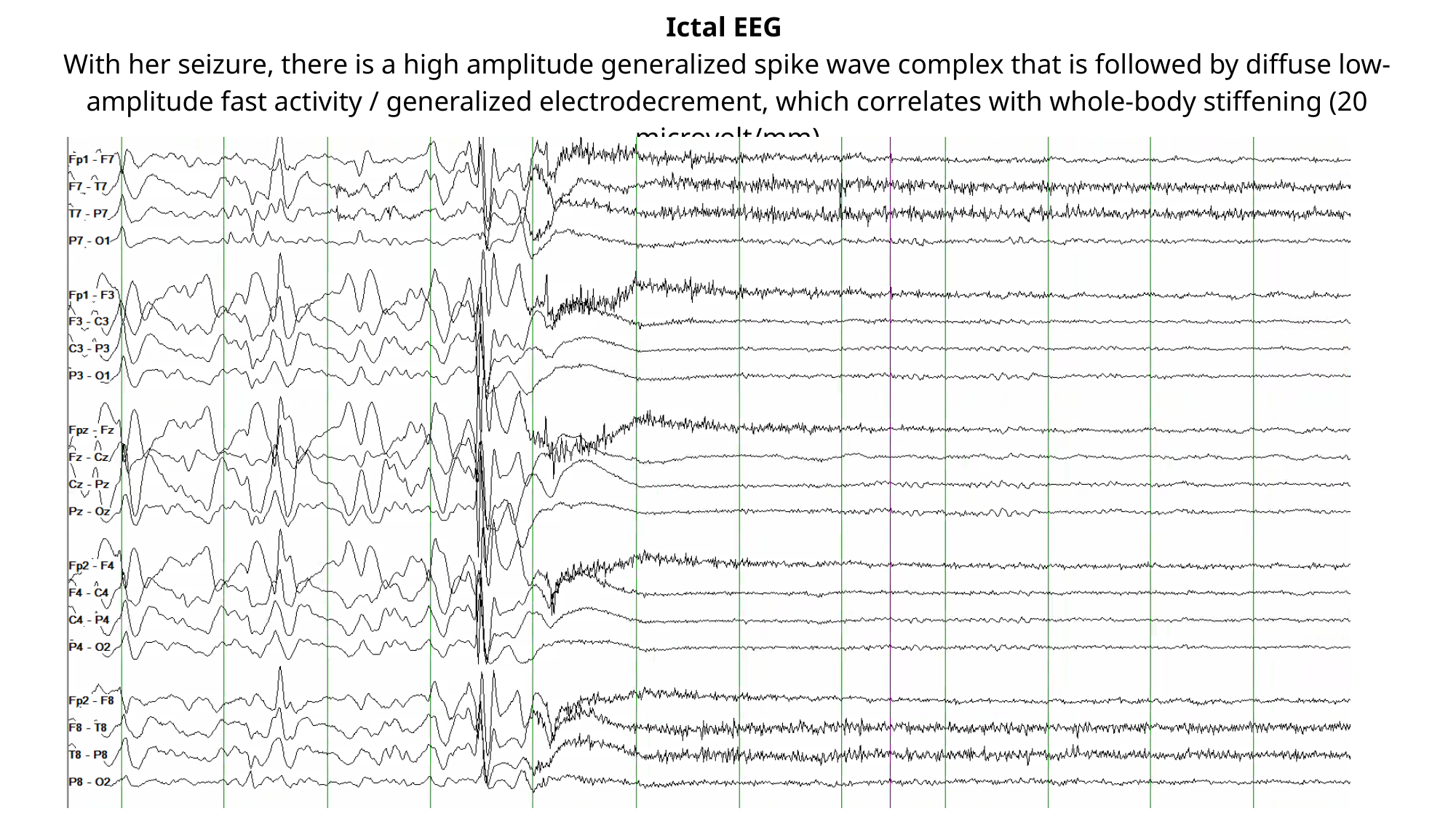

# Ictal EEG With her seizure, there is a high amplitude generalized spike wave complex that is followed by diffuse low-amplitude fast activity / generalized electrodecrement, which correlates with whole-body stiffening (20 microvolt/mm)

## Slide 3
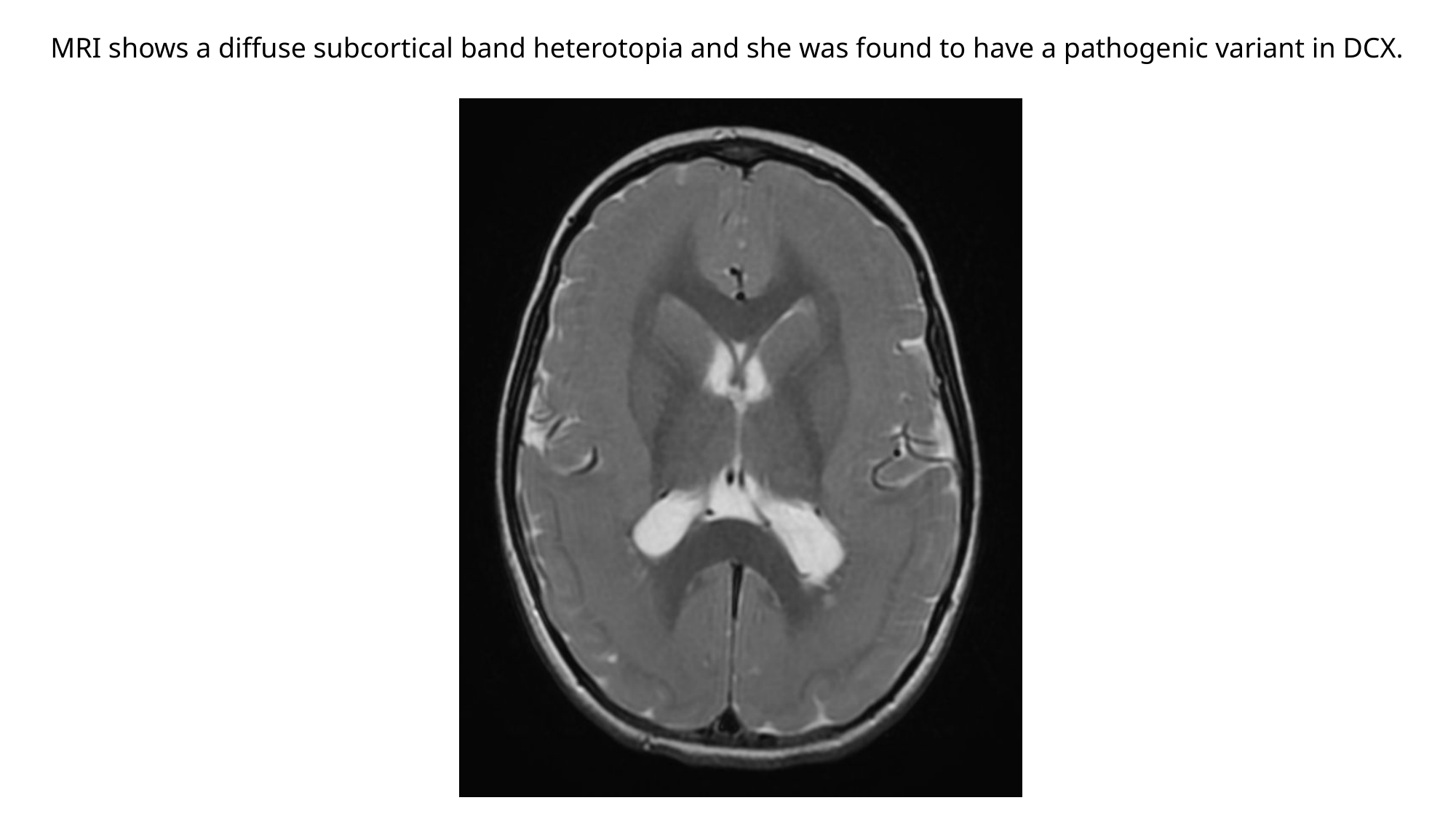

# MRI shows a diffuse subcortical band heterotopia and she was found to have a pathogenic variant in DCX.
