# Supplementary material for: A practical guide to the updated seizure classification 2025
Source: Epileptic Disord. 2025 Oct 13;27(6):1087–104. doi: 10.1002/epd2.70110 (PMC12747708; doi:10.1002/epd2.70110)
Supplement: Supplementary file 26 — Data S26. [file EPD2-27-1087-s020.pptx]

## Slide 1
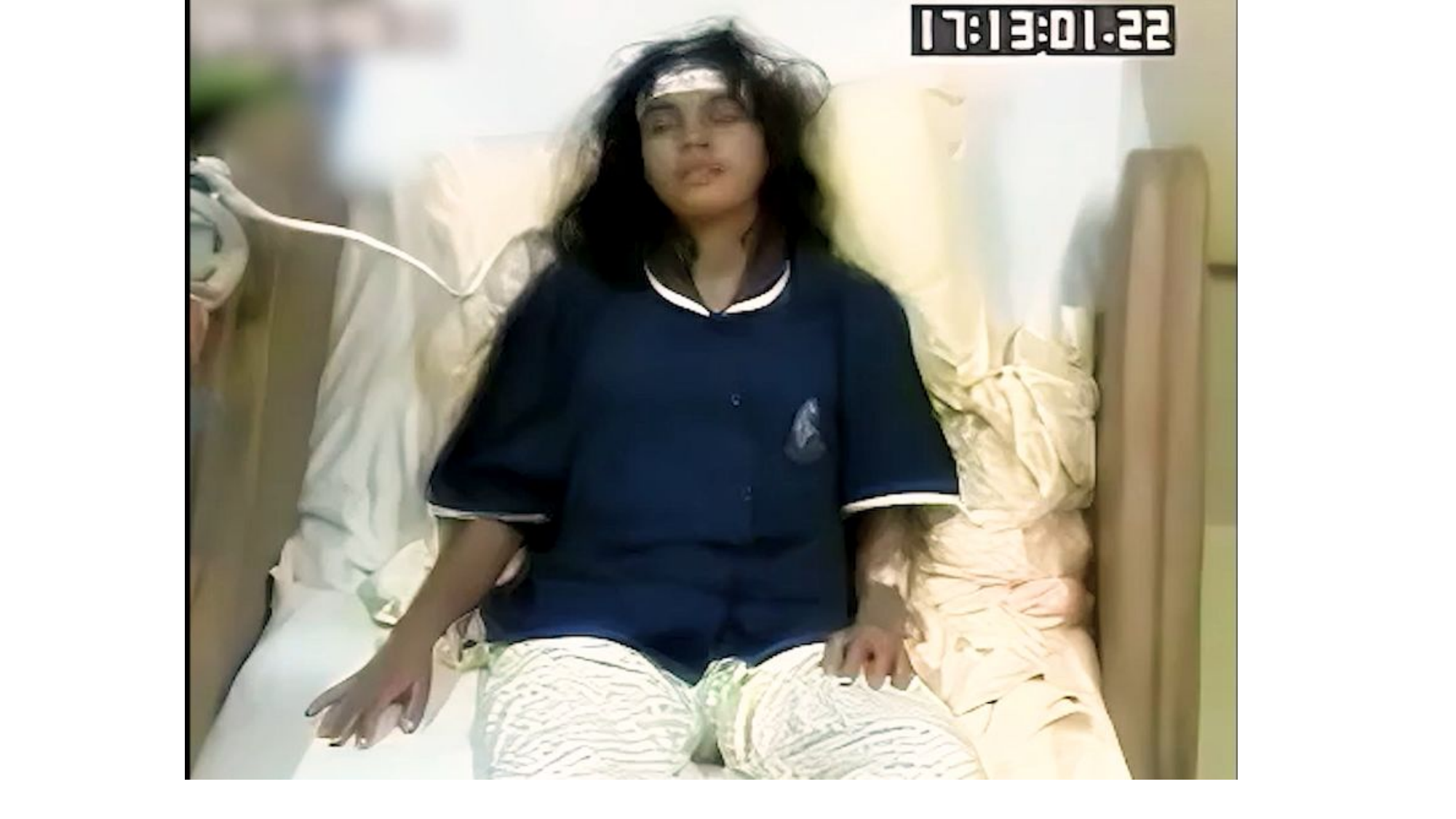

## Slide 2
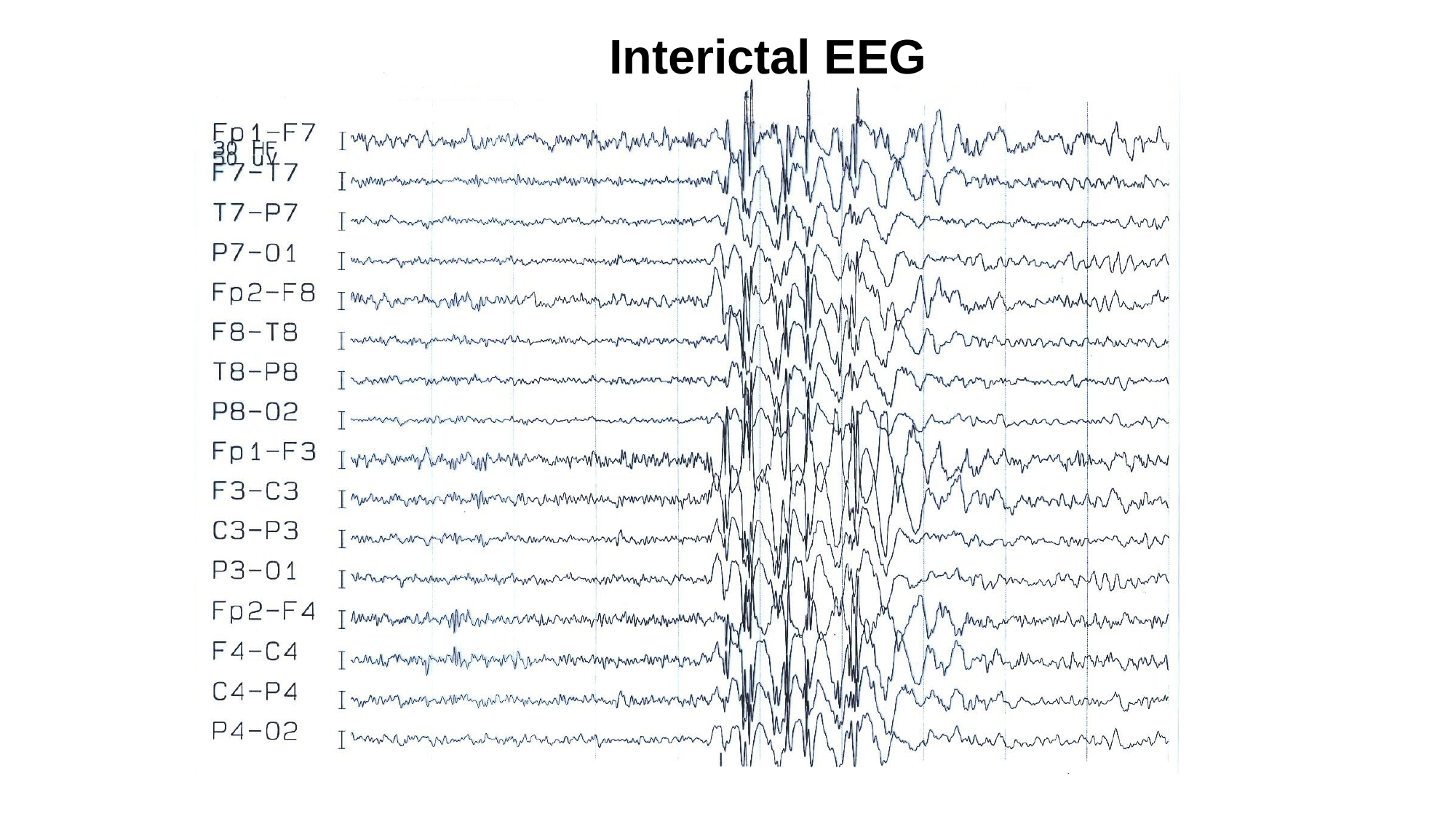

Interictal EEG

## Slide 3
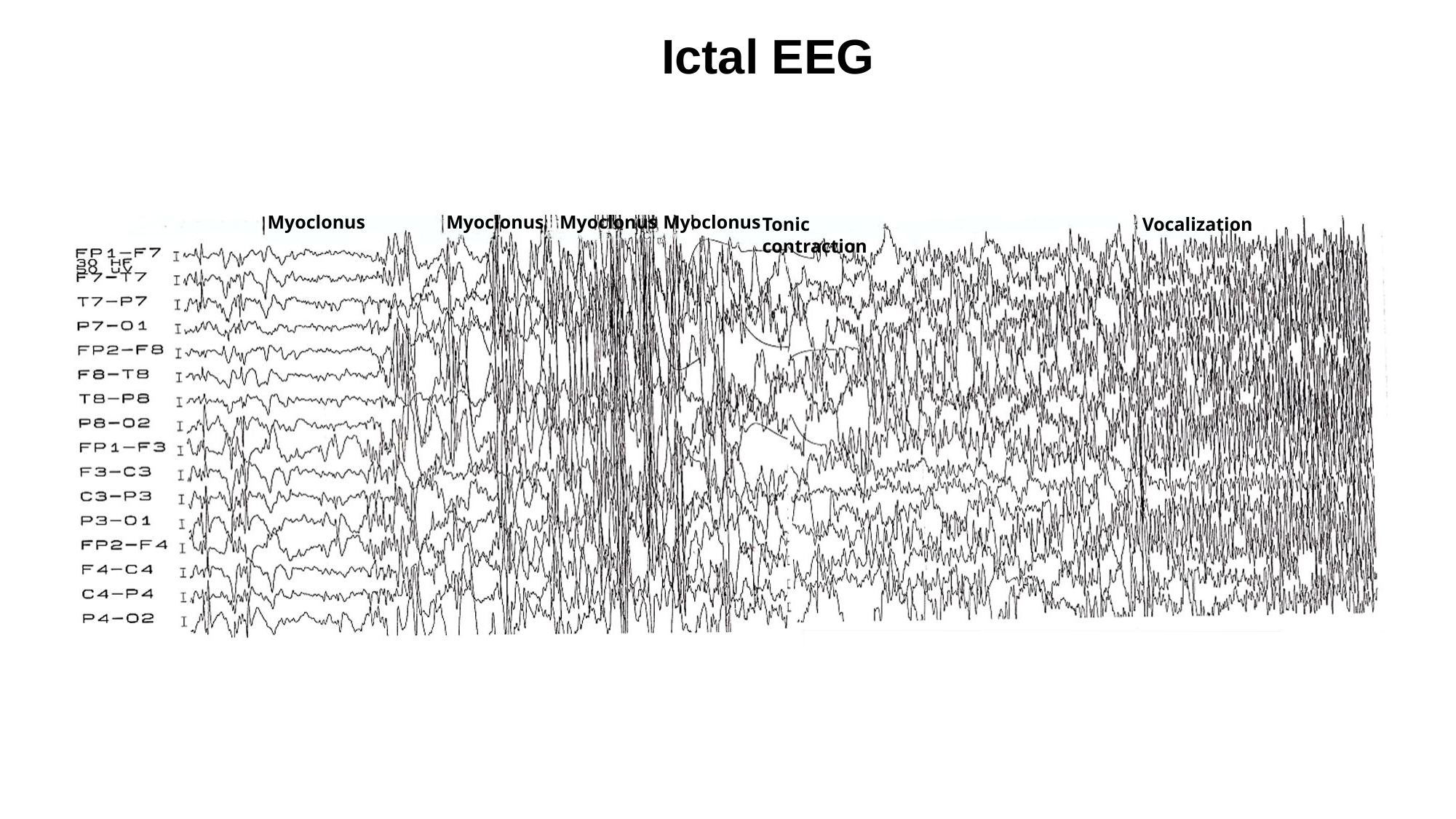

Ictal EEG
Myoclonus
Myoclonus
Myoclonus
Myoclonus
Vocalization
Tonic contraction
